# One-Pot Synthesis and Applications of N-Heteroaryl Iodonium Salts

**DOI:** 10.1002/open.201300042

**Published:** 2014-01-23

**Authors:** Marcin Bielawski, Joel Malmgren, Leticia M Pardo, Ylva Wikmark, Berit Olofsson

**Affiliations:** [a] Department of Organic Chemistry, Stockholm University,106 91 Stockholm (Sweden)

**Keywords:** arylation, heterocycles, hypervalent iodine, iodonium salts, oxidation

## Abstract

An efficient one-pot synthesis of N-heteroaryl iodonium triflates from the corresponding N-heteroaryl iodide and arene has been developed. The reaction conditions resemble our previous one-pot syntheses, with suitable modifications to allow N-heteroaryl groups. The reaction time is only 30 min, and no anion exchange is required. The obtained iodonium salts were isolated in a protonated form, these salts can either be employed directly in applications or be deprotonated prior to use. The aryl groups were chosen to induce chemoselective transfer of the heteroaryl moiety to various nucleophiles. The reactivity and chemoselectivity of these iodonium salts were demonstrated by selectively introducing a pyridyl moiety onto both oxygen and carbon nucleophiles in good yields.

Diaryliodonium salts have recently been recognized as efficient electrophilic arylation reagents with a wide range of nucleophiles under both metal-free and metal-catalyzed conditions.[1] The accessibility of these reagents has been greatly simplified by the recent development of efficient one-pot syntheses from the corresponding iodoarenes or iodine with arenes (Scheme 1).[2] A complimentary regiospecific route employing arylboronic acids gives access to diaryliodonium salts with all kinds of substitution patterns (Scheme 1).[3]

**Scheme 1:**
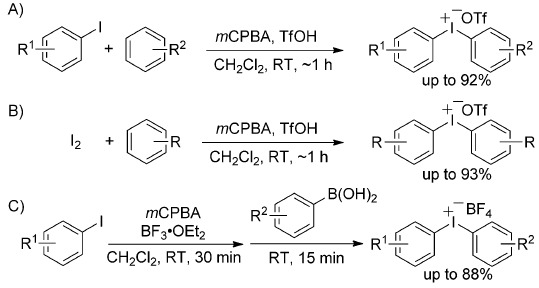
Our one-pot syntheses of Ar_2_IX from A) the corresponding iodoarenes or B) iodine with arenes or C) employing arylboronic acids.

Despite the wide scope of the one-pot methods described above, they fail in the synthesis of N-heterocyclic iodonium salts.[4] N-heterocycles are common organometallic ligands and substructures in biologically active compounds,[5] and the introduction of such a moiety from a diaryliodonium salt would be of high utility in organic synthesis. Symmetric N-heterocyclic salts can be obtained in low yields by treatment of a highly unstable vinyliodonium dichloride[6] with aryl lithium reagents at −78 °C.[7] Unsymmetric salts can be obtained in a similar way.[8] A four-step synthesis to 3-pyridyl(aryl)iodonium salts via the corresponding 3-pyridyl-iododichloride has also been reported.[9] The poor accessibility to N-heterocyclic diaryliodonium salts inspired us to develop a general one-pot synthesis of these compounds. The synthesis and chemoselective application of these salts to different nucleophiles are reported herein.

The reaction of 3-iodopyridine (**1 a**) with benzene (**2 a**) to give pyridyl iodonium salt **3 a** was chosen as a model system (Scheme 2). Initial attempts to form **3 a** under our standard conditions, 1.1 equiv *meta*-chloroperoxybenzoic acid (*m*CPBA) and 3 equiv trifluoromethanesulfonic acid (TfOH),[2b] resulted in mixtures due to competing N-oxidation of **1 a**.[10] This could be prevented by treating **1 a** with TfOH before addition of *m*CPBA, thus protecting the nitrogen from oxidation. The nitrogen remained protonated in the formed iodonium salt, and the isolated product was found to be phenyl(3-pyridinium)iodonium bistriflate (**3 a′**) rather than **3 a**.

**Scheme 2:**
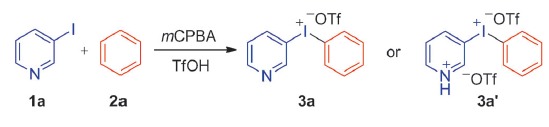
Model system.

The reaction conditions were further optimized, as detailed in Table 1. The amount of *m*CPBA was first investigated, and a slight excess proved better than equimolar amounts (Entries 1–3). Increasing the amount of TfOH from three to four equivalents further improved the yield, which can be explained by one equivalent of acid being consumed for nitrogen protonation (Entry 4). Subsequently, the reaction time and temperature were varied, and 30 min at 60 °C was as efficient as 3 h at 80 °C, while lower temperatures were insufficient (Entries 4–8). The high temperature required could be explained by slow I-oxidation of protonated **1 a**.

**Table 1:** Optimization of reaction conditions.[a]

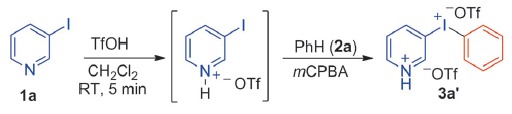					
Entry	*m*CPBA [equiv]	TfOH [equiv]	*T* [°C]	*t* [min]	Yield [%]
1	1.1	3.0	80	180	54
2	1.5	3.0	80	180	60
3	2.0	3.0	80	180	52
4	1.5	4.0	80	180	68
5	1.5	4.0	80	10	48
6	1.5	4.0	80	30	60
7	1.5	4.0	60	30	69
8	1.5	4.0	40	30	–[b]

[a] [a] 1.1 equiv **2 a** was used. The product was isolated by concentration of the crude mixture in vacuo followed by precipitation by addition of Et_2_O.

[b] Product isolation was difficult.

It is known that pyridyl(phenyl)iodonium salts provide insufficient chemoselectivity in reactions with nucleophiles under metal-free conditions.[9], [11] Therefore, further optimization was performed with anisole (**2 b**), which is known to be a useful “dummy group” in reactions of diaryliodonium salts with various nucleophiles.[12]

Syntheses of diaryliodonium salts with electron-rich arenes generally require special conditions, such as a milder acid or oxidant, to avoid side reactions. We have previously reported modified procedures in our one-pot syntheses, where the iodoarene is oxidized to the iodine(III) intermediate before addition of the electron-rich arene at low temperature.[2b], [3]

Because oxidation of protonated **1 a** is slow, it was deemed unsuitable to change the oxidation conditions. Thus, **1 a** was oxidized under the same conditions, followed by addition of 2 equiv water at 0 °C to quench the triflic acid and create a milder acid. The electron-rich arene in dichloromethane was added dropwise, and additional stirring for 10 min afforded the desired product **3 b′** in high yield (Scheme 3).

**Scheme 3:**
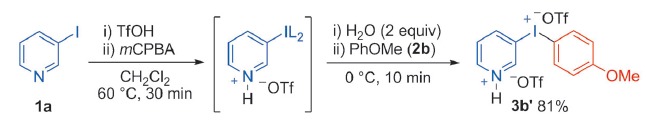
Modified synthesis with electron-rich arenes.

Pyridinium bistriflate **3′** might behave similarly to pyridyl salts **3** in applications, but it was desirable to also have access to deprotonated salts **3**. A deprotonation procedure was therefore developed to remove the triflic acid from **3′** to obtain pyridyl salts **3**. Several basic workup procedures were attempted to isolate salt **3 b′**, but unwanted anion exchanges complicated such deprotonations.[13] Deprotonation was best achieved using a basic Al_2_O_3_ column eluted with dichloromethane/methanol (20:1). The eluted material was concentrated in vacuo to give the pure deprotonated product **3 b** in 97 % yield. Unfortunately, submitting the crude reaction mixture directly onto an Al_2_O_3_ column gave back the protonated material, and isolation of **3′** before deprotonation was necessary.

The optimized synthesis of **3′** and deprotonation to **3** was subsequently applied on a range of N-heterocyclic iodoarenes **1** and arenes **2**. The arenes were selected to give good chemoselectivity in both metal-free and metal-mediated reactions (Scheme 4). As mentioned above, the anisyl moiety is a good dummy group in many reactions under metal-free conditions.[12] Sterically hindered groups, such as mesityl and 2,4,6-triisopropylphenyl (TRIP) are useful in metal-mediated reactions and in arylations with malonates.[12], [14]

**Scheme 4:**
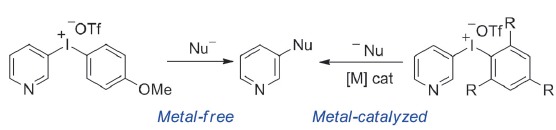
General chemoselectivity trends.

The selected heteroaryl salts were synthesized using the two optimized methods described above, and selected salts were deprotonated to illustrate the methodology (Scheme 5). 3-Iodopyridine (**1 a**) was combined with a range of arenes to give heteroaryl salts **3 a**–**e**. Salts **3 b**–**d** have the selected dummy groups for chemoselective arylations under different conditions.

**Scheme 5:**
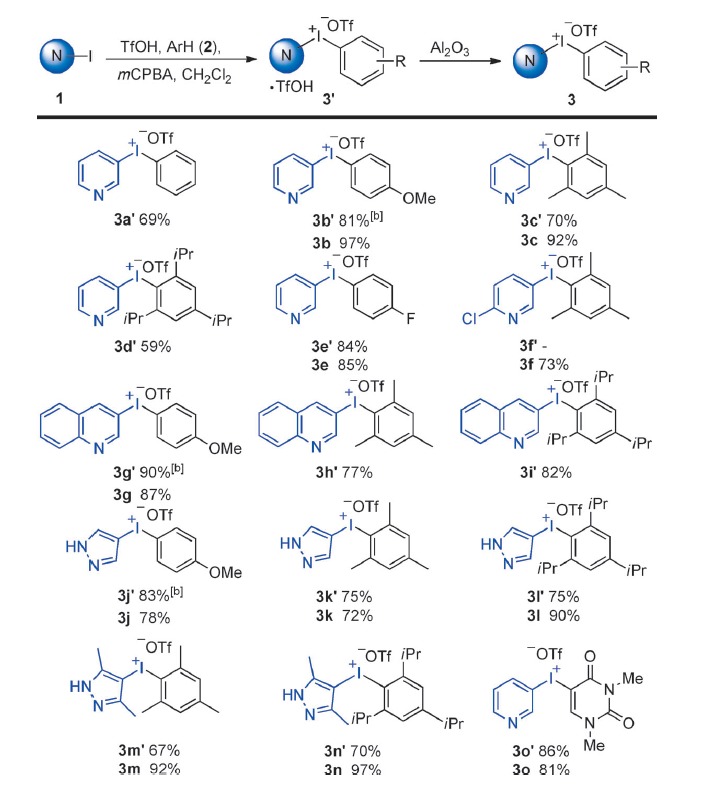
Synthesized N-heteroaryl iodonium salts. Method A was used unless specified (see Table 1, Entry 7). Yield of 3 is from isolated 3′. [a] Method B was used (see Scheme 3).

2-Chloro-5-iodopyridine (**1 b**) was reacted with mesitylene to directly deliver salt **3 f** without the need for deprotonation. Apparently, the electron-withdrawing chloride diminishes the basicity of the nitrogen enough to avoid both N-oxidation and protonation, which was previously noticed in iodonium salt syntheses with **1 b** and benzene or anisole.[2b], [4] 3-Iodoquinoline (**1 c**) was used to synthesize salts **3 g**–**i**, with all three dummy groups. 4-Iodo-1*H*-pyrazole (**1 d**) and 3,5-dimethyl-4-iodo-1*H*-pyrazole (**1 e**) could also be utilized in this reaction, forming salts **3 j**–**n** with the selected dummy groups.

Aryl(uracyl)iodonium salts[15] have recently been applied in the preparation of heteroaryl ketones using an N-heterocyclic carbene (NHC) catalyst.[16] While chemoselective, the cost of *N*,*N*-dimethyluracil makes this dummy group less attractive in standard reactions.[17] Still, uracyl salt **3 o** could be synthesized in high yield with this methodology (see Scheme 5).

The synthesis of salts **3′** was generally high yielding, and reactions with the anisyl dummy group consistently gave better yields than the alkyl-substituted arenes. The modified procedure used for anisyl salts was inefficient for synthesis of salts with less electron-rich character. Also, the deprotonation of **3’** to **3** took place in good to excellent yields. The salts with an anisyl moiety (**3 b, g, j)** are expected to chemoselectively deliver the pyridyl group in metal-free reactions with nucleophiles, and salts with a mesityl (**3 c, f, h, k, m**) or TRIP group (**3 d, i, l, n**) should be chemoselective in metal-catalyzed reactions and other reactions sensitive to steric hindrance, for example, malonate arylations.

A reliable analytical tool was required to ensure that complete deprotonation had taken place for all salts. While HRMS delivered the same molecular weight for both **3′** and **3**, the ^1^H NMR spectra in deuterated methanol differed. A concentration effect with slightly changing shifts is seen in samples of **3′**, due to the interchangeable proton, while shifts of **3** are constant.[13] Salt **3 e′** was synthesized to confirm the accuracy of this analytical method. Apart from the differences in ^1^H NMR spectra described above, ^19^F NMR analysis of **3 e′** gave a ratio of 6:1 between the two detected peaks, indicating the presence of two triflate moieties. The same analysis after deprotonation to **3 e** indeed gave a ratio of 3:1.

Application studies were next undertaken to demonstrate the utility of the prepared salts in chemoselective arylations. Furthermore, the reactivities of the protonated salts **3′** and the deprotonated salts **3** were compared. The electron-rich salts **3 b′** and **3 b** were applied in the arylation of phenols, using our previously reported protocol.[18] Arylation of **4 a** with **3 b** gave pyridyl ether **5 a** in 88 % yield (Scheme 6). The sterically hindered phenol **4 b** was also arylated to give **5 b**, albeit in slightly lower yield. Reactions with salt **3 b′** were performed with 2.1 equiv base instead of 1.1 equiv to compensate for the TfOH present.[19] The yields with this salt were lower than with **3 b**; but considering the facile isolation of **3 b′**, it might still be a useful alternative in some applications. The reactivity of N-heteroaryl salts **3 c** and **3 c′** was investigated by arylation of diethyl methylmalonate (**6**),[20] which has previously been chemoselectively arylated using a mesityl dummy group.[12] Again, the deprotonated salt was more efficient than the protonated salt in formation of **7**, despite use of 2 equiv base (Scheme 7). Complete chemoselectivity was obtained in all reactions, illustrating the usefulness of inexpensive anisole and mesitylene as dummy groups in these arylations.

**Scheme 6:**
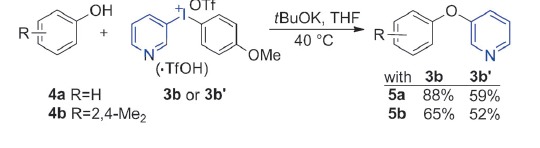
Chemoselective arylation of phenols.

**Scheme 7:**
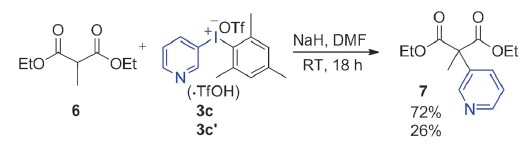
Chemoselective arylation of malonate 6.

An efficient synthesis of N-heteroaryl iodonium triflates has been developed. The aryl groups were chosen to induce chemoselective transfer of the heteroaryl moiety to different nucleophiles. This was demonstrated by selectively introducing a pyridyl moiety onto both oxygen and carbon nucleophiles. The protonated heteroaryl salts **3′** give lower yields than the deprotonated salts **3** in these reactions but might be as efficient in transformations that are not base mediated.

## Experimental Section

### General experimental procedures

**Method A**: Iodoarene **1** (0.24 mmol) was stirred with TfOH (4 equiv) in CH_2_Cl_2_ (1 mL) for 5 min at RT. Arene **2** (1.1 equiv) and *m*CPBA (1.5 equiv) were added, and the solution was stirred at 60 °C for 30 min.

**Method B**: Iodoarene **1** (1.51 mmol) was stirred with TfOH (4 equiv) in CH_2_Cl_2_ (10 mL) for 5 min at RT. *m*CPBA (1.75 equiv) was added, and the solution was stirred at 60 °C for 30 min. After H_2_O (2 equiv) was subsequently added at 0 °C and then arene **2** (1.1 equiv), the solution was stirred at 0 °C for 10 min.

**Isolation of 3′**: The crude mixture was concentrated in vacuo followed by precipitation by addition of Et_2_O (1–3 mL). The solid was collected by filtration and washed with Et_2_O.

**Deprotonation to 3**: Isolated **3’** was added on a basic Al_2_O_3_ column and eluted with CH_2_Cl_2_/MeOH (20:1). The eluted mixture was concentrated in vacuo to give the pure deprotonated product **3**.
