# Supplementary material for: One-Pot Synthesis and Applications of N-Heteroaryl Iodonium Salts
Source: ChemistryOpen. 2014 Jan 23;3(1):19–22. doi: 10.1002/open.201300042 (PMC3943608; doi:10.1002/open.201300042)
Supplement: Supplementary file 1 [file open0003-0019-sd1.pdf]

ChemistryOPEN

Supporting Information

© 2014 The Authors. Published by Wiley-VCH Verlag GmbH & Co. KGaA, Weinheim

One-Pot Synthesis and Applications of N-Heteroaryl Iodonium Salts

Marcin Bielawski, Joel Malmgren, Leticia M. Pardo, Ylva Wikmark, and Berit Olofsson*[a]

open_201300042_sm_miscellaneous_information.pdf

SUPPORTING INFORMATION

1	General Experimental Conditions	2
2	NMR Analysis of Concentration Effect and Deprotonation	2
3	Synthesis of *N*-Heteroaryliodonium Triflates 3' and 3	4
4	Arylation of Phenols 4 to diaryl ethers 5[4]	11
5	Arylation of Diethyl methylmalonate 6 to Product 7[6]	12
6	References	12
7	Copies of NMR spectra	12

1 General Experimental Conditions

Precautions to exclude air or moisture were not taken, except when mentioned. Commercial *m*CPBA was dried under vacuum at rt for 1 hour and subsequently the percentage of active oxidising reagent was determined by iodometric titration.[1] All other commercially available chemicals were used as received. For TLC analyses precoated silica gel 60 F_{254} plates were used; and for column chromatography 40-60 µm, 60A silica gel was used. Melting points were measured using a STUART SMP3 and are reported uncorrected. NMR spectra were recorded using a 400 or 500 MHz Bruker AVANCE II with a BBO probe at 298 K, using MeOH-d_4 and $CDCl_3$ as solvents. Chemical shifts are given in ppm relative to the (residual) solvent peak (^{1}H NMR: $CDCl_3$ δ 7.26, MeOH-d_4 δ 3.31; ^{13}C NMR: $CDCl_3$ δ 77.23, MeOH-d_4 δ 49.0) with multiplicity (s = singlet, d = doublet, t = triplet, q = quartet, m = multiplet, app = apparent), coupling constants (in Hz) and integration. High-resolution mass analyses were obtained using a Bruker microTOF ESI.

2 NMR Analysis of Concentration Effect and Deprotonation

2.1 Concentration effects in the NMR Analysis of 3b'

Diaryliodonium salt **3b'** was analysed by NMR together with either an excess of TfOH (Figure 1, top, green) or Et_3N (Figure 1, bottom, orange) in order to obtain the spectrum for full protonation and deprotonation respectively. Different amounts of salt **3b'** in 0.5 mL of MeOH-d_4 were then run to find that their spectra fit between the two extremes of protonated and deprotonated **3b'** (Figure 1).

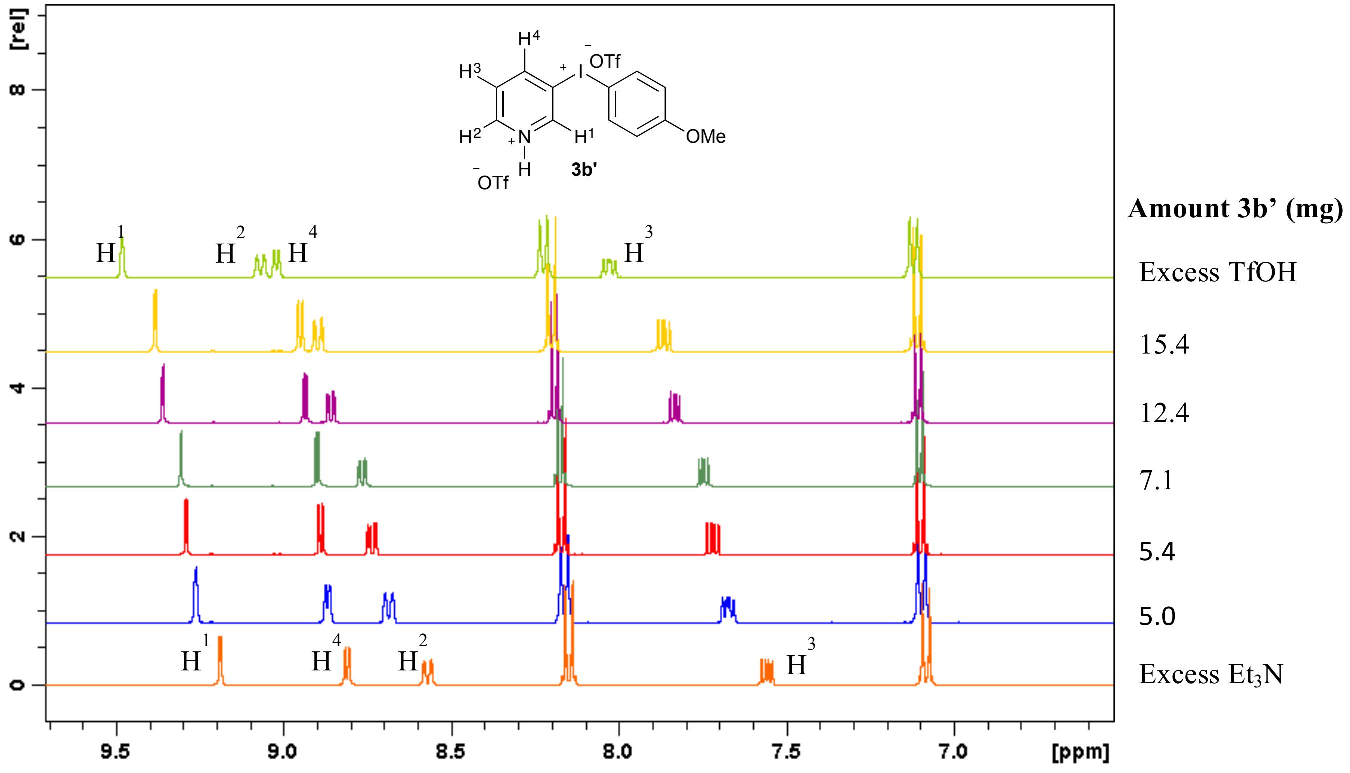

Figure S1. NMR spectra of different concentrations of 3b' in MeOH-d_4.

Due to the concentration effect described above, it is possible to distinguish between the two species **3'** and **3** by running two ^{1}H NMRs with different sample concentrations. If the shifts do not differ, the salt is deprotonated. A second NMR could also be run after the addition of a small amount of Et_3N. ^{19}F NMR with fluorobenzene as the internal standard can also be utilized. Compounds **3e'** and **3e** were synthesised in order to compare the ^{19}F-NMR ratio with an intramolecular internal standard. As expected, the integral ratio between the two peaks were 1:6 and 1:3 respectively.

2.2 Deprotonation of Products 3' to 3

To a column (Ø = 1.5 cm) was added cotton (h ≈ 0.5 cm, packed) followed by addition of sand (h = 0.5 cm). CH_2Cl_2 was then added to the column followed by basic Al_2O_3 (1.5 g). Salt **3'** (0.24 mmol) dissolved in CH_2Cl_2 (0.5 mL) and MeOH (0.1 mL) was then applied on the column. The column was eluted with a mixture of CH_2Cl_2 and MeOH (20:1, 40 mL), and the eluted liquids were concentrated *in vacuo* to give the desired deprotonated salt **3**. *NB*: When scaling up, use the same height of Al_2O_3 as in the described procedure by using a broader column.

Screened Deprotonation Methods

- Addition of $NaHCO_3$ or NaOAc as solids or in solution to the crude material was inefficient.
- Purification by silica with a NH_3 (aq. 25%) in the eluent worked, but NH_4OTf eluted together with product **3b**.
- Addition of Et_3N to **3b'** in CH_2Cl_2 followed by evaporation *in vacuo* and precipitation with Et_2O gave the deprotonated product **3b** in 67% yield, which was difficult to reproduce.

3 Synthesis of *N*-Heteroaryliodonium Triflates 3' and 3

3.1 Method A

To a solution of heteroaryl iodide **1** (0.24 mmol) in CH_2Cl_2 (1 mL) was added TfOH (4 equiv) and the resulting mixture was stirred at rt for 5 min. *m*CPBA (1.5 equiv) followed by the appropriate arene (1.1 equiv) was then added. The reaction vessel was sealed and submitted to a 60 °C oilbath with stirring for 30 min. The reaction mixture was then allowed to reach rt after which it was concentrated *in vacuo*. Et_2O (1 mL) was added and the mixture was stirred at 0 °C for 30 min. The resulting precipitate was filtered through a glass-sintered funnel and washed with additional Et_2O (3 x 1 mL) to give the protonated *N*-heteroaryliodonium bistriflate **3'**.

3.2 Method B (for 3b',3g', 3j')

To a solution of heteroaryl iodide **1** (1.51 mmol) in CH_2Cl_2 (10 mL) was added TfOH (4 equiv) and the resulting mixture was stirred at rt for 5 min. *m*CPBA (1.75 equiv) was then added. The reaction vessel was sealed and submitted to a 60 °C oilbath with stirring for 30 min after which it was cooled down to 0 °C. H_2O (2 equiv) was added, followed by the dropwise addition of the appropriate arene **2** (1.2 equiv) dissolved in CH_2Cl_2 (2 mL) *via* syringe. The reaction mixture was stirred for 15 min at 0 °C before it was concentrated *in vacuo*. Et_2O (1-3 mL) was added and the mixture was cooled to 0 °C and stirred for 30 min. The resulting precipitate was filtered through a glass sintered funnel and washed with additional Et_2O (3 x 3 mL) to give the protonated *N*-heteroaryliodonium bistriflate **3'**.

3.3 Analyctical Data

Phenyl(3-pyridinium)iodonium bistriflate 3a'

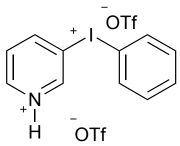

Synthesized according to Method A from **1a** and benzene (**2a**) to give **3a'** in 69% yield as a light grey solid; mp 127-130 °C; ^{1}H NMR (400 MHz, 5 mg in MeOH-d_4, (0.5 mL)) δ 9.29 (d, *J* = 1.6, 1H), 8.87 (dd, *J* = 4.9, 1.3, 1H), 8.71 (ddd, *J* = 8.3, 2.2, 1.3, 1H), 8.26 (appd, *J* = 8.5, 2H), 7.72 (appt, *J* = 7.5, 1H), 7.65 (ddd, *J* = 8.4, 5.0, 0.7, 1H), 7.57 (appt, *J* = 8.0, 2H); ^{13}C NMR (100 MHz, 20 mg in MeOH-d_4 (0.5 mL)): δ 152.3, 151.1, 147.2, 136.9, 134.2, 133.5, 129.2, 121.8 (q, J_{C-F} = 316), 116.3, 115.4; HRMS (ESI): *m/z* calculated for $C_{11}H_9NI$ ([M-HOTf-OTf$^-$])$^+$: 281.9774, found: 281.9759.

4-Methoxyphenyl(3-pyridinium)iodonium bistriflate 3b'

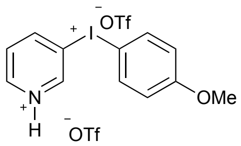

Synthesized according to Method B from **1a** and anisole (**2b**) to give **3b'** in 81% yield as an off-white solid; mp 181-183 °C; ^{1}H NMR (500 MHz, 12.4 mg in MeOH-d_4 (0.5 mL)) δ 9.36-9.35 (m, 1H), 8.94 (dd, *J* = 5.2, 1.3, 1H), 8.86 (ddd, *J* = 8.3, 2.2, 1.3, 1H), 8.19 (appd, *J* = 9.2, 2H), 7.83 (ddd, *J* = 8.3, 5.2, 0.7, 1H), 7.11 (appd, *J* = 9.2, 2H), 3.87 (s, 3H); ^{13}C NMR (125 MHz, 11.5 mg in MeOH-d_4 (0.5 mL)): δ 165.0,

151.4, 150.3, 147.6, 139.0, 129.3, 121.8 (q, $J_{C\text{-}F}$ = 317), 119.2, 115.7, 104.5, 101.4, 56.4; HRMS (ESI): *m/z* calculated for $C_{12}H_{11}NOI$ ([M-HOTf-OTf$^-$])$^+$: 311.9880, found: 311.9887.

4-Methoxyphenyl(3-pyridyl)iodonium triflate 3b

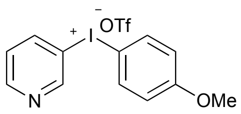

Isolated from **3b'** *via* deprotonation as an oily residue in 97% yield; ^{1}H NMR (400 MHz, MeOH-d_4) δ 9.19 (dd, *J* = 2.3, 0.5, 1H), 8.82 (dd, *J* = 4.8, 1.4, 1H), 8.56 (ddd, *J* = 8.4, 2.4, 1.4, 1H), 8.14 (appd, *J* = 9.2, 2H), 7.56 (ddd, *J* = 8.3, 4.8, 0.7, 1H), 7.09 (appd, *J* = 9.2, 2H), 3.86 (s, 3H); ^{13}C NMR (100 MHz, MeOH-d_4) δ 164.8, 154.3, 153.4, 143.6, 138.8, 128.2, 121.8 (q, $J_{C\text{-}F}$ = 316), 119.1, 116.1, 104.4, 56.4; HRMS (ESI): *m/z* calculated for $C_{12}H_{11}NOI$ ([M-OTf$^-$])$^+$: 311.9880, found: 311.9891.

Mesityl(3-pyridinium)iodonium bistriflate 3c'

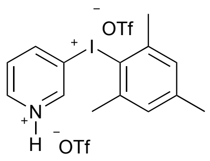

Synthesized according to Method A from **1b** and mesitylene in 70% yield as a beige solid; mp 153-155 ^{o}C; ^{1}H NMR (400 MHz, 10.2 mg in MeOH-d_4 (0.5 mL)) δ 9.12 (d, *J* = 1.9, 1H), 8.88, (dd, *J* = 5.0, 1.1, 1H), 8.55-8.50 (m, 1H), 7.75 (dd, *J* = 8.3, 5, 1H), 7.28 (s, 2H), 2.69 (s, 6H), 2.38 (s, 3H); ^{13}C NMR (100 MHz, 20.0 mg in MeOH-d_4 (0.5 mL)) δ 150.6, 150.0, 147.1, 146.6, 143.8, 131.7, 129.7, 122.4, 121.7 (q, $J_{C\text{-}F}$ = 317), 113.4, 27.1, 21.1; HRMS (ESI): *m/z* calculated for $C_{14}H_{15}NI$ ([M-HOTf-OTf$^-$])$^+$: 324.0244, found: 324.0252.

Mesityl(3-pyridyl)iodonium triflate 3c

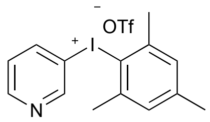

Isolated from **3c'** *via* deprotonation as a white solid in 92% yield; mp 173-175 ^{o}C; ^{1}H NMR (400 MHz, MeOH-d_4) δ 8.99 (d, *J* = 2, 1H), 8.79 (dd, *J* = 4.4, 1.2, 1H), 8.34 (ddd, *J* = 8.4, 2.4, 1.2, 1H), 7.54 (ddd, *J* = 8.4, 4.8, 0.8, 1H), 7.26 (s, 2H), 2.68 (s, 6H), 2.37 (s, 3H); ^{13}C NMR (100 MHz, MeOH-d_4) δ 153.7, 153.3, 146.2, 143.5, 143.1, 131.5, 128.6, 122.3, 121.8 (q, $J_{C\text{-}F}$ = 316), 113.8, 27.1, 21.0; HRMS (ESI): *m/z* calculated for $C_{14}H_{15}NI$ ([M-OTf$^-$])$^+$: 324.0244, found: 324.0231.

2,4,6-Triisopropylphenyl(3-pyridinium)iodonium bistriflate 3d'

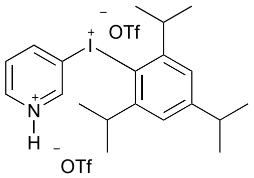

Synthesized according to Method A from **1a** and 1,3,5-triisopropylbenzene in 59% yield as a white solid; mp 149-150 ^{o}C; ^{1}H NMR (400 MHz, 5.0 mg in MeOH-d_4 (0.5 mL)) δ 9.0 (d, *J* = 0.4, 1H), 8.82 (dd, *J* = 4.8, 1.3, 1H), 8.34 (ddd, *J* = 8.3, 2.3, 1.3, 1H), 7.64 (ddd, *J* = 8.4, 4.8, 0.8, 1H), 7.37 (s, 2H), 3.46-3.40 (m, 2H), 3.06-2.99 (m, 1H), 1.31 (d, *J* = 6.8, 12H), 1.27, (d, *J* = 7.0, 6H); ^{13}C NMR (100 MHz, 20.0 mg in MeOH-d_4 (0.5 mL)) δ 157.6, 153.6, 151.0, 150.6, 145.9, 129.5, 126.8, 123.3, 121.8 (q, $J_{C\text{-}F}$ = 316), 114.1, 40.8, 35.4, 24.5, 24.0; HRMS (ESI): *m/z* calculated for $C_{20}H_{17}NI$ ([M-HOTf-OTf$^-$])$^+$: 408.1183, found: 408.1192.

4-Fluorophenyl(3-pyridinium)iodonium bistriflate 3e'

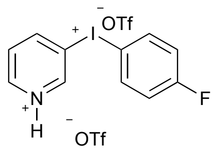

Synthesized according to Method A from **1a** and fluorobenzene in 84% yield as an off-white solid; mp 196-200 ^{o}C; ^{1}H NMR (400 MHz, 10.0 mg in MeOH-d_4 (0.5 mL)) δ 9.37 (d, *J* = 2.0, 1H), 8.93 (dd, 5.1, 1.3, 1H), 8.87-8.83 (m, 1H), 8.36-8.30 (m, 2H), 7.78 (dd, *J* = 8.4, 5.1, 1H), 7.38-7.30 (m, 2H); ^{19}F NMR (376 MHz, 12.8 mg in MeOH-d_4 (0.5 mL)) δ -80.09 (s 6F), -106.18 (s, 1F); ^{13}C NMR (125 MHz, 23.7 mg in MeOH-d_4 (0.5 mL)) δ 166.8 (d, *J* = 254), 151.0, 149.7, 149.0, 140.0 (d, *J* = 10), 129.7, 121.8 (q, $J_{C\text{-}F\ (^{-}OTf)}$ = 317), 120.9 (appd, $J_{C\text{-}F}$ = 23), 115.5, 110.1, (d, $J_{C\text{-}F}$ = 3); HRMS (ESI): *m/z* calculated for $C_{11}H_8NFI$ ([M-HOTf-OTf$^-$])$^+$: 299.9680, found: 299.9684.

4-fluorophenyl(3-pyridyl)iodonium triflate 3e

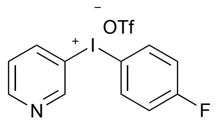

Isolated from **3e'** *via* deprotonation as a white solid in 85% yield; mp 140-141 ^{o}C; ^{1}H NMR (400 MHz, MeOH-d_4) δ 9.24 (d, *J* = 1.9, 1H), 8.84 (dd, *J* = 4.8, 1.8, 1H), 8.63 (ddd, *J* = 8.4, 2.3, 1.4, 1H), 8.35-8.25 (m, 2H), 7.58 (ddd, *J* = 8.3, 4.7, 0.6, 1H), 7.38-7.28 (m, 2H); ^{19}F NMR (376 MHz, MeOH-d_4) δ -80.08 (s, 3F), -106.60 (s, 1F); ^{13}C NMR (100 MHz, MeOH-d_4) δ 166.6 (d, *J* = 253), 154.5, 153.7, 144.0, 139.6 (d, $J_{C\text{-}F}$ = 9), 128.3, 121.8 (q, $J_{C\text{-}F\ (^{-}OTf)}$ = 317), 120.8 (d, $J_{C\text{-}F}$ = 23), 116.1, 109.9 (d, $J_{C\text{-}F}$ = 3); HRMS (ESI): *m/z* calculated for $C_{11}H_8NFI$ ([M-OTf$^-$])$^+$: 299.9680, found: 299.9692.

Mesityl(6-chloro-3-pyridyl)iodonium triflate 3f

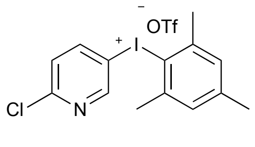

Synthesized according to Method A from **1b** and mesitylene in 73% yield as a brown solid; mp 161-163 ^{o}C; ^{1}H NMR (400 MHz, MeOH-d_4) δ 8.82 (dd, *J* = 2.5, 0.5, 1H), 8.28 (dd, *J* = 8.6, 2.5, 1H), 7.58 (dd, *J* = 8.5, 0.6, 1H), 7.26 (s, 2H), 2.68 (s, 6H), 2.37 (s, 3H); ^{13}C NMR (125 MHz, MeOH-d_4) δ 155.9, 154.3, 146.3, 145.5, 143.5, 131.5, 129.2, 122.4, 121.8 (q, $J_{C\text{-}F}$ = 317), 111.4, 27.1, 21.0; HRMS (ESI): *m/z* calculated for $C_{14}H_{14}ClNI$ ([M-OTf$^-$])$^+$: 357.9854, found: 357.9843.

4-Methoxyphenyl(3-quinolinium)iodonium bistriflate 3g'

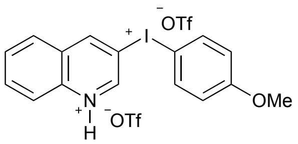

Synthesized according Method B from **1c**[2] and anisole (**2b**) in 90% yield as a white solid; mp 193-196 ^{o}C; ^{1}H NMR (400 MHz, 7.2 mg in MeOH-d_4 (0.5 mL)) δ 9.51 (appd, *J* = 2.0, 1H), 9.47 (appd, *J* = 2.0, 2H), 8.22 (appd, *J* = 9, 2H), 8.19-8.14 (m, 2H), 8.09-8.04 (m, 1H), 7.92-7.85 (m, 1H), 7.09 (appd, *J* = 9.2, 2H), 3.84 (s, 3H); ^{13}C NMR (100 MHz, MeOH-d_4) δ 164.9, 150.8, 150.3, 139.1, 136.8, 131.5, 130.9, 130.6, 125.9, 121.8, (q, $J_{C\text{-}F}$ = 316), 119.2, 109.6, 104.9, 101.4, 56.4; HRMS (ESI): *m/z* calculated for $C_{16}H_{13}NOI$ ([M-HOTf-OTf$^-$])$^+$: 362.0036, found: 362.0041.

4-Methoxyphenyl(3-quinolinyl)iodonium triflate 3g

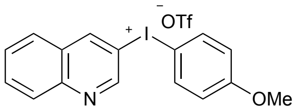

Isolated from **13g'** *via* deprotonation in 87% yield as a white solid; mp 143-144 ^{o}C; ^{1}H NMR (400 MHz, MeOH-d_4) δ 9.35 (d, *J* = 2.4, 1H), 9.27 (d, *J* = 2, 2H), 8.20 (appd, *J* = 9.2, 1H), 8.11 (d, *J* =8.8, 1H), 8.04 (d, *J* = 8.4, 1H), 7.98-7.92 (m, 1H), 7.81-7.75 (m, 1H), 7.08 (appd, *J* = 9.2, 2H), 3.84 (s, 3H); ^{13}C NMR (100 MHz, MeOH-d_4) δ 164.7, 152.5, 149.1, 145.2, 138.7, 134.2, 130.6, 130.1, 130.0, 129.8, 121.8 (q, $J_{C\text{-}F}$ = 317), 119.1, 111.5, 104.7, 56.4; HRMS (ESI): *m/z* calculated for $C_{16}H_{13}NOI$ ([M-OTf$^-$])$^+$: 362.0036, found: 362.0031.

Mesityl(3-quinolinium)iodonium bistriflate 3h'

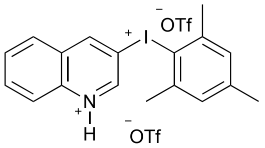

Synthesized according to Method A from **1c**[2] and mesitylene in 77% yield as a light brown solid; mp 155-156 ^{o}C; ^{1}H NMR (400 MHz, 5.7 mg in MeOH-d_4 (0.5 mL)) δ 9.22 (d, *J* = 2.0, 1H), 9.15 (d, *J* = 2.0, 1H), 8.15 (d, *J* = 8.8, 1H), 8.09 (d, *J* = 8.4, 1H), 8.04-7.99 (m, 1H), 7.85-7.79 (m, 1H), 7.27 (s, 2H), 2.74 (s, 6H), 2.36 (s, 3H); ^{13}C NMR (100 MHz, MeOH-d_4) δ 150.2, 149.5, 146.5, 144.2, 143.8, 136.7, 131.6, 131.4, 131.2, 130.5, 125.8, 122.7, 121.7 (q, $J_{C\text{-}F}$ = 316), 107.3, 27.2, 21.0; HRMS (ESI): *m/z* calculated for $C_{18}H_{17}NI$ ([M-HOTf-OTf$^-$])$^+$: 374.0400, found: 374.0399.

2,4,6-Triisopropylphenyl(3-quinolinium)iodonium bistriflate 3i'

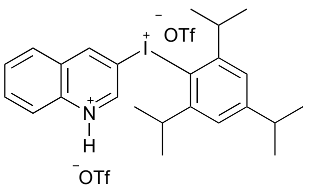

Synthesized according to Method A from **1c**[2] and 1,3,5-triisopropylbenzene in 82% as an off-white solid; mp 135-138 ^{o}C; ^{1}H NMR (400 MHz, 10.4 mg in MeOH-d_4 (0.5 mL)) δ 9.18 (s, 1H), 9.16 (s, 1H), 8.15 (appd, *J* = 8.4, 1H), 8.10 (appd, *J* = 8, 1H), 8.06-8.00 (m, 1H), 7.87-7.81 (m, 1H), 7.37 (s, 2H), 3.61-3.51 (m, 2H), 3.06-2.97 (m, 1H), 1.33 (d, *J* = 6.8, 12H), 1.26 (d, *J* = 6.8, 6H); ^{13}C NMR (100 MHz, MeOH-d_4) δ 157.6, 153.7, 150.4, 148.1, 145.5, 136.2, 131.3, 131.0, 130.3, 127.1, 126.8, 123.9, 121.9 (q, $J_{C\text{-}F}$ = 316), 108.4, 40.9, 35.5, 24.7, 24.1; HRMS (ESI): *m/z* calculated for $C_{24}H_{29}NI$ ([M-HOTf-OTf^{-}])$^{+}$: 458.1339, found: 458.1314.

4-Methoxyphenyl(3-pyrazolium)iodonium bistriflate 3j'

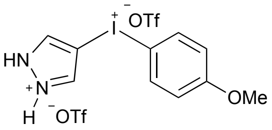

Synthesized according to Method B from **1d**[3] and anisole (**2b**) in 83% yield; mp 183-185 ^{o}C; ^{1}H NMR (400 MHz, 12.3 mg in MeOH-d_4 (0.5 mL)) δ 8.29 (s, 2H), 8.03 (appd, *J* = 9.2, 2H), 7.03 (d, *J* = 9.2, 2H), 3.84 (s, 3H); ^{13}C NMR (100 MHz, MeOH-d_4) δ 164.3, 140.5, 137.7, 121.8 (q, $J_{C\text{-}F}$ = 316), 118.6, 106.2, 81.5, 56.3; HRMS (ESI): *m/z* calculated for $C_{10}H_{10}N_2OI$ ([M-HOTf-OTf^{-}])$^{+}$: 300.9832, found: 300.9826.

4-Methoxyphenyl(3-pyrazolyl)iodonium triflate 3j

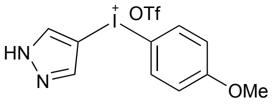

Isolated from **3j'** *via* deprotonation in 78% yield; mp 109-111 ^{o}C; ^{1}H NMR (400 MHz, MeOH-d_4) δ 8.28 (s, 2H), 8.01 (d, *J* = 8.8, 2H), 7.03 (d, *J* = 9.2, 2H), 3.84 (s, 3H); ^{13}C NMR (100 MHz, MeOH-d_4) δ 164.3, 140.6, 137.7, 121.8 (q, $J_{C\text{-}F}$ = 317), 118.6, 106.2, 81.2, 56.3; HRMS (ESI): *m/z* calculated for $C_{10}H_{10}N_2OI$ ([M-OTf^{-}])$^{+}$: 300.9832, found: 300.9843.

Mesityl(3-pyrazolium)iodonium bistriflate 3k'

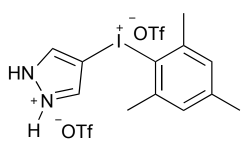

Synthesized according to Method A from **1d**[3] and mesitylene in 75% yield as a brown solid; 169-171 ^{o}C; ^{1}H NMR (400 MHz, 5.8 mg in MeOH-d_4 (0.5 mL)) δ 8.23 (s, 2H), 7.19 (s, 2H), 2.72 (s, 6H), 2.34 (s, 3H); ^{13}C NMR (100 MHz, MeOH-d_4) δ 145.5, 142.6, 140.3, 131.0, 124.2, 121.8 (q, $J_{C\text{-}F}$ = 316), 79.0, 27.0, 21.0; HRMS (ESI): *m/z* calculated for $C_{12}H_{14}N_2I$ ([M-OTf^{-}])$^{+}$: 313.0196, found: 313.0192.

Mesityl(3-pyrazolyl)iodonium triflate 3k

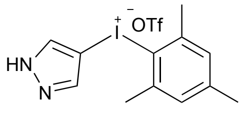

Isolated from **3k'** *via* deprotonation in 72% yield as a brown solid; mp 186-189 ^{o}C; ^{1}H NMR (400 MHz, MeOH-d_4) δ 8.20 (s, 2H), 7.18 (s, 2H), 2.72 (s, 6H), 2.33 (s, 3H); ^{13}C NMR (100 MHz, MeOH-d_4) δ 145.5,

142.6, 140.4, 131.0, 124.2, 121.8 (q, J_{C-F} = 316), 78.7, 27.0, 21.0; HRMS (ESI): *m/z* calculated for $C_{12}H_{14}N_2I$ ([M-OTf$^-$])$^+$: 313.0196, found: 313.0196.

2,4,6-Triisopropylphenyl(3-pyrazolium)iodonium bistriflate 3l'

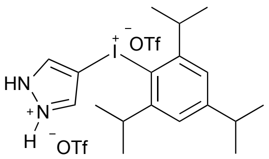

Synthesized according to Method A from **1d**[3] and 1,3,5-triisopropylbenzene in 75% yield as a white solid; mp 175-179 ^oC; ^1H NMR (400 MHz, 4.7 mg in MeOH-d_4 (0.5 mL)) δ 8.16 (s, 2H), 7.28 (s, 2H), 3.57-3.47 (m, 2H), 3.02-2.94 (m, 1H), 1.34 (d, *J* = 6.8, 12H), 1.25 (d, *J* = 6.8, 6H); ^{13}C NMR (100 MHz, MeOH-d_4) δ 156.5, 152.5, 140.1, 126.0, 125.7, 121.8 (q, J_{C-F} = 317), 79.8, 40.4, 35.4, 24.4, 24.0; HRMS (ESI): *m/z* calculated for $C_{18}H_{26}N_2I$ ([M-HOTf-OTf$^-$])$^+$: 397.1135, found: 397.1142.

2,4,6-triisopropylphenyl(3-pyrazolyl)iodonium triflate 3l

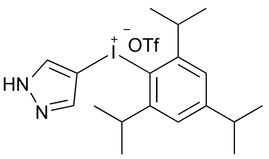

Isolated from **3l'** *via* deprotonation in 90% yield as a white solid; mp 166-168 ^oC; ^1H NMR (400 MHz, MeOH-d_4) δ 8.11 (s, 2H), 7.27 (s, 2H), 3.60-3.48 (m, 2H), 3.04-2.90 (m, 1H), 1.34 (d, *J* = 6.8, 12H), 1.25 (d, *J* = 6.8, 6H); ^{13}C NMR (100 MHz, MeOH-d_4) δ 156.4, 152.5, 140.5, 125.9, 125.8, 121.8 (q, J_{C-F} = 316), 79.0, 40.4, 35.4, 24.4, 24.0; HRMS (ESI): *m/z* calculated for $C_{18}H_{26}N_2I$ ([M-OTf$^-$])$^+$: 397.1135, found: 397.1127.

Mesityl(2,5-dimethyl-3-pyrazolium)iodonium bistriflate 3m'

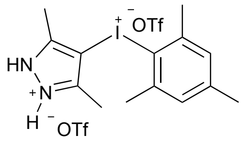

Synthesized according to Method A from **1e** and mesitylene in 67% yield as a slightly brown solid; mp 148-152 ^oC; ^1H NMR (400 MHz, 5.9 mg in MeOH-d_4 (0.5 mL)) δ 7.20 (s, 2H), 2.64 (s, 6H), 2.35 (s, 9H); ^{13}C NMR (100 MHz, 28.2 mg in MeOH-d_4 (0.5 mL)) δ 150.0, 145.4, 142.9, 131.4, 121.3, 121.8 (q, J_{C-F} = 316), 81.7, 26.7, 20.9, 12.2; HRMS (ESI): *m/z* calculated for $C_{14}H_{18}N_2I$ ([M-HOTf-OTf$^-$])$^+$: 341.0509, found: 341.0515.

Mesityl(2,5-dimethyl-3-pyrazolyl)iodonium triflate 3m

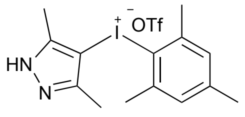

Isolated from **3m'** *via* deprotonation as a light brown solid in 92% yield; mp 178-180 ^oC; ^1H NMR (400 MHz, MeOH-d_4) δ 7.20 (s, 2H), 2.64 (s, 6H), 2.35 (s, 9H); ^{13}C NMR (100 MHz, MeOH-d_4) δ 145.3, 142.9,

131.3, 121.3, 121.8 (q, $J_{C\text{-}F}$ = 317), 80.9, 26.6, 20.9, 12.3; HRMS (ESI): *m/z* calculated for $C_{14}H_{18}N_2I$ ([M-OTf$^-$])$^+$: 341.0509, found: 341.0513.

2,4,6-Triisopropylphenyl(2,5-dimethyl-3-pyrazolium)iodonium bistriflate 3n'

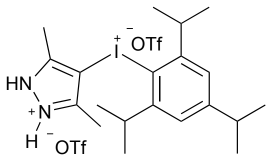

Synthesized according to Method A from **1e** and 1,3,5-triisopropylbenzene in 70% yield as an off-white solid; mp 141-142 ^oC; ^1H NMR (400 MHz, 25.3 mg in MeOH-d_4 (0.5 mL)) δ 7.30 (s, 2H), 3.41-3.34 (m, 2H), 3.03-2.96 (m, 1H), 2.38 (s, 6H), 1.29 (d, *J* = 6.8, 12H), 1.25 (d, *J* = 6.8, 6H); ^{13}C NMR (100 MHz, MeOH-d_4) δ 156.5, 153.0, 149.7, 126.3, 122.9, 121.8 (q, $J_{C\text{-}F}$ = 316), 82.0, 40.5, 35.3, 24.4, 24.0, 12.3; HRMS (ESI): *m/z* calculated for $C_{20}H_{30}N_2I$ ([M-HOTf-OTf$^-$])$^+$: 425.1448, found: 425.1451.

2,4,6-Triisopropylphenyl(2,5-dimethyl-3-pyrazolyl)iodonium triflate 3n

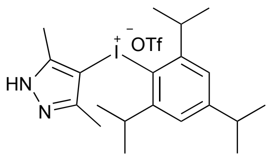

Isolated from **3n'** *via* deprotonation in 97% as a white solid; mp 162-164 ^oC; ^1H NMR (400 MHz, MeOH-d_4) δ 7.30 (s, 2H), 3.42-3.34 (m, 2H), 3.03-2.95 (m, 1H), 2.37 (s, 6H), 1.29 (d, *J* = 6.4, 1H), 1.26 (d, *J* = 7.2, 6H); ^{13}C NMR (100 MHz, MeOH-d_4) δ 156.5, 153.0, 149.6, 126.3, 122.9, 121.8 (q, $J_{C\text{-}F}$ = 316), 81.5, 40.4, 35.3, 24.4, 24.0, 12.4; HRMS (ESI): *m/z* calculated for $C_{20}H_{30}N_2I$ ([M-OTf$^-$])$^+$: 425.1448, found: 425.1438.

5-(1,3-Dimethyluracilyl)(3-pyridinium)iodonium bistriflate 3o'

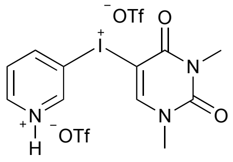

Synthesized according to Method A from **1a** and *N*,*N*-dimethyluracil in 86% yield as a white solid; mp 172-173 ^oC; ^1H NMR (400 MHz, MeOH-d_4) δ 9.37 (s, 1H), 8.97-8.88 (m, 3H), 7.84 (dd, *J* = 8.3, 5.2, 1H), 3.50 (s, 3H), 3.34 (s, 3H); ^{13}C-NMR (100 MHz, MeOH-d_4) δ 160.9, 156.4, 152.3, 150.7, 148.1, 129.2, 121.8 (q, *J* = 317), 115.3, 89.4, 38.5, 29.8; HRMS (ESI): *m/z* calculated for $C_{11}H_{11}N_3O_2I$ ([M-HOTf-OTf$^-$])$^+$: 343.9890, found: 343.9898.

5-(1,3-Dimethyluracilyl)(3-pyridyl)iodonium triflate 13o

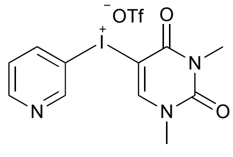

Isolated from **3o'** *via* deprotonation in 81% yield as a white solid; mp 133 °C (decomposed); ^{1}H NMR (400 MHz, MeOH-d_4) δ 9.20 (appd, *J* = 2, 1H), 8.94 (s, 1H), 8.83 (dd, *J* = 4.7, 1.1, 1H), 8.60 (ddd, *J* = 8.3, 2.2, 1.5, 1H), 7.58 (ddd, 8.3, 4.8, 0.4, 1H), 3.49 (s, 3H), 3.34 (s, 3H); ^{13}C NMR (100 MHz, MeOH-d_4) δ 160.9, 156.1, 154.7, 153.6, 152.3, 144.2, 128.2, 121.8 (q, J_{C-F} = 317), 115.8, 89.1, 38.4, 29.8; HRMS (ESI): *m/z* calculated for $C_{11}H_{11}N_3O_2I$ ([M-OTf$^-$])$^+$: 343.9890, found: 343.9902.

4 Arylation of Phenols 4 to Diaryl Ethers 5[4]

Phenylpyridyl ether 5a

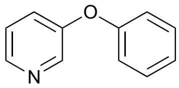

To a suspension of ^{t}BuOK (1.2 equiv, 0.6 mmol, 67 mg) in THF (1.5 mL) was added phenol **4a** (0.5 mmol, 47 mg) at 0 °C and the reaction was left to stir at this temperature for 15 min. Diaryliodonium salt **3b** (1.2 equiv, 0.6 mmol, 278 mg) was added in one portion and the reaction was stirred in an oil bath preheated to 40 °C and until TLC indicated complete consumption of **4a.** The reaction was then quenched with H_2O at 0 °C, the organic phase separated and the water phase extracted with dichloromethane (3 × 10 mL). The combined organic phases were dried (Na_2SO_4), filtered, and concentrated *in vacuo*. The crude material was purified by flash chromatography (pentane:Et_2O, 100:1 → pentane:Et_2O, 25:1) to give diaryl ether **5a** in 88% yield as a transparent oil. Analytical data were in accordance with those previously reported.[5]

Product **5a** was also prepared according to the same protocol using the protonated salt **3b'** and 2.2 equiv of ^{t}BuOK, giving **5a** in 59% yield.

2,4-dimethylphenylpyridyl ether 5b

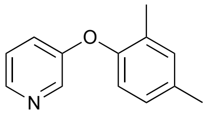

Compound **5b** was synthesized according to the experimental procedure described for product **5a** using 2,4-dimethylphenol **4b**, diaryliodonium salt **13b** and 1.1 equiv ^{t}BuOK. Isolated after column chromatography (pentane:Et_2O, 100:1 → pentane:Et_2O, 25:1) in 65% yield as a transparent oil. Analytical data were in accordance with those previously reported.[4]

Product **5b** was also prepared from the same protocol using the protonated salt **3b'** and 2.2 equiv of ^{t}BuOK, giving **5b** in 52% yield.

5 Arylation of Diethyl Methylmalonate 6 to Product 7[6]

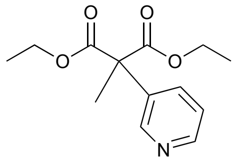

NaH (60% dispersed in mineral oil, 1.3 equiv, 0.65 mmol, 26 mg) was suspended in DMF (1 mL) and diethyl methylmalonate (**6**) (0.5 mmol, 88 mg) was added dropwise at 0 °C. The reaction was allowed to stir at rt for 10 min. A solution of diaryliodonium salt **3c** (1.3 equiv, 0.33 mmol, 308 mg) in DMF (1 mL) was added *via* cannula to the reaction mixture at 0 °C. The reaction mixture was then stirred at rt until TLC indicated complete consumption of **6**. The reaction was quenched with H_2O at 0 °C, extracted with EtOAc (3 × 10 mL) and washed with H_2O (1 × 20 mL) and brine (2 × 20 mL). The combined organic phases were dried ($MgSO_4$) and concentrated *in vacuo*. The crude material was purified with flash chromatography (Pentane:EtOAc, 100:1 → Pentane:EtOAc, 6:1, the EtOAc contained 3% Et_3N) to give **7** in 72% yield as a transparent oil; ^1H NMR (400 MHz, $CDCl_3$) δ 8.63 (d, *J* = 2.4, 1H), 8.54 (d, *J* = 4.8, 1H), 7.72 (appd, *J* = 8.1, 1H), 7.31-7.25 (m, 1H), 4.32-4.16 (m, 4H), 1.89 (s, 3H), 1.26 (t, *J* = 7.1, 6H); ^{13}C NMR (100 MHz, $CDCl_3$) δ 170.9, 149.1, 148.9, 135.5, 134.2, 123.0, 62.2, 57.4, 22.0, 14.1; HRMS (ESI): *m/z* calculated for $C_{13}H_{18}NO_4$ ($[M+H])^+$: 252.1246, found: 252.1236.

Product **7** was also prepared from the same protocol using the protonated salt **3c’** and 2.3 equiv of NaH, giving **7** in 23% yield.

6 References

[1] A. I. Vogel, B. S. Furniss, A. J. Hannaford, V. Rogers, P. W. G. Smith, A. R. Tatchell, *Vogel's Textbook of Practical Organic Chemistry*, **1978**.

[2] A. Klapars, S. L. Buchwald, *J. Am. Chem. Soc.* **2002**, *124*, 14844-14845.

[3] R. E. Sammelson, J. E. Casida, *J. Org. Chem.* **2003**, *68*, 8075-8079.

[4] N. Jalalian, T. B. Petersen, B. Olofsson, *Chem. Eur. J.* **2012**, *18*, 14140-14149.

[5] N. Jalalian, E. E. Ishikawa, L. F. Silva, B. Olofsson, *Org. Lett.* **2011**, *13*, 1552-1555.

[6] C. H. Oh, J. S. Kim, H. H. Jung, *J. Org. Chem.* **1999**, *64*, 1338-1340.

7 Copies of NMR spectra

400 MHz
MeOD

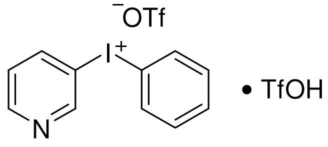

3a'

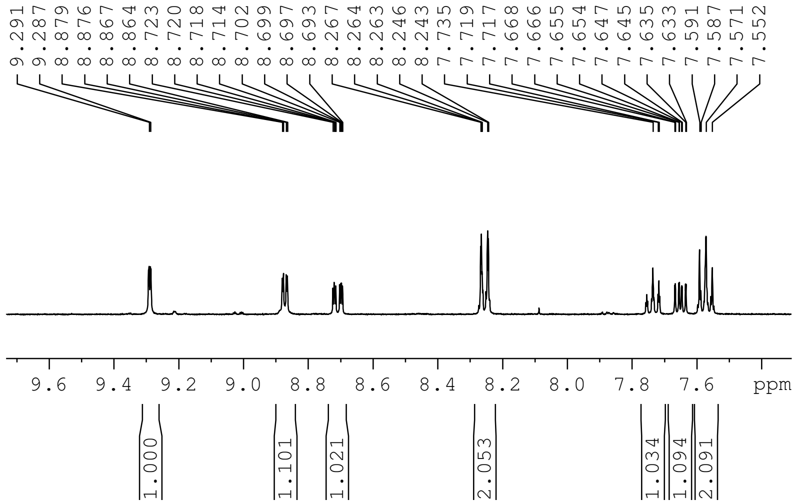

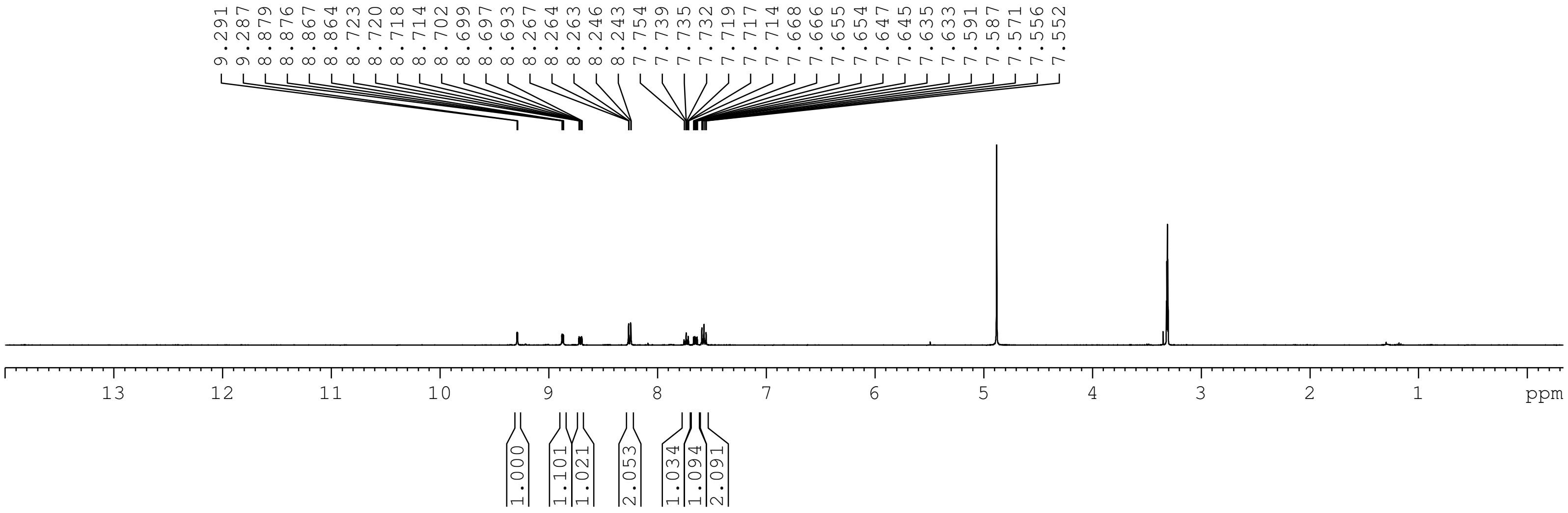

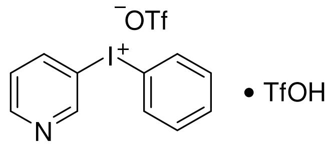

3a'

100 MHz
MeOD

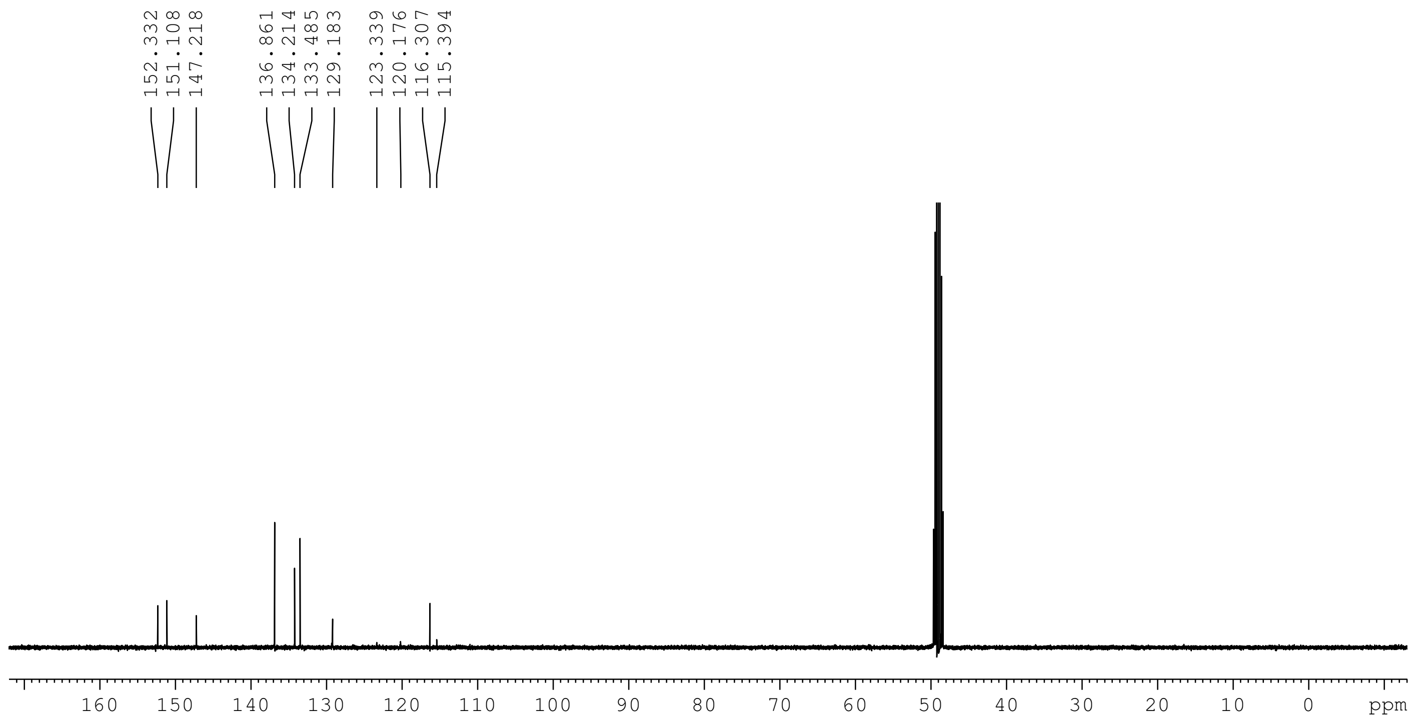

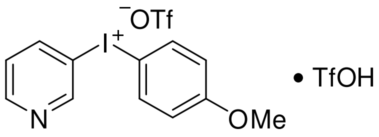

3b'

500 MHz
MeOD

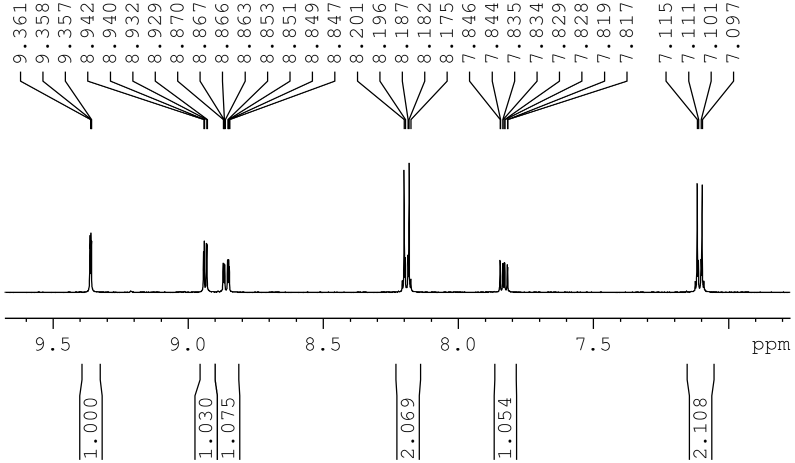

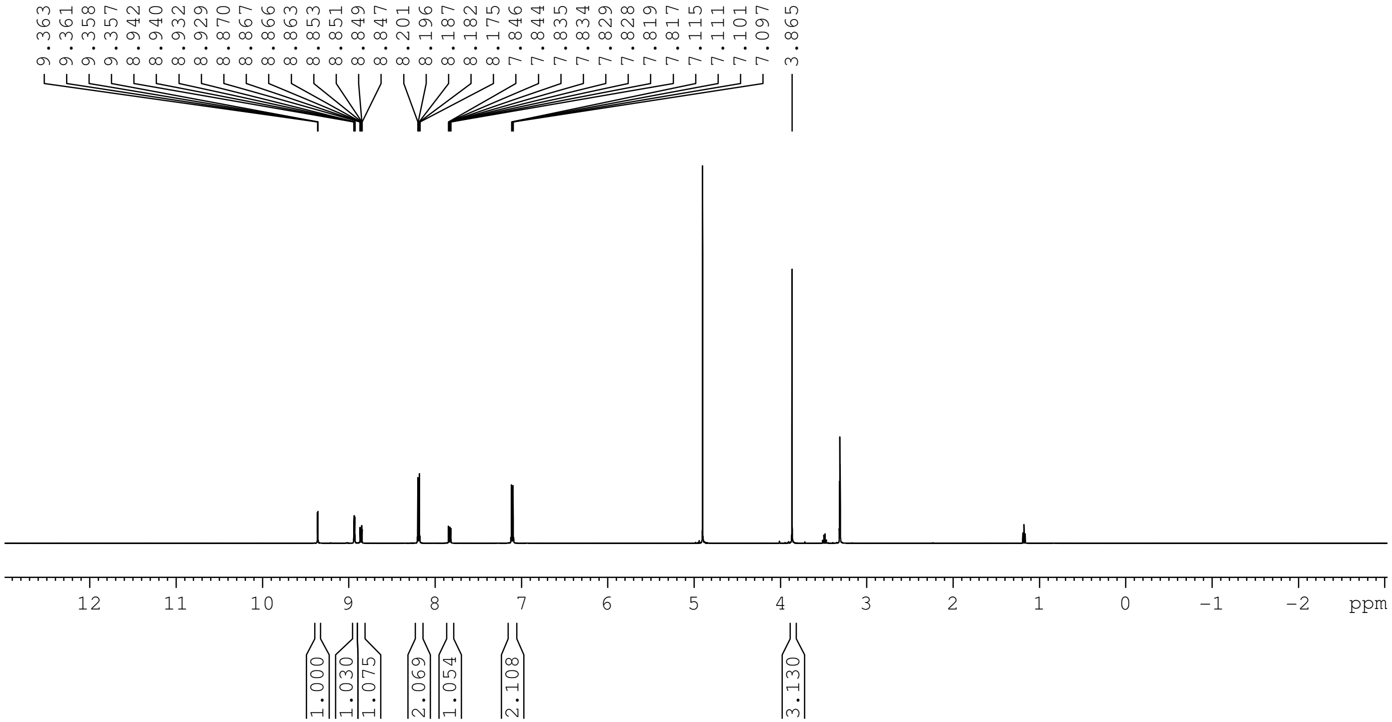

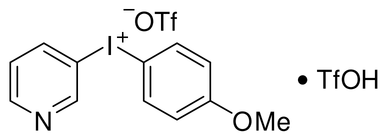

3b'

125 MHz
MeOD

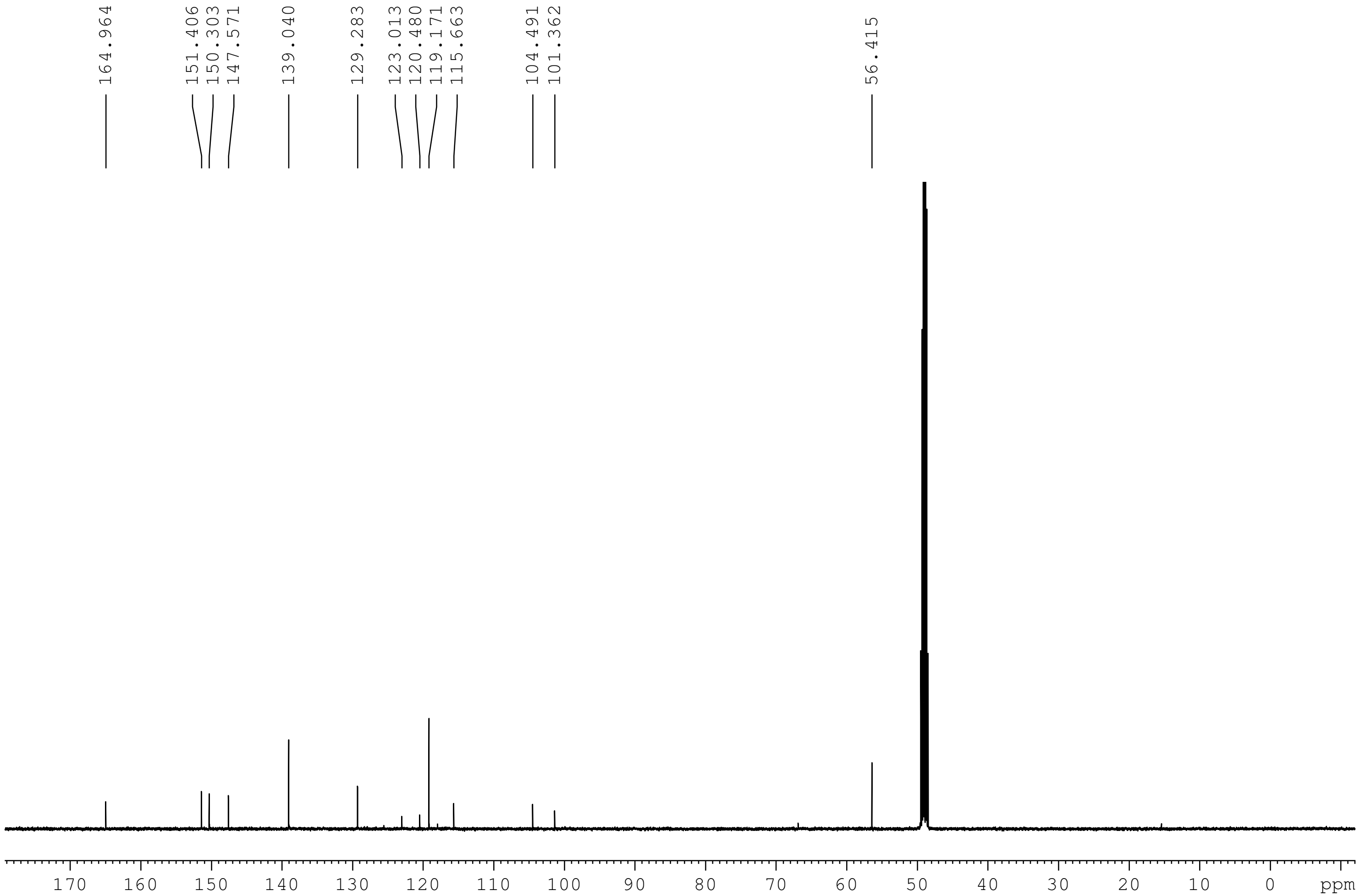

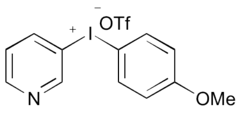

3b

400 MHz
MeOD

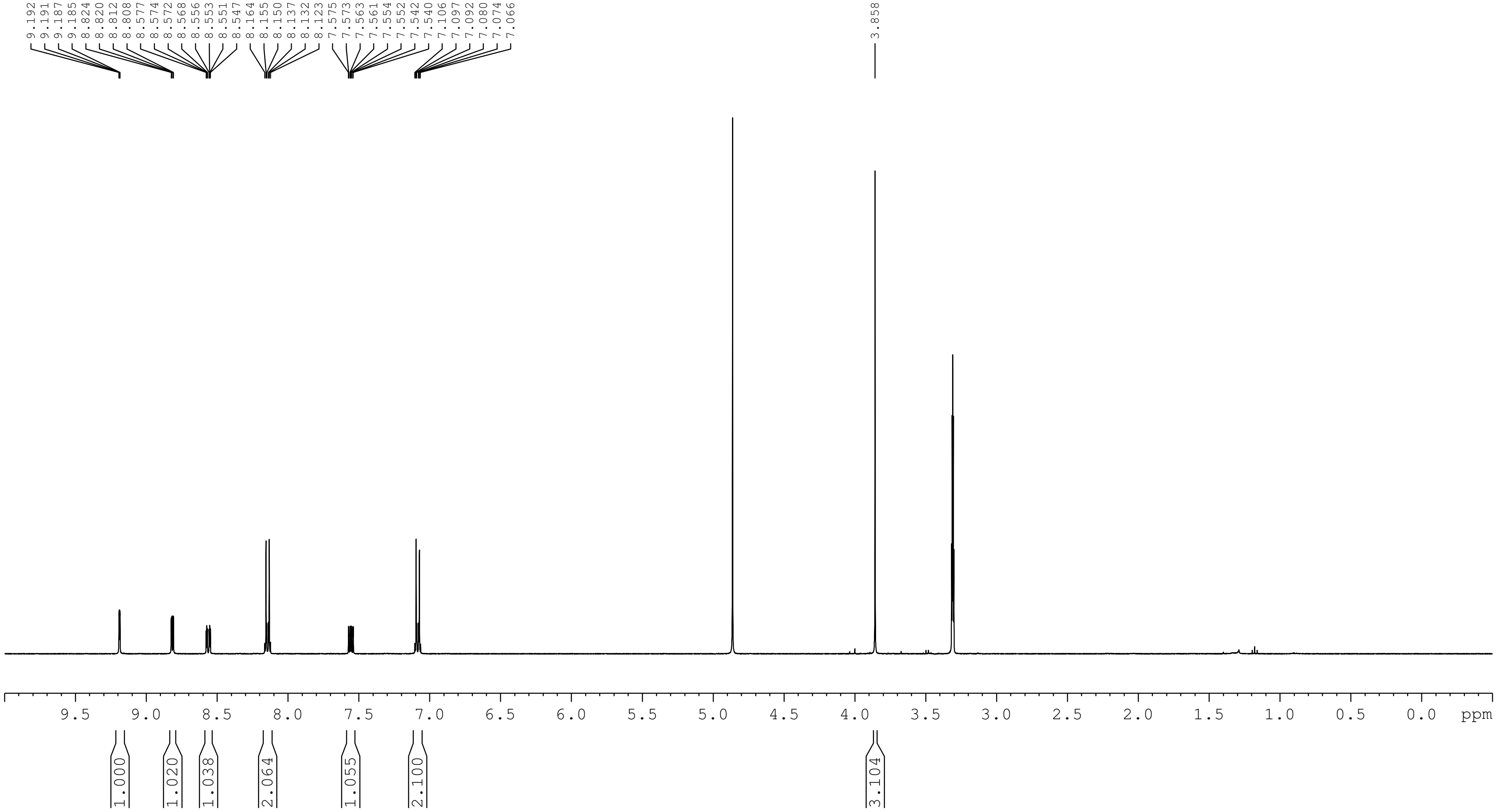

100 MHz
MeOD

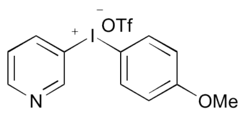

3b

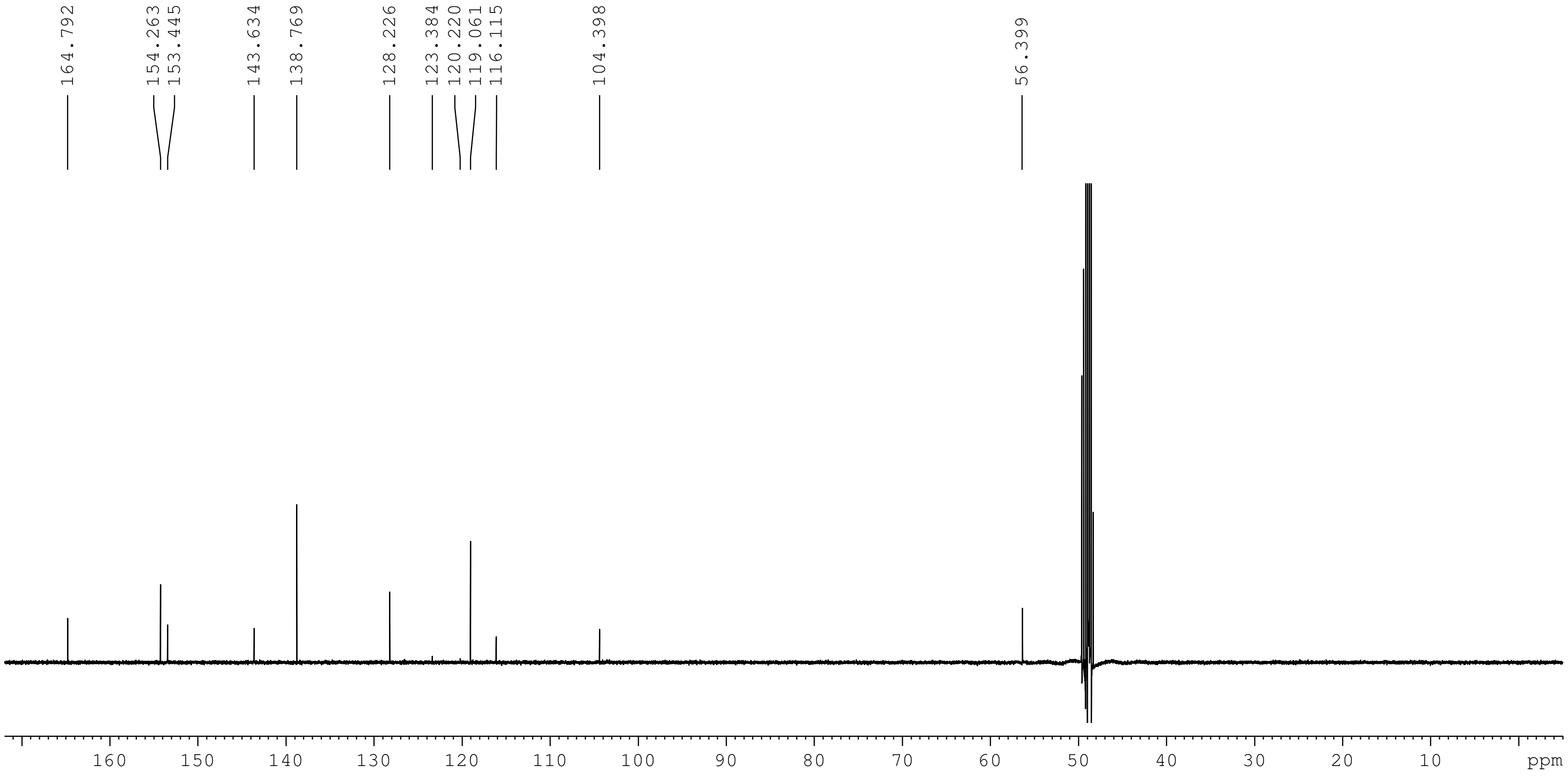

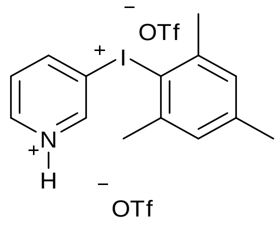

3c'

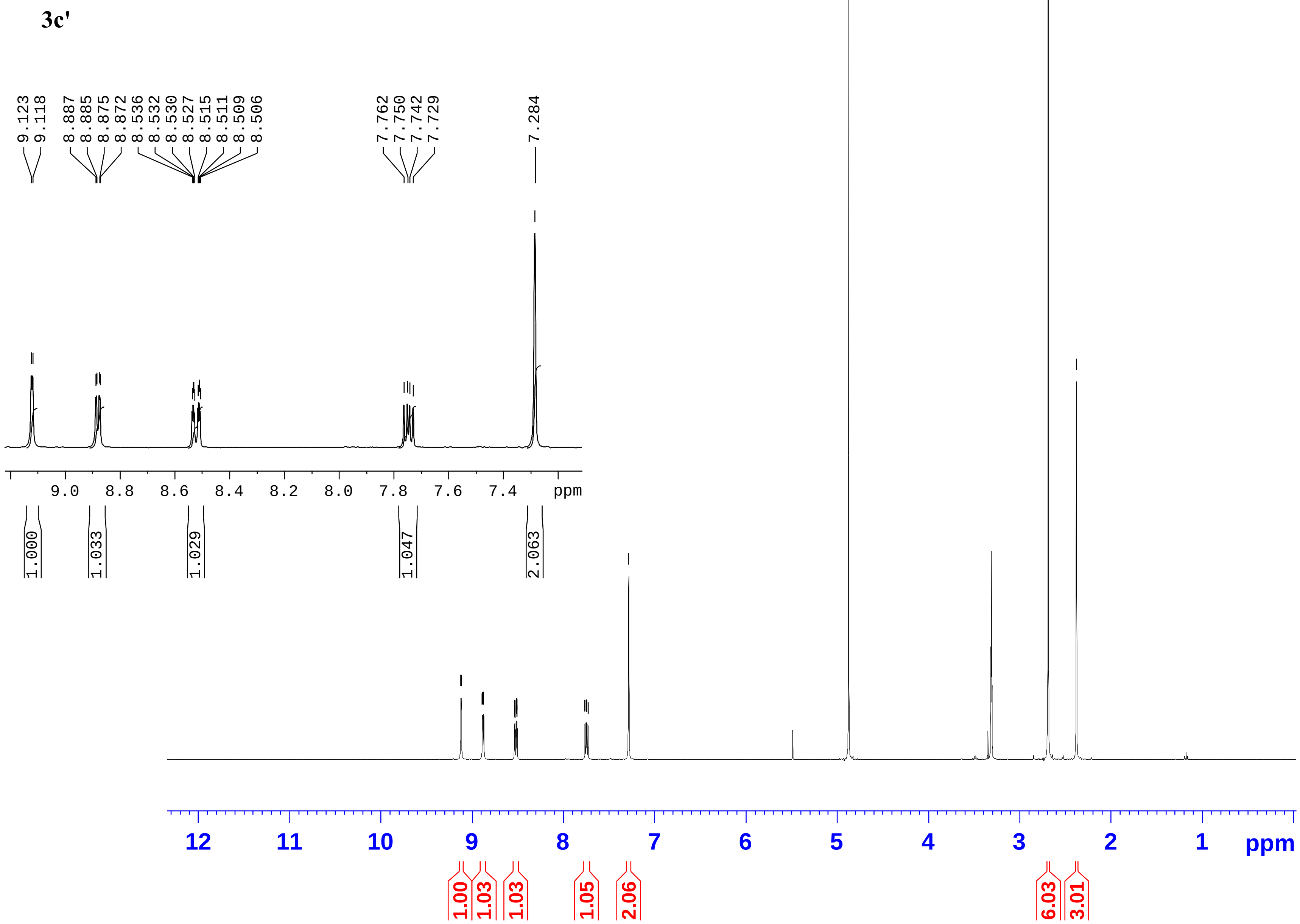

400 MHz
MeOD

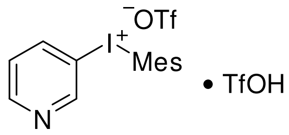

3c'

100 MHz
MeOD

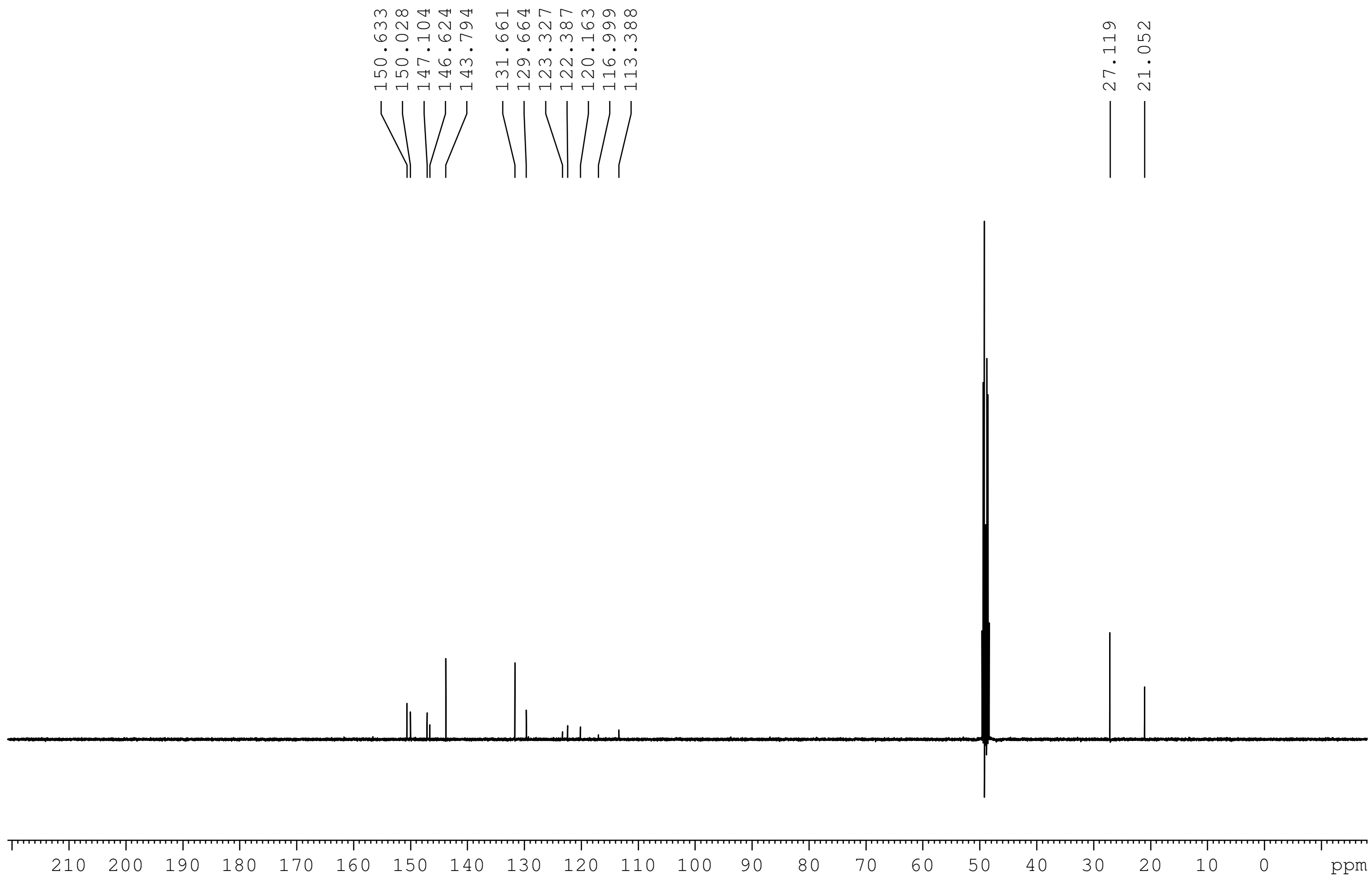

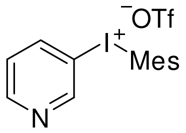

3c

400 MHz
MeOD

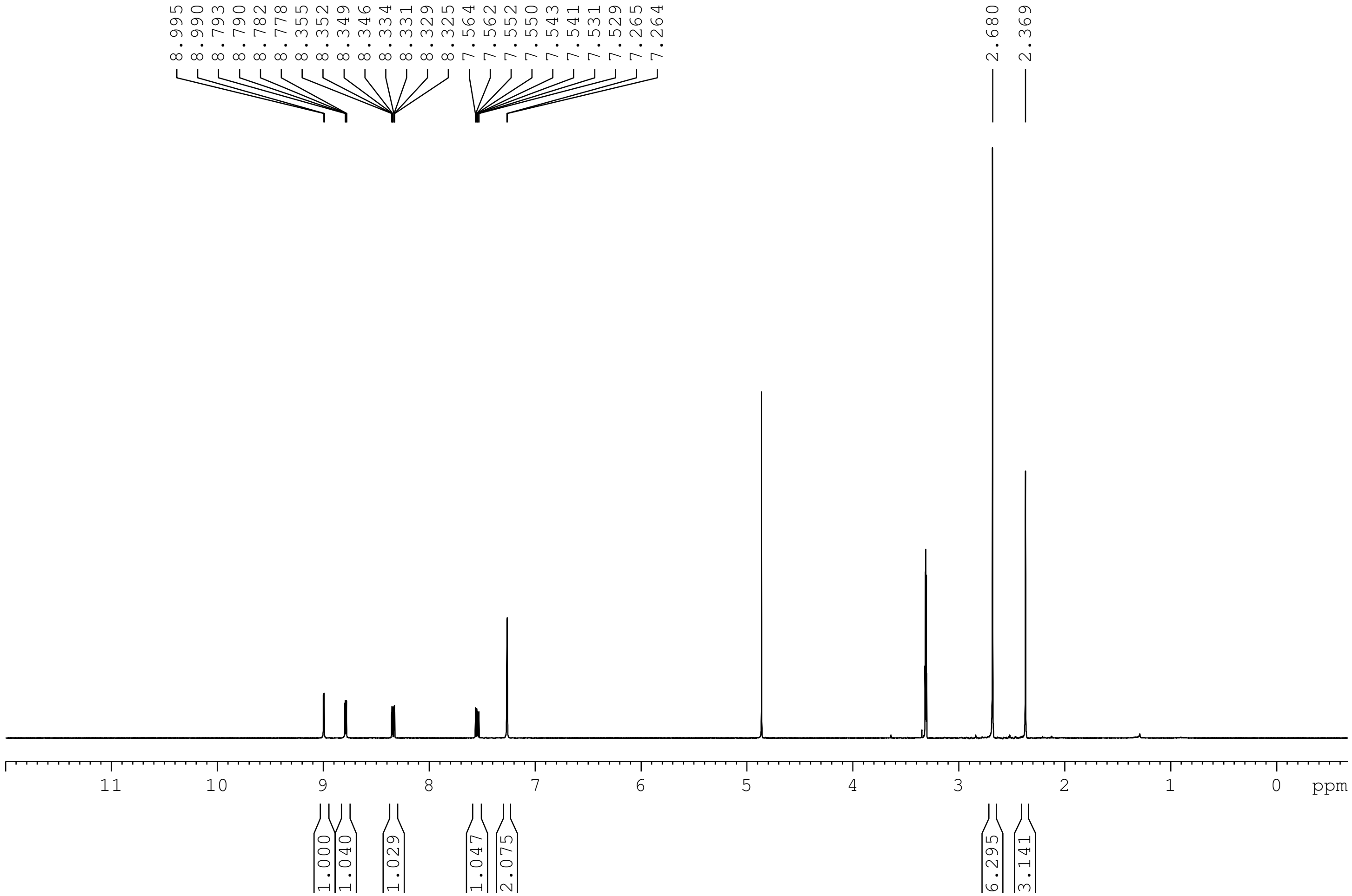

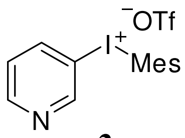

3c

100 MHz
MeOD

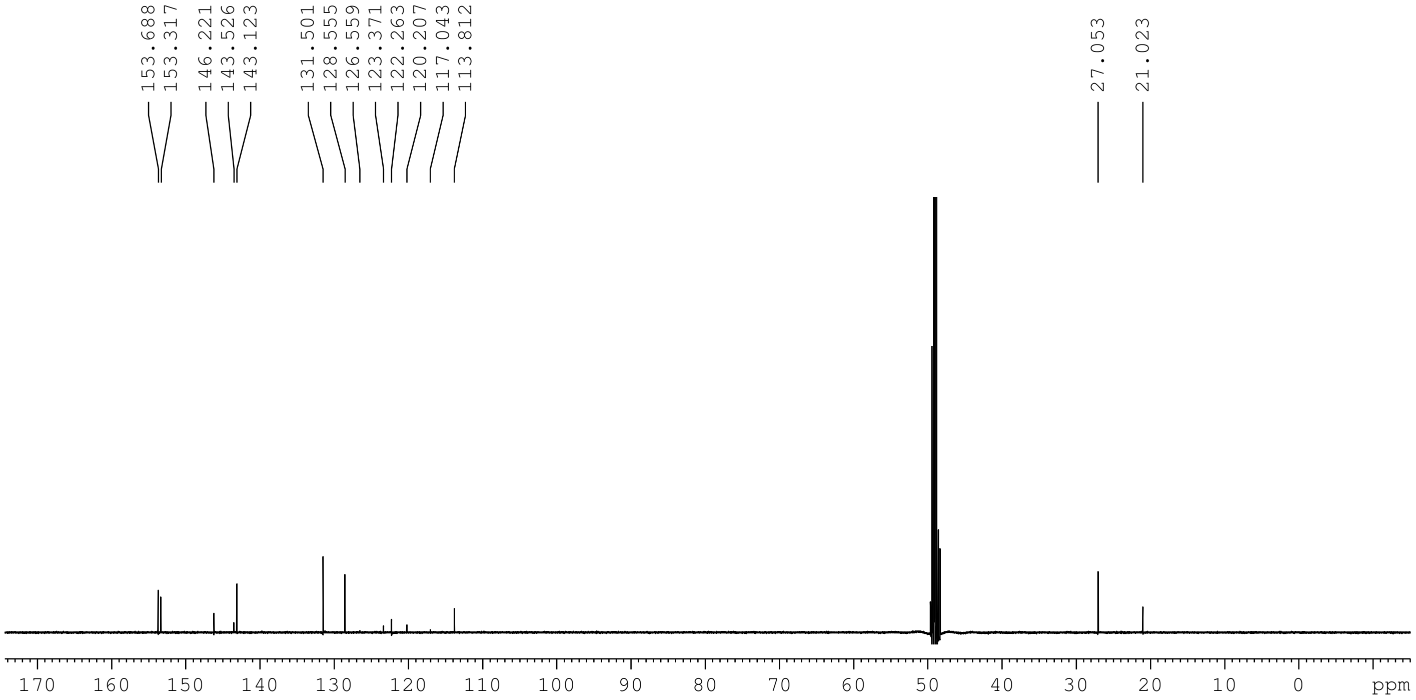

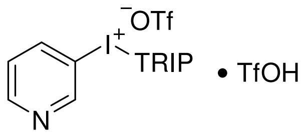

3d'

400 MHz
MeOD

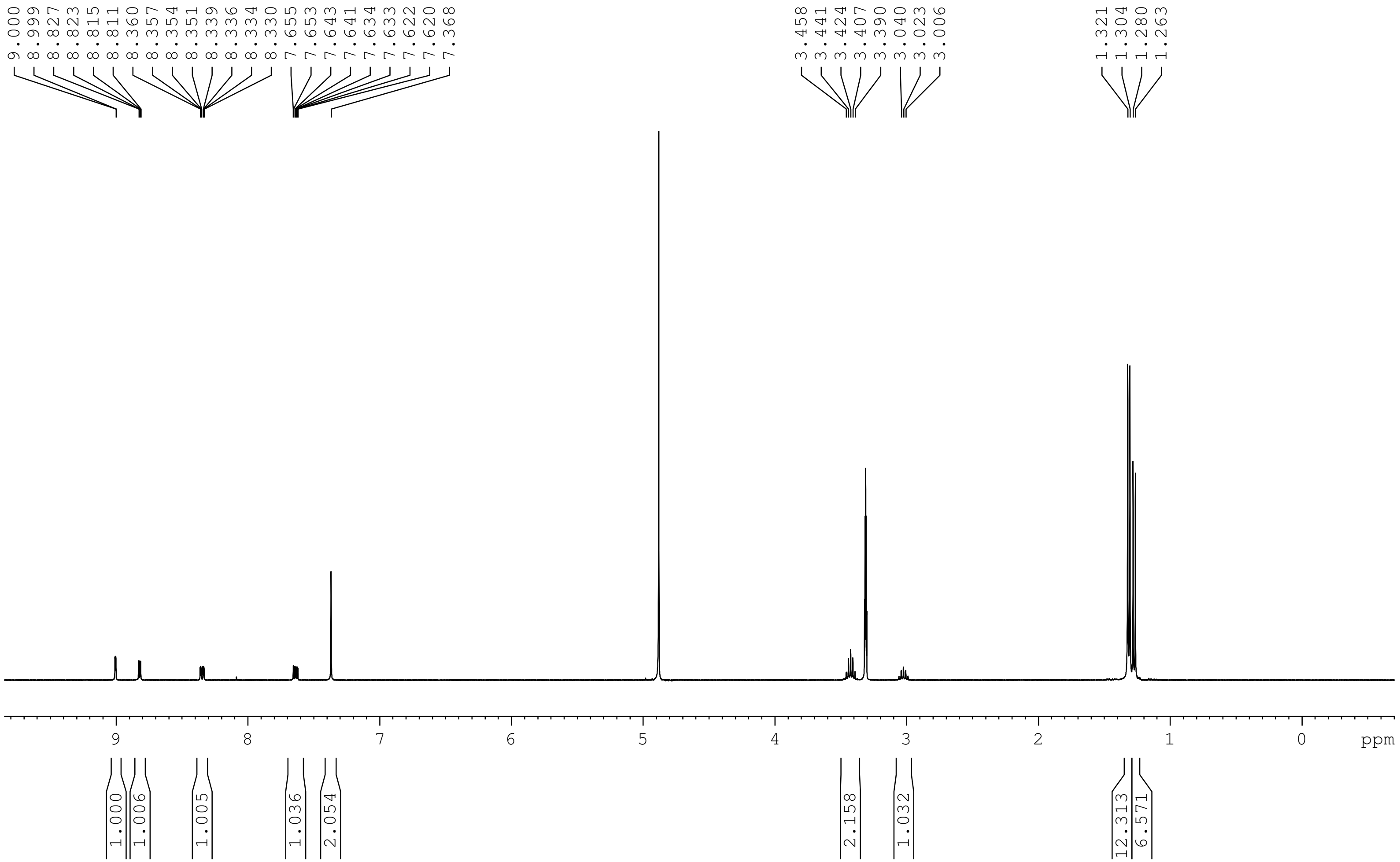

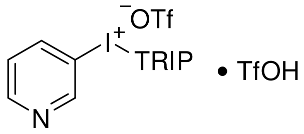

3d'

100 MHz
MeOD

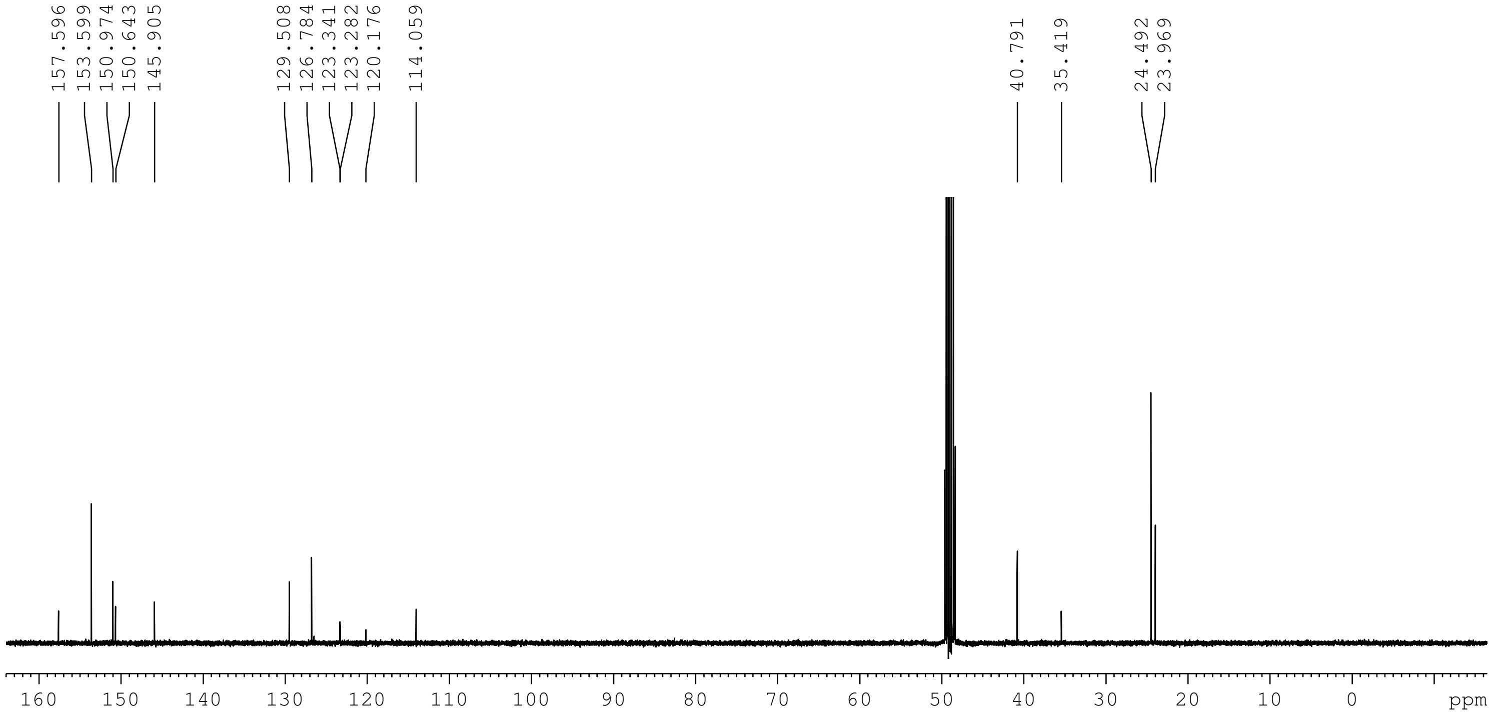

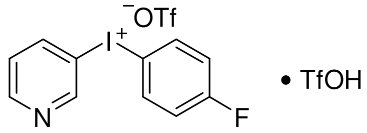

3e'

400 MHz
MeOD

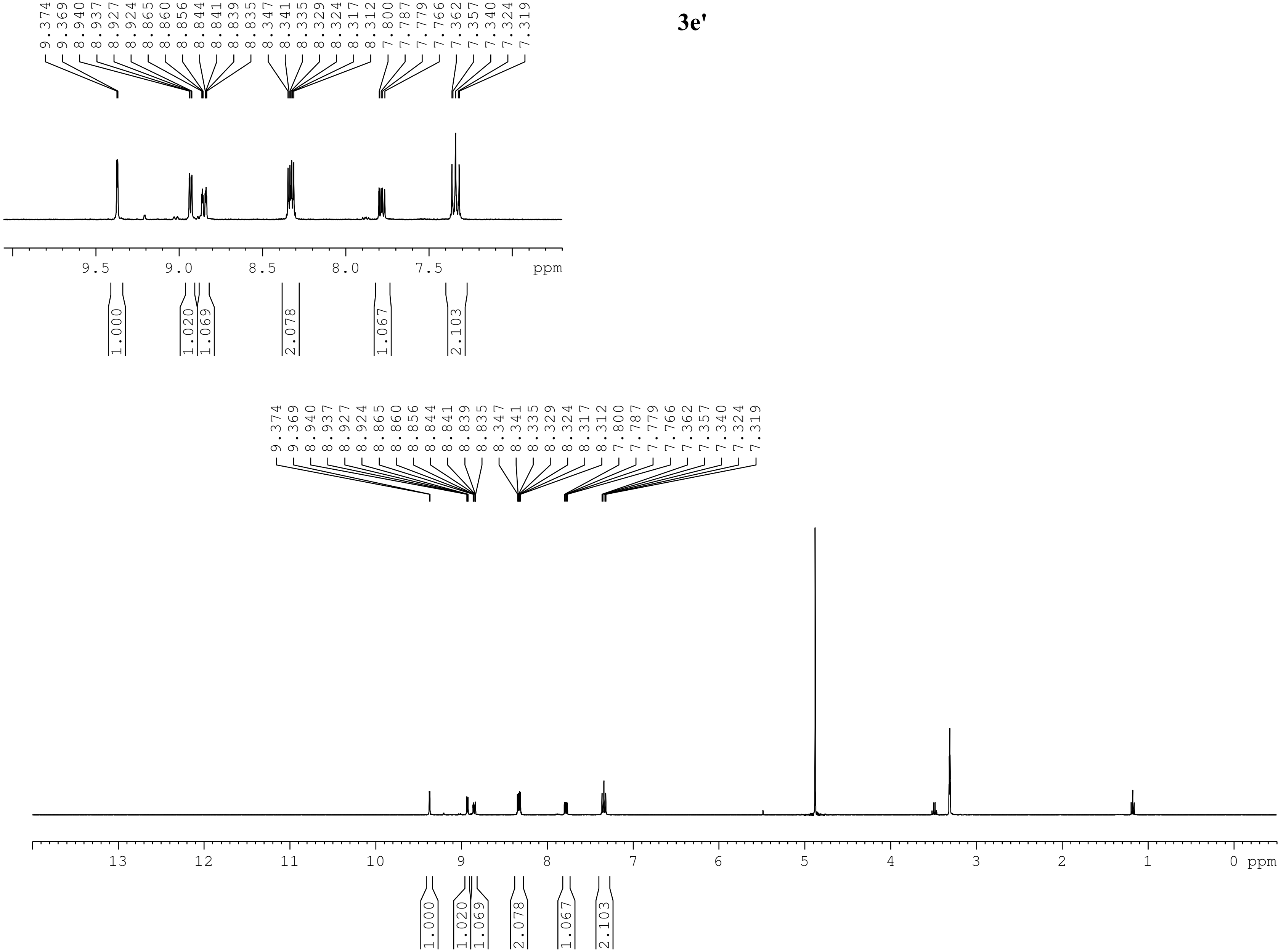

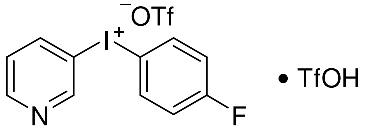

3e'

125 MHz
MeOD

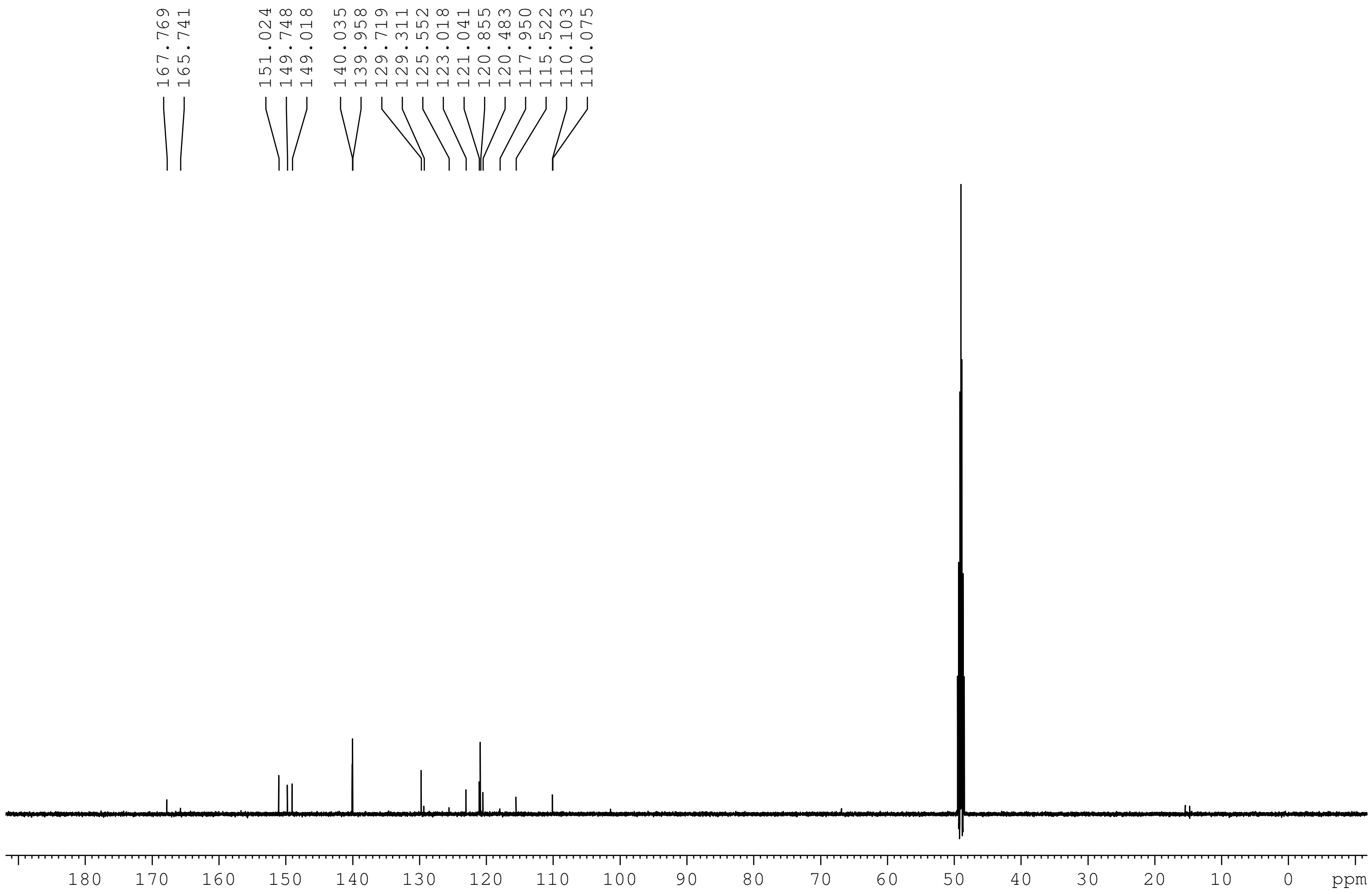

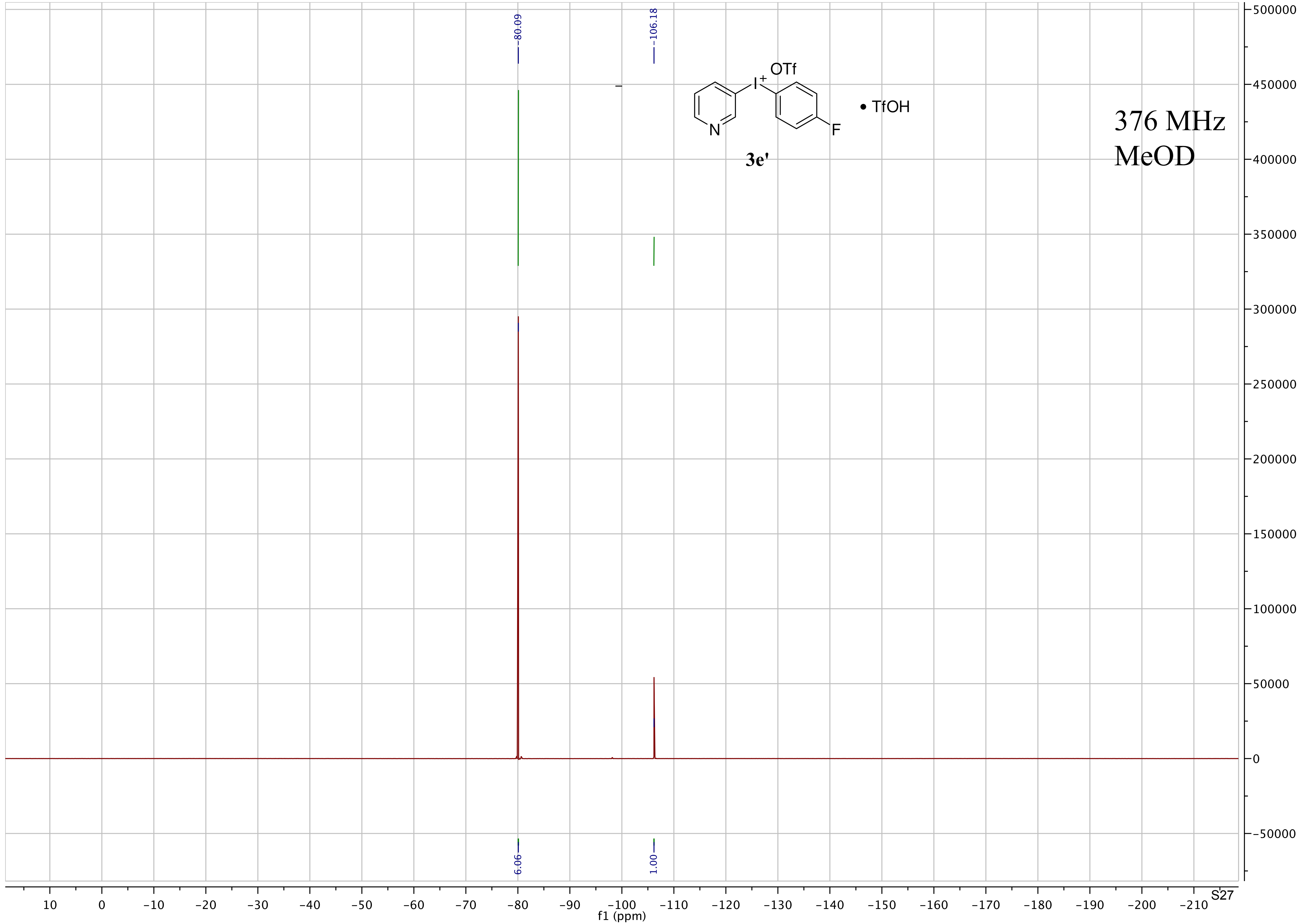
-80.09
-106.18
OTf
I+
N
F
• TfOH
3e'
376 MHz
MeOD
6.06
1.00
f1 (ppm)

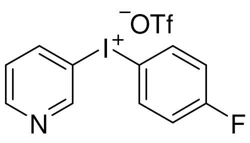

3e

400 MHz
MeOD

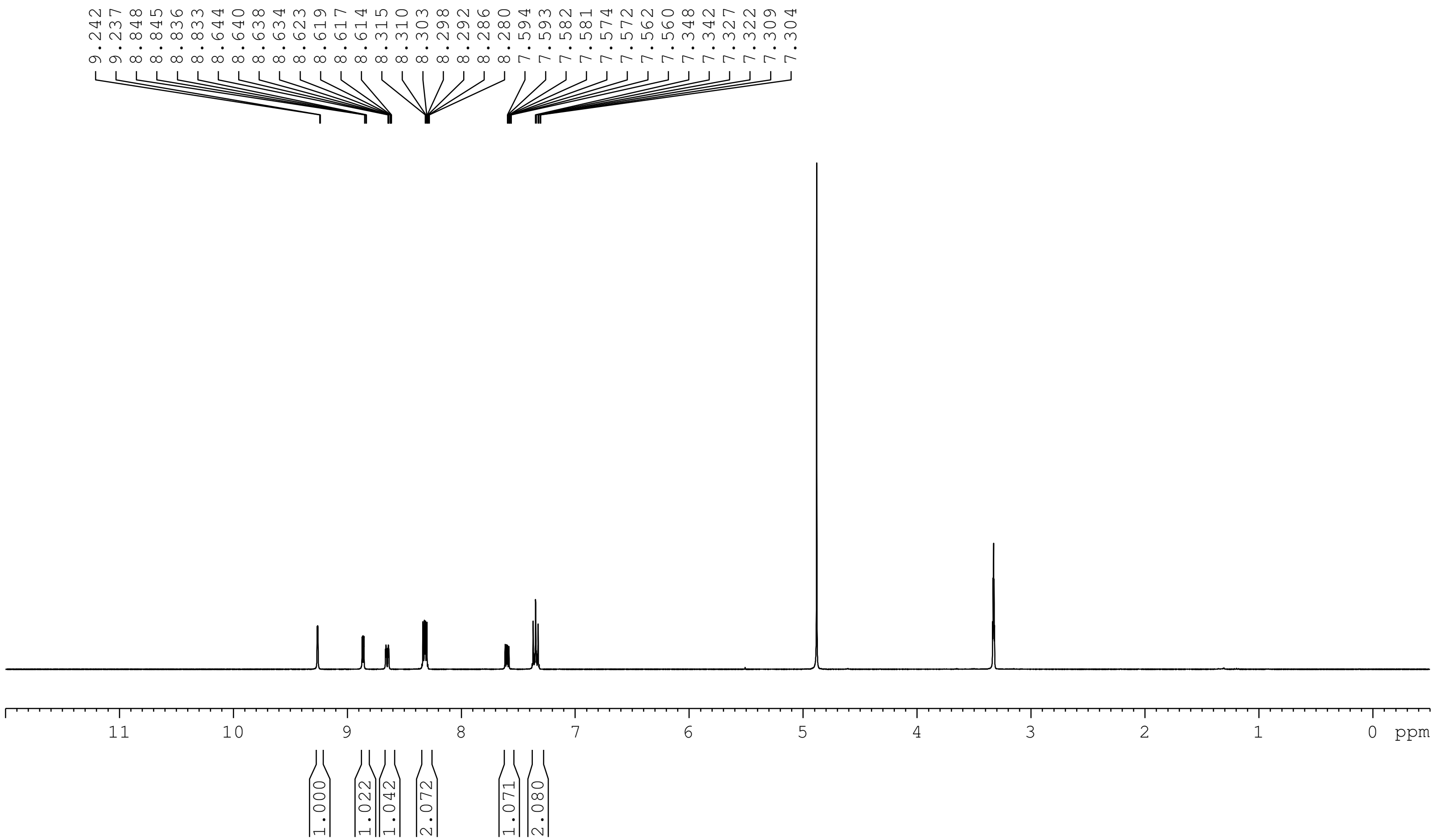

100 MHz
MeOD

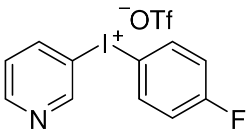

3e

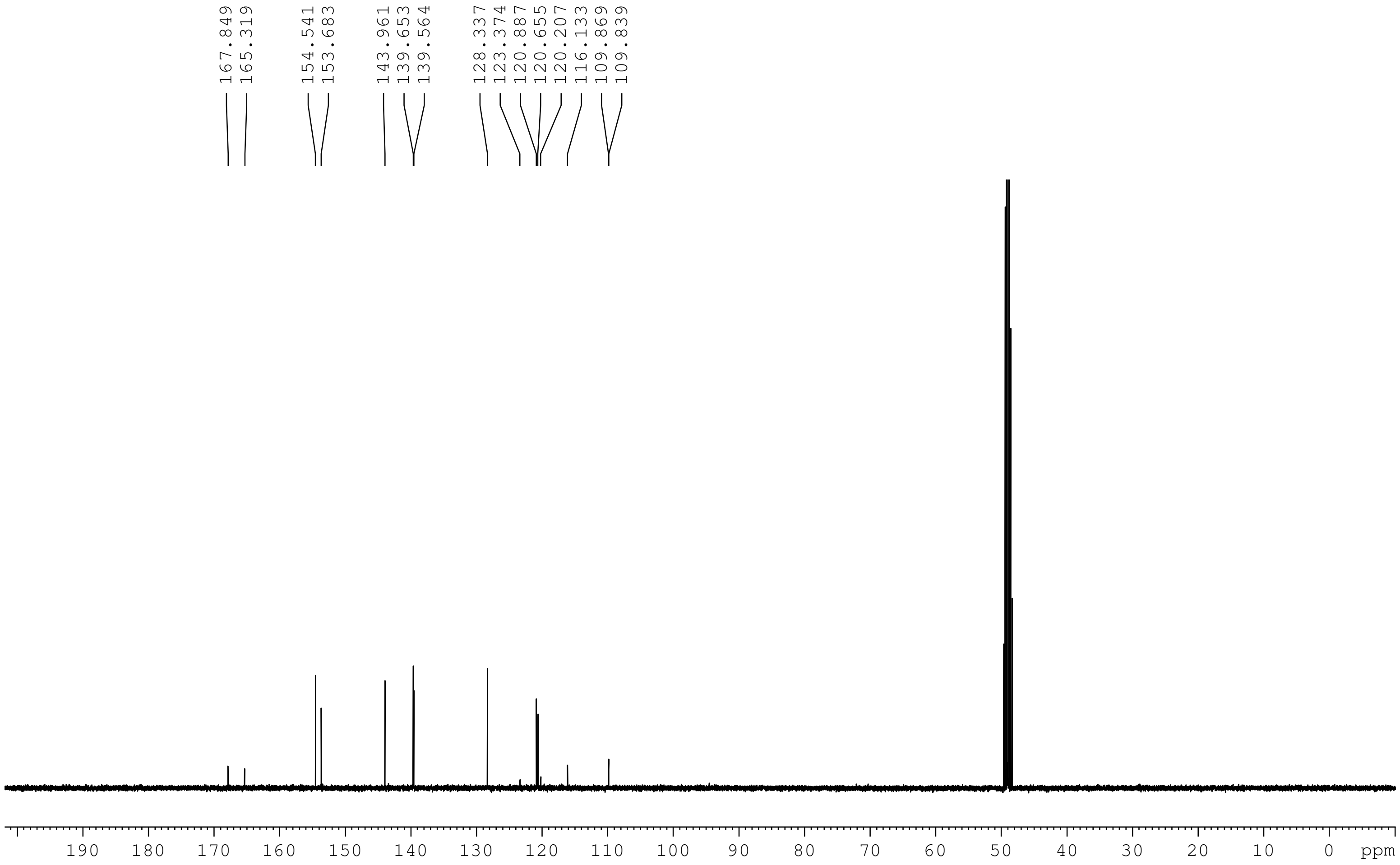

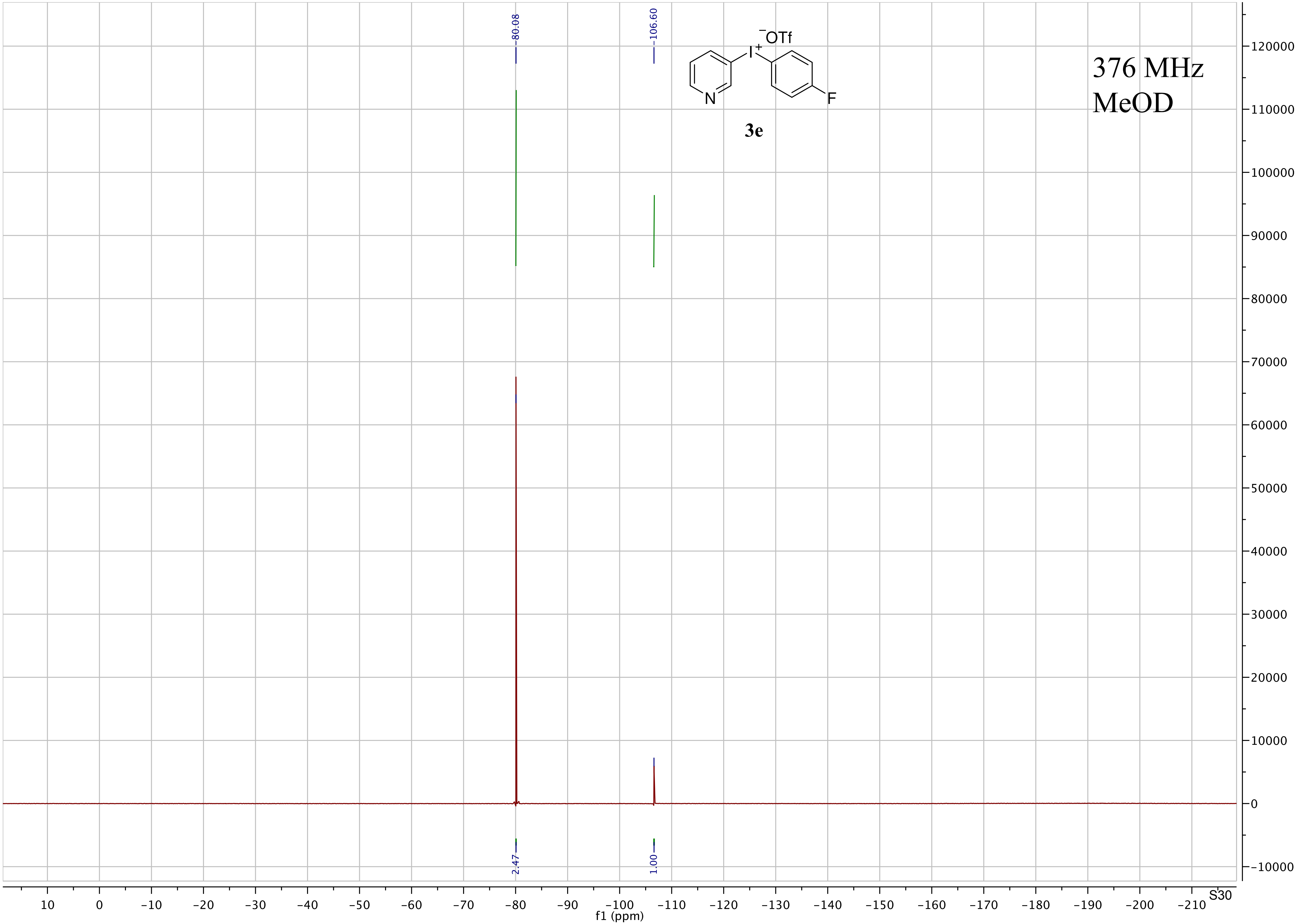

-80.08
-106.60
OTf
I
N
F
3e
376 MHz
MeOD
2.47
1.00
f1 (ppm)

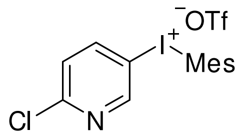

3f

400 MHz
MeOD

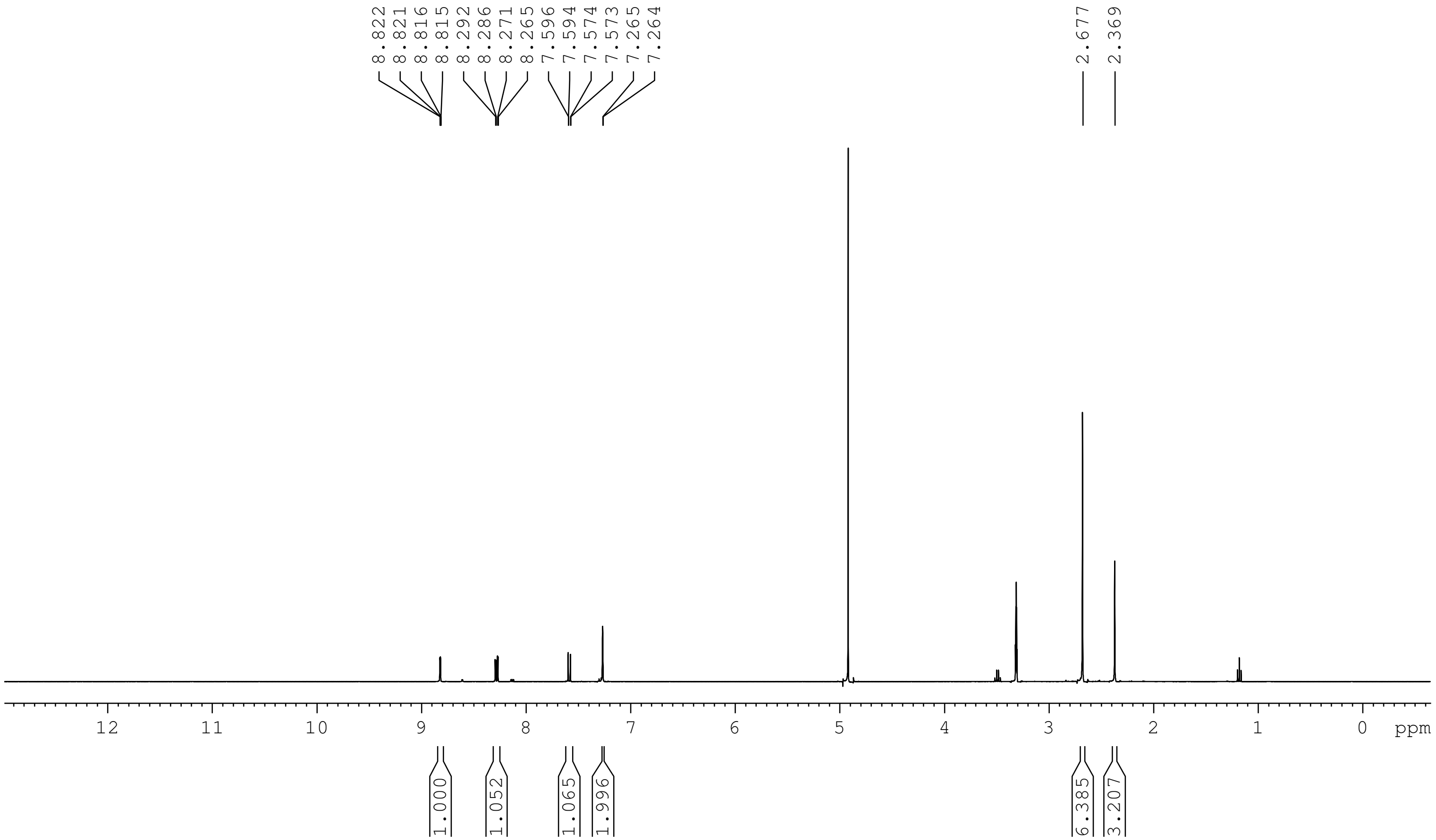

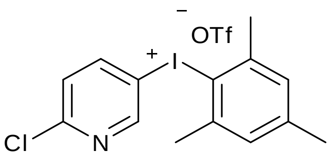

3f

125 MHz
MeOD

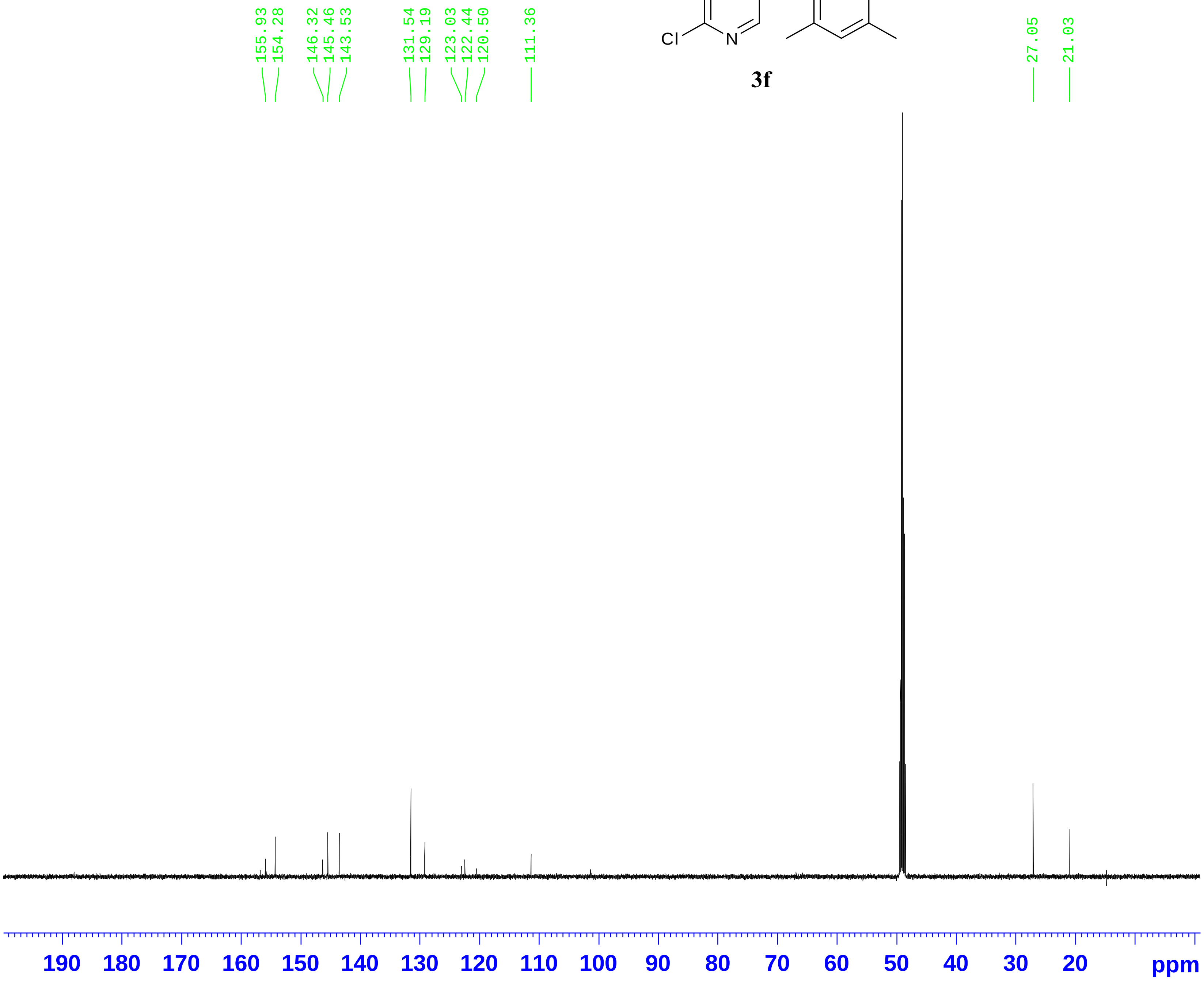

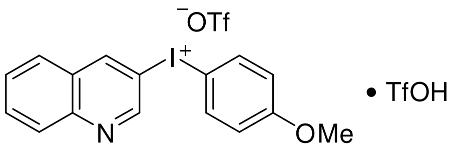

3g'

400 MHz
MeOD

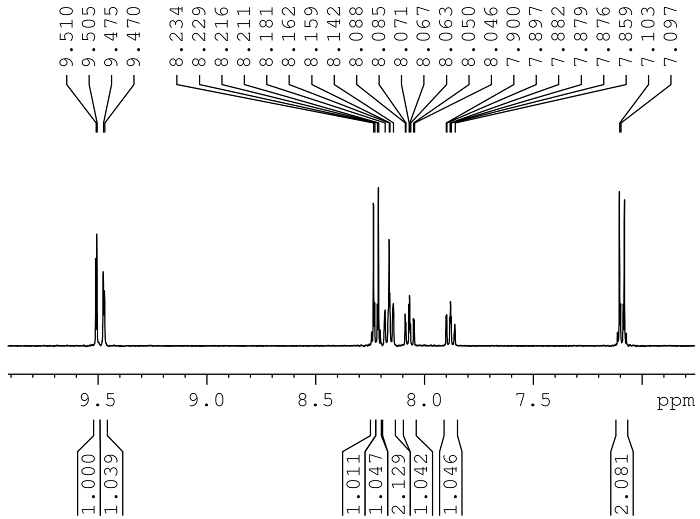

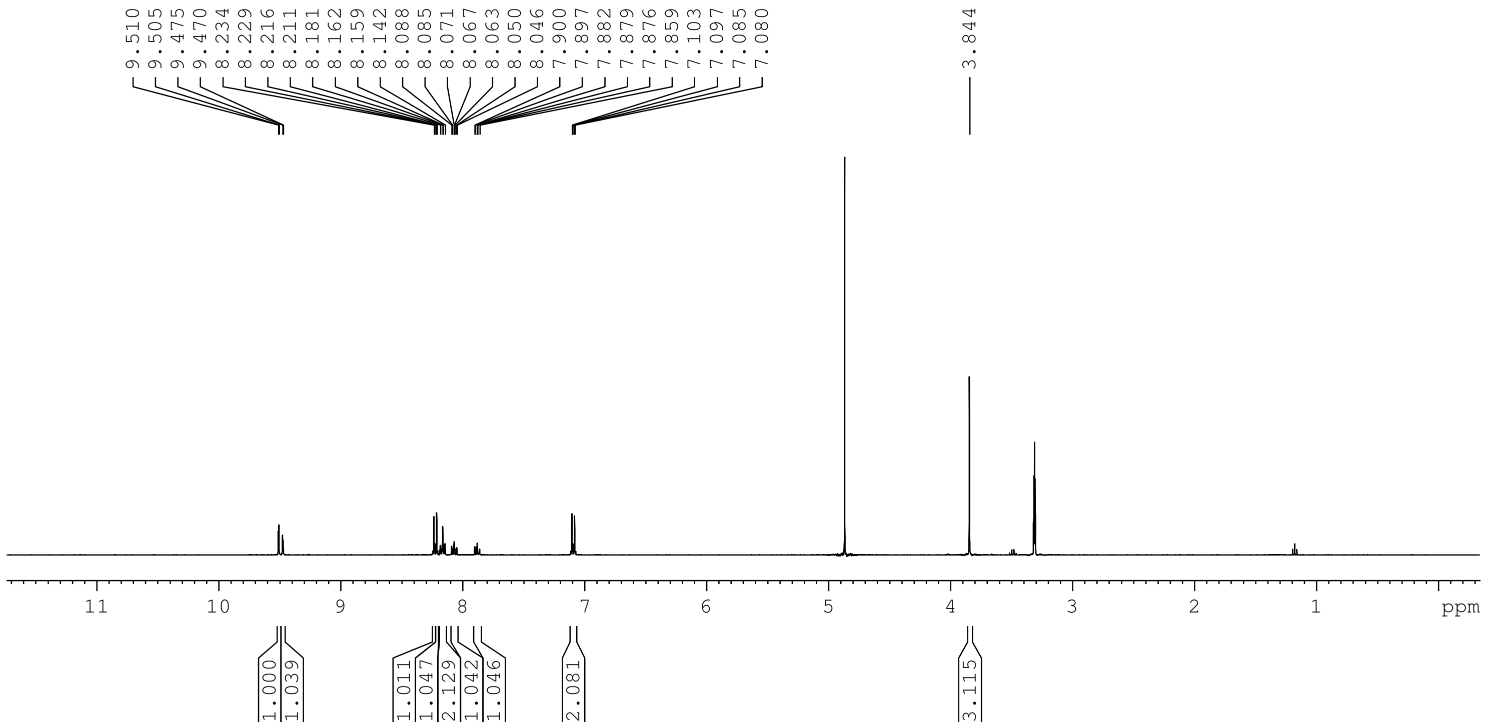

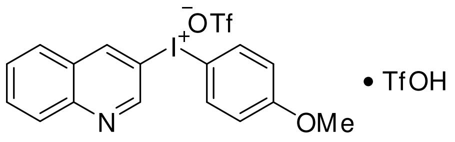

3g'

125 MHZ
MeOD

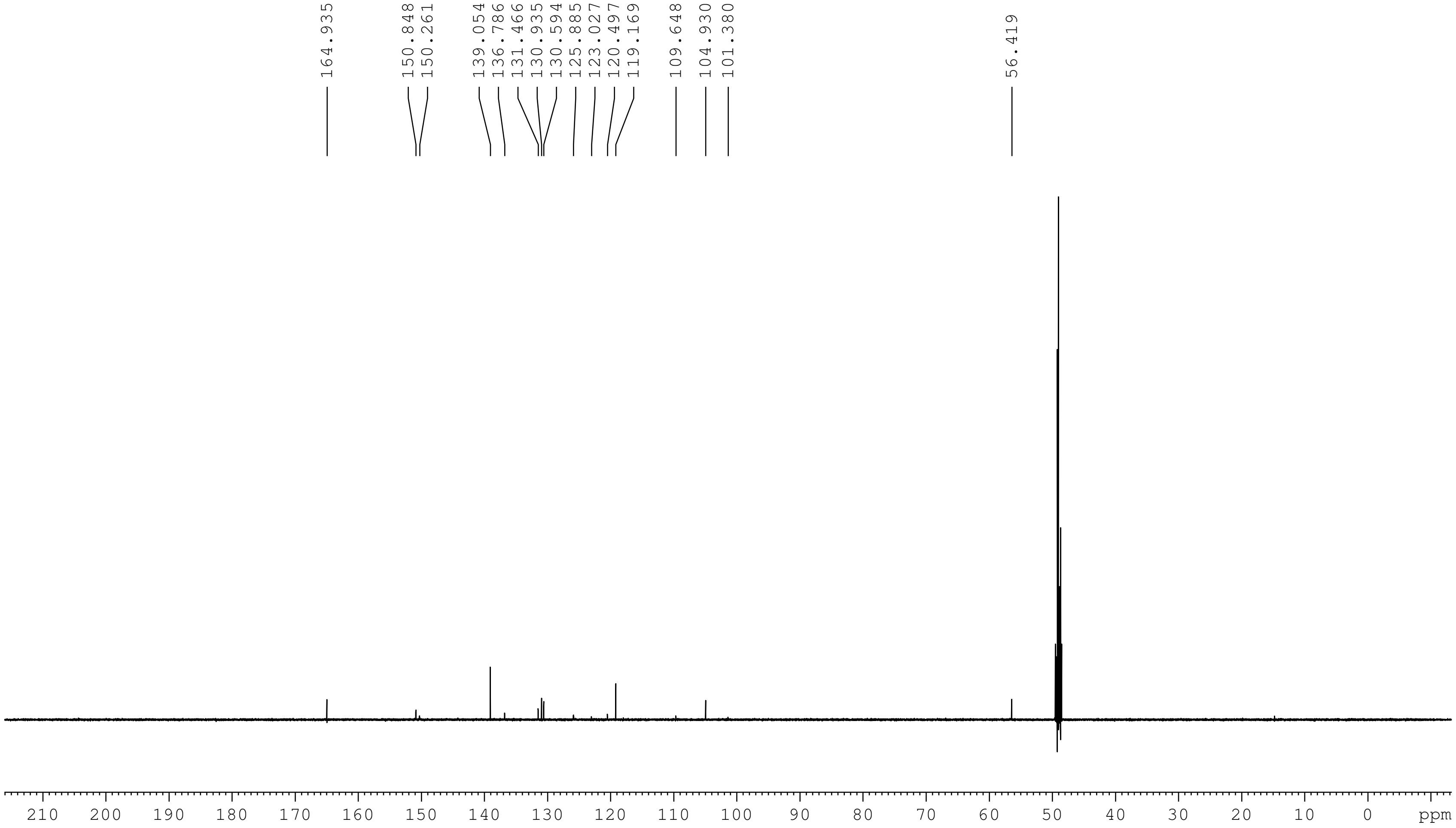

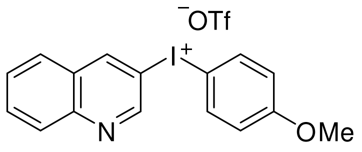

3g

400 MHz
MeOD

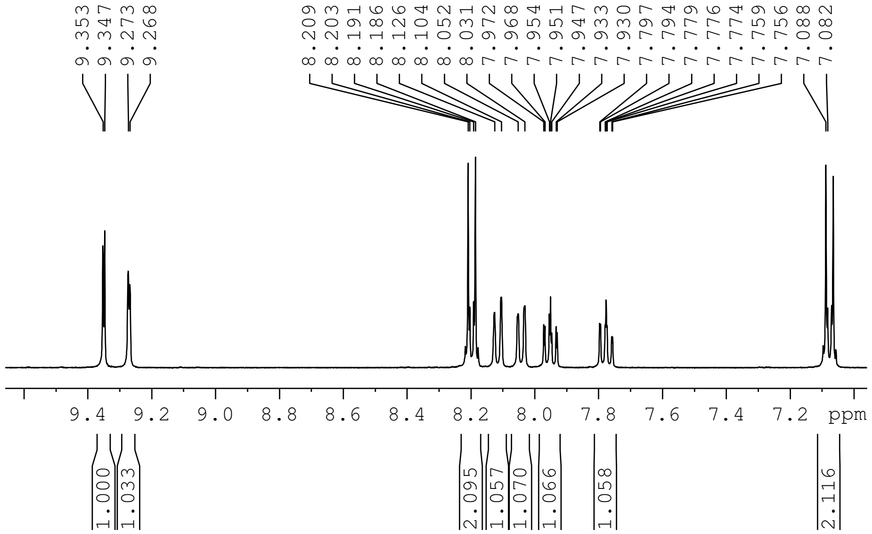

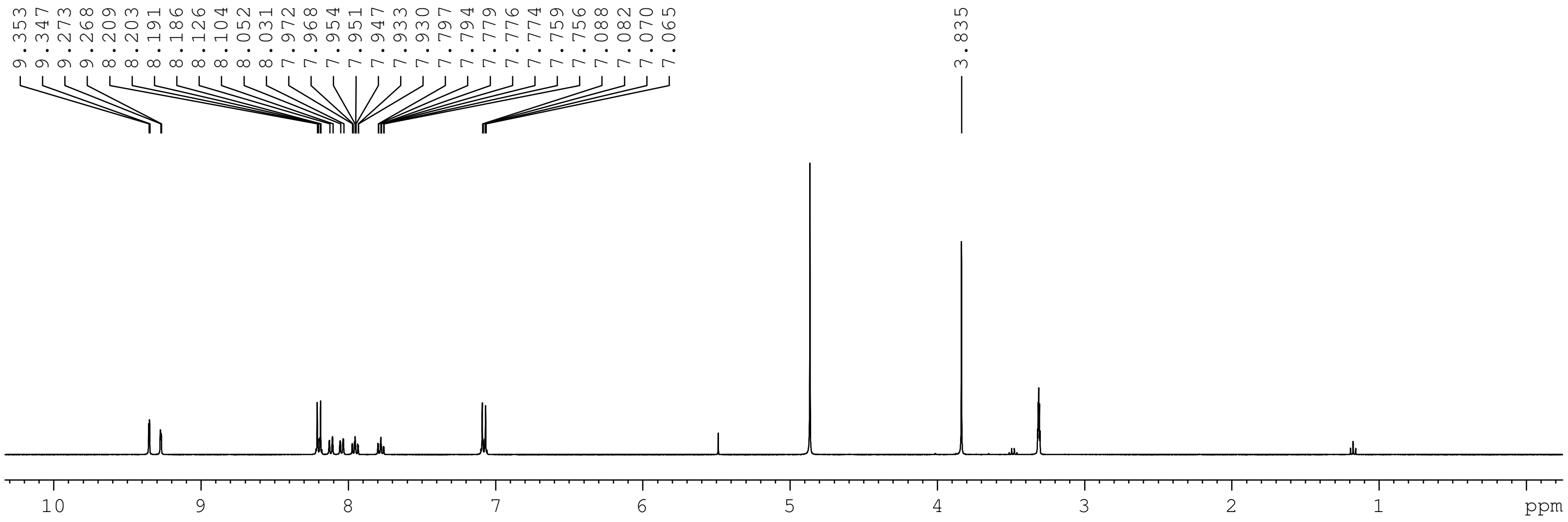

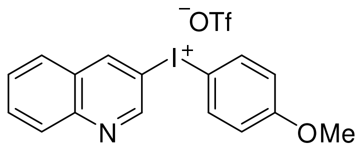

3g

100 MHz
MeOD

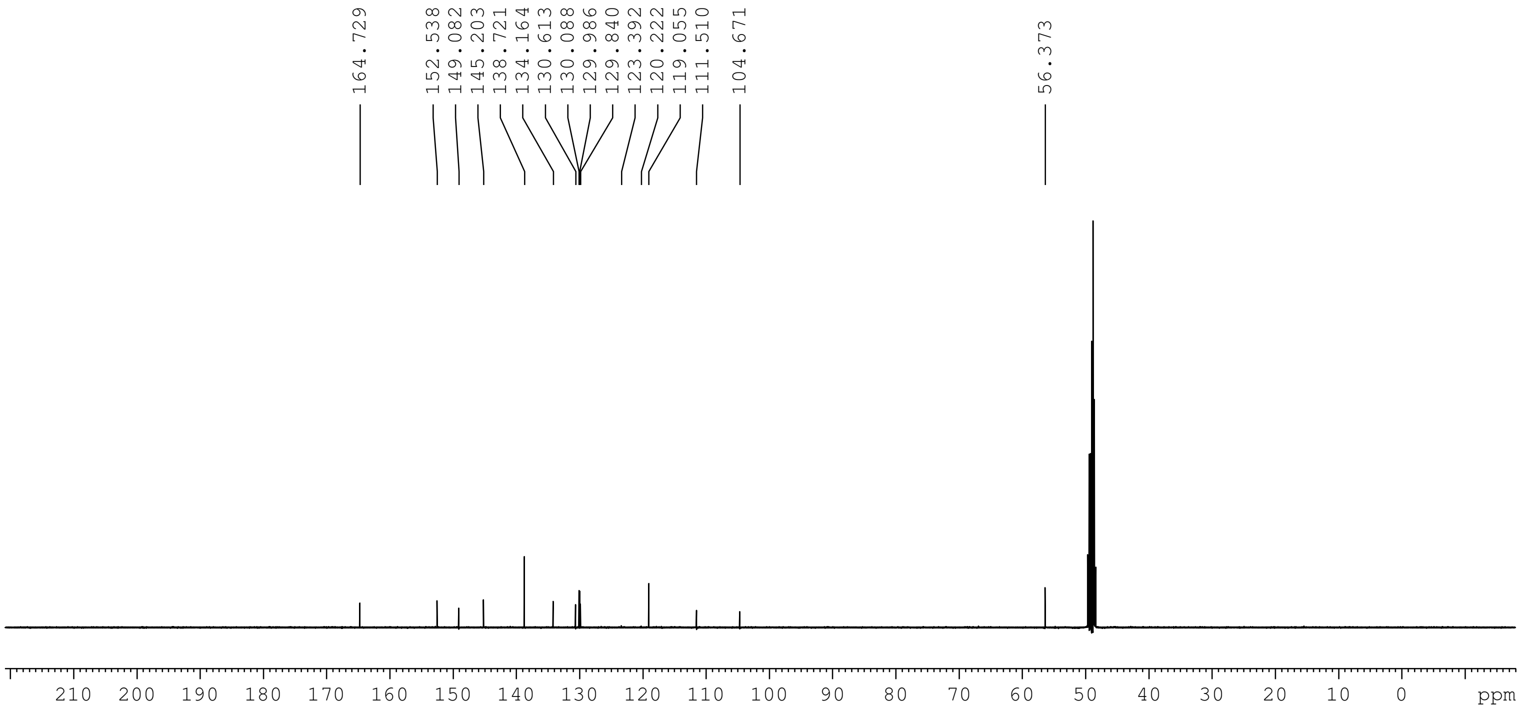

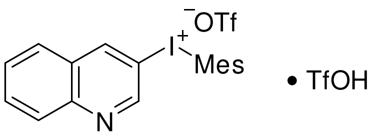

3h'

400 MHz
MeOD

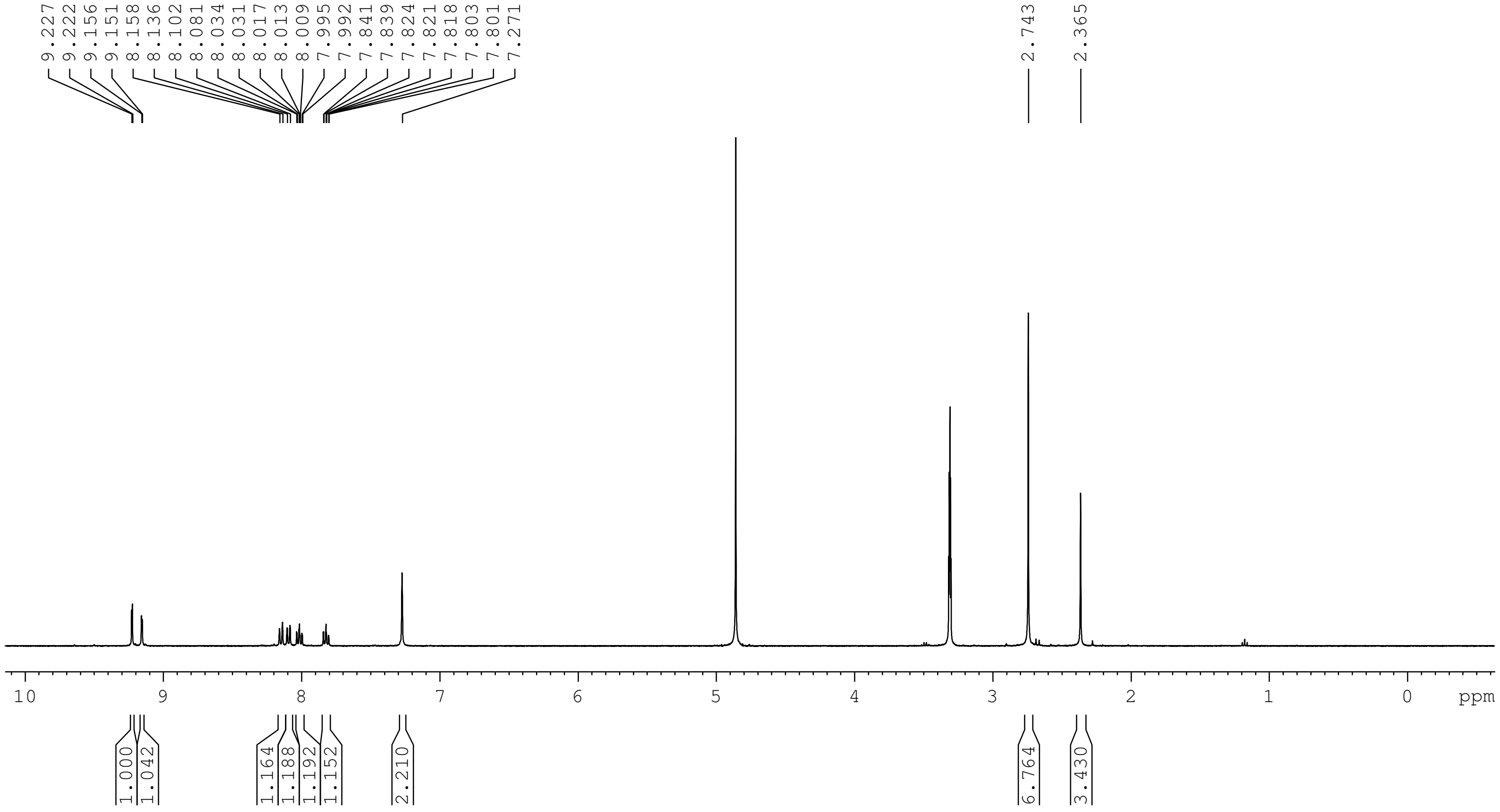

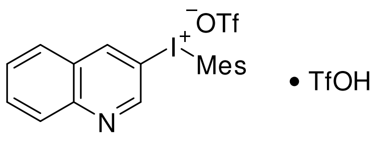

3h'

100 MHz
MeOD

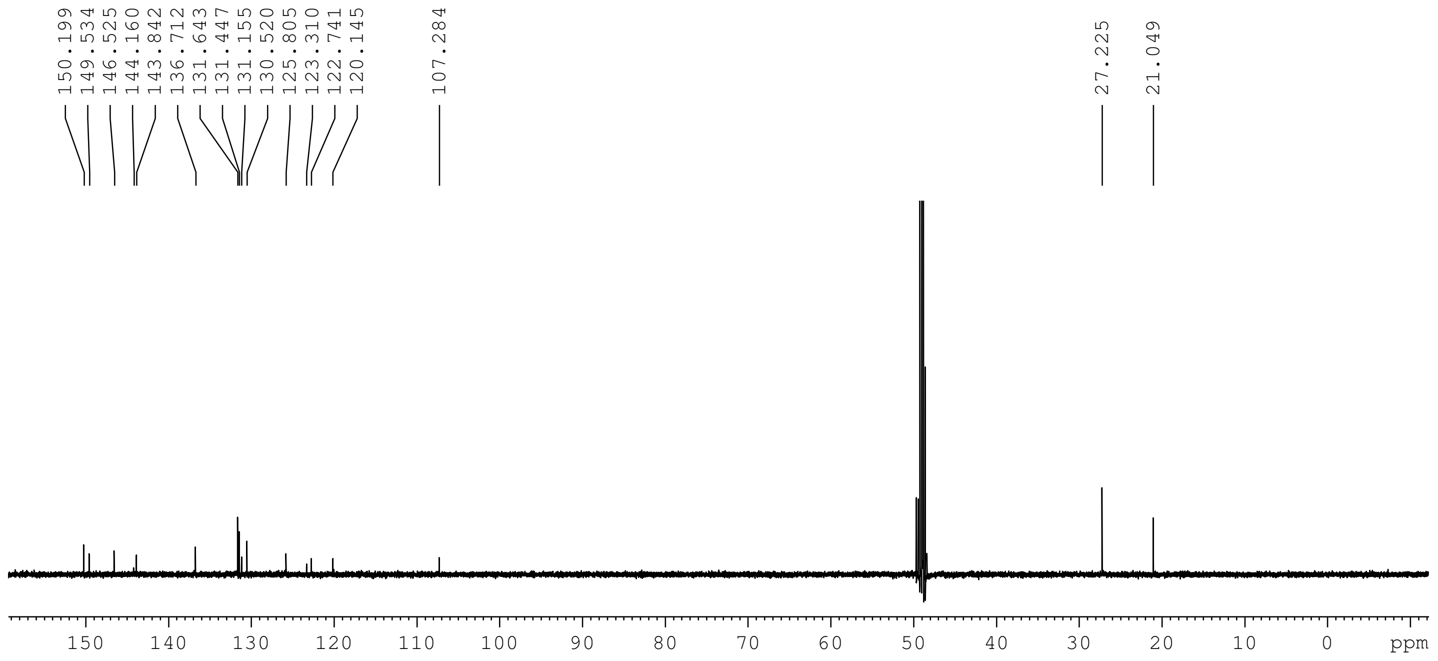

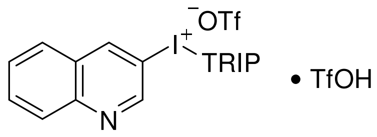

3i'

400 MHz
MeOD

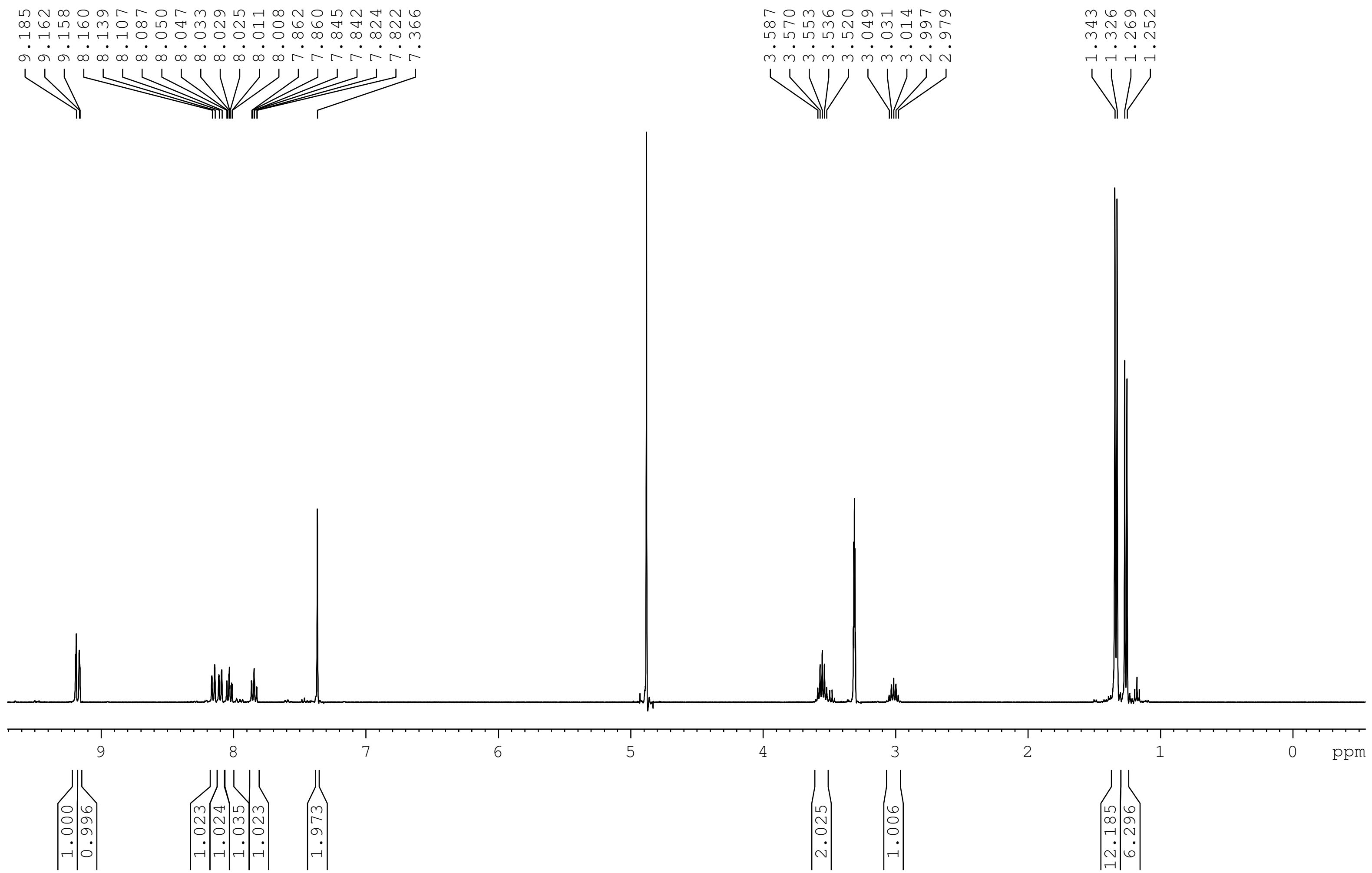

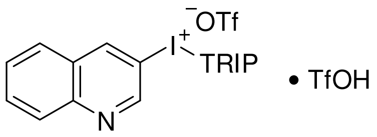

3i'

100 MHz
MeOD

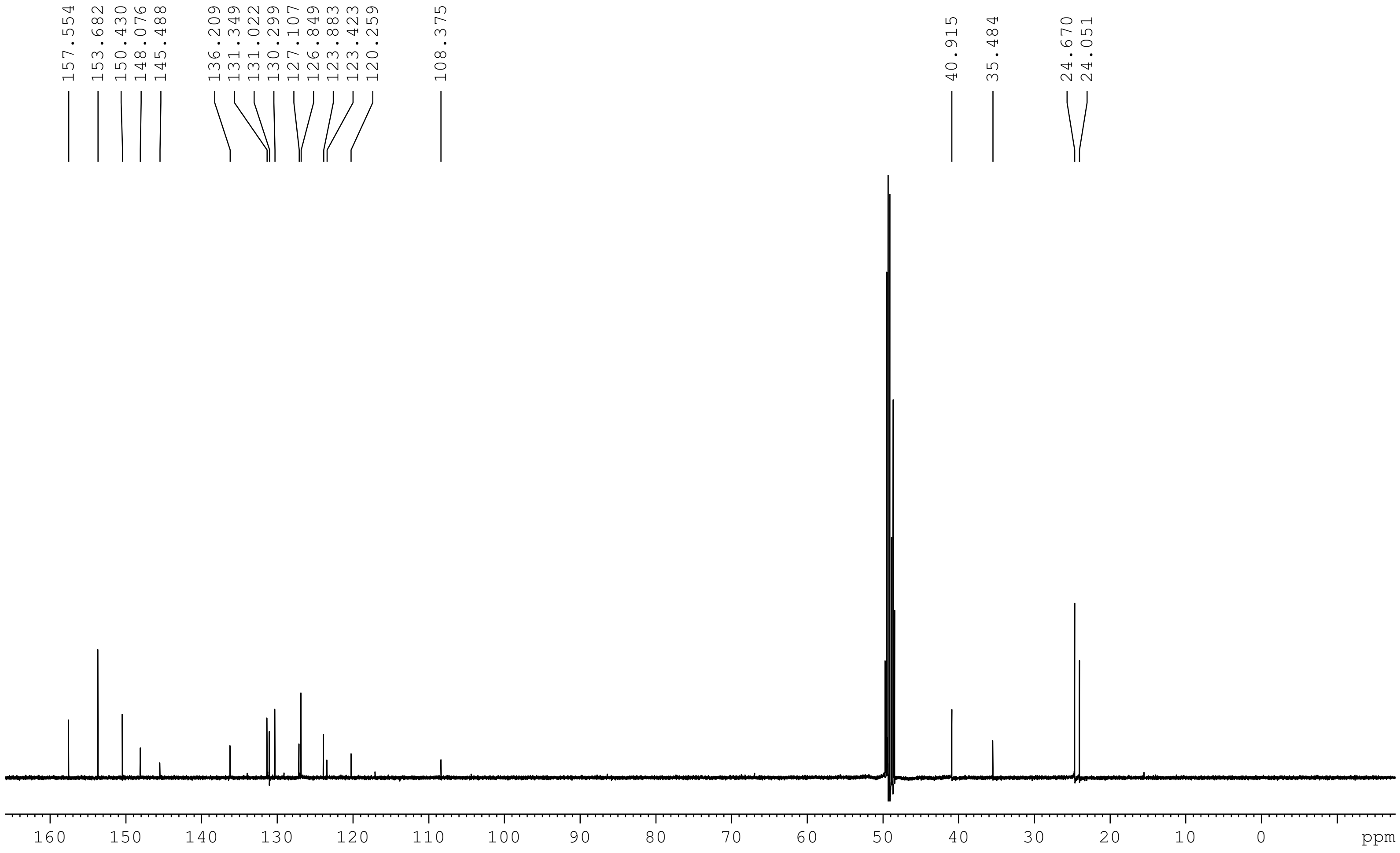

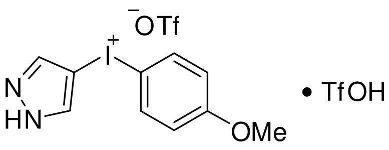

3j'

400 MHz
MeOD

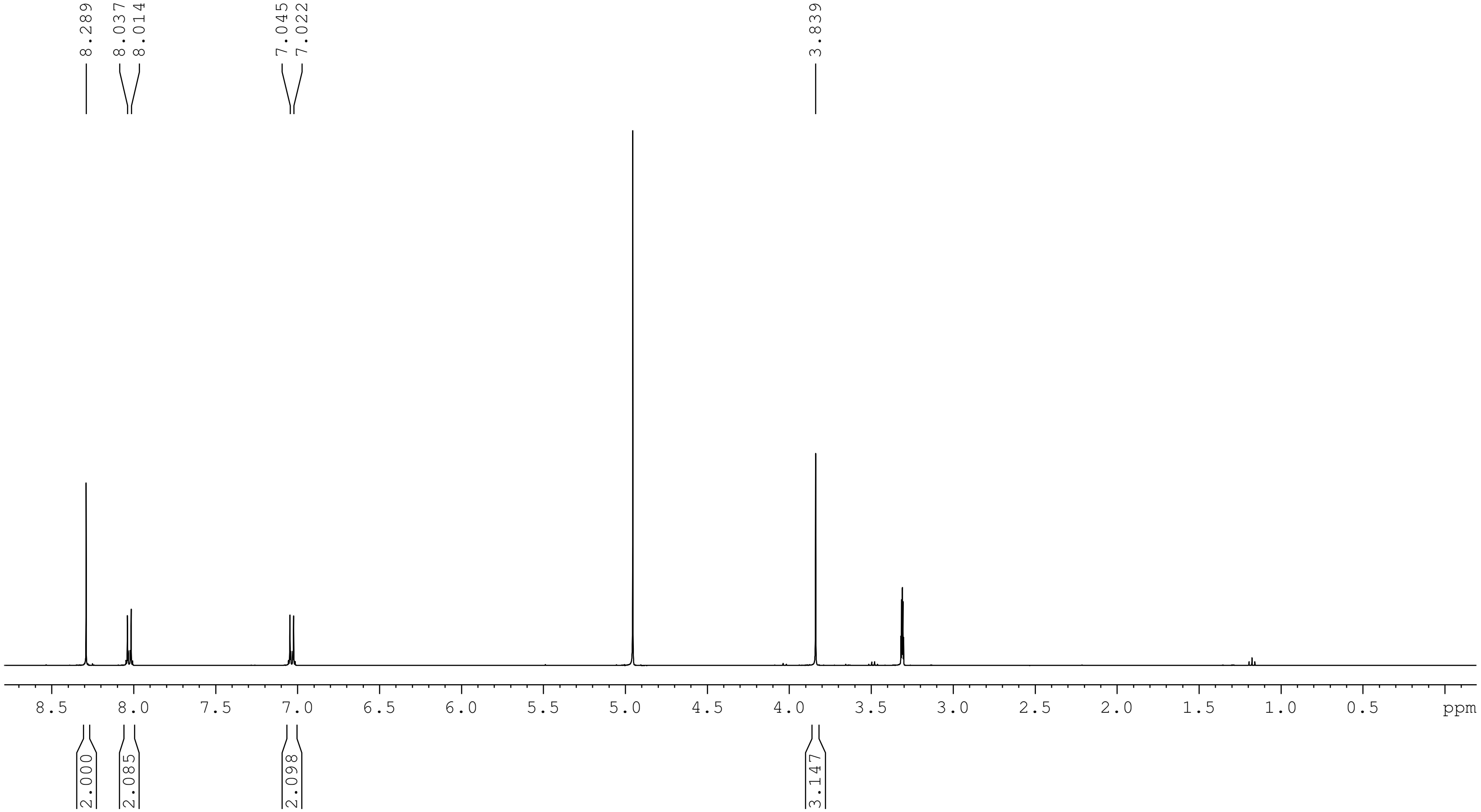

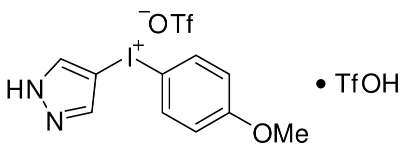

3j'

100 MHz
MeOD

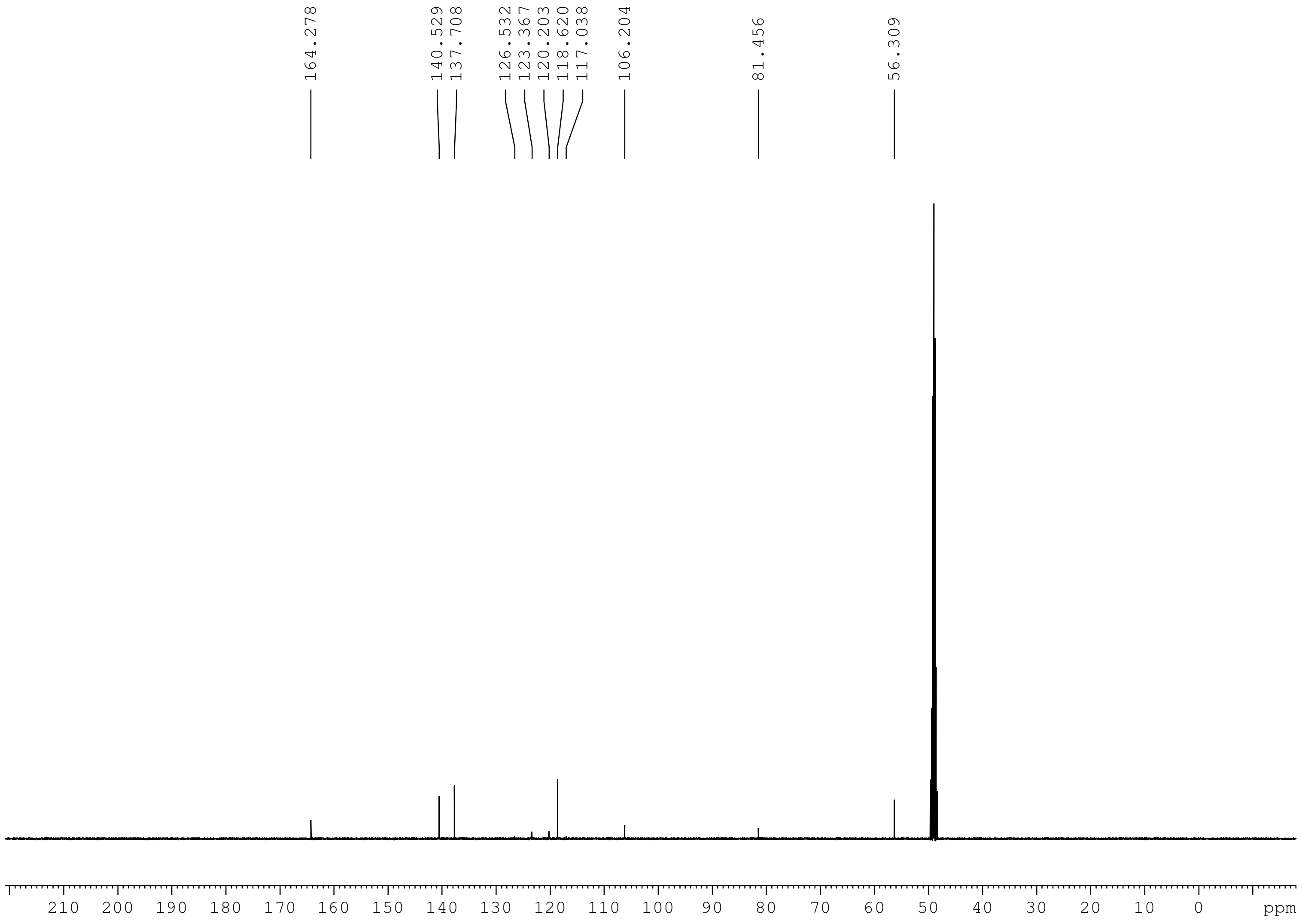

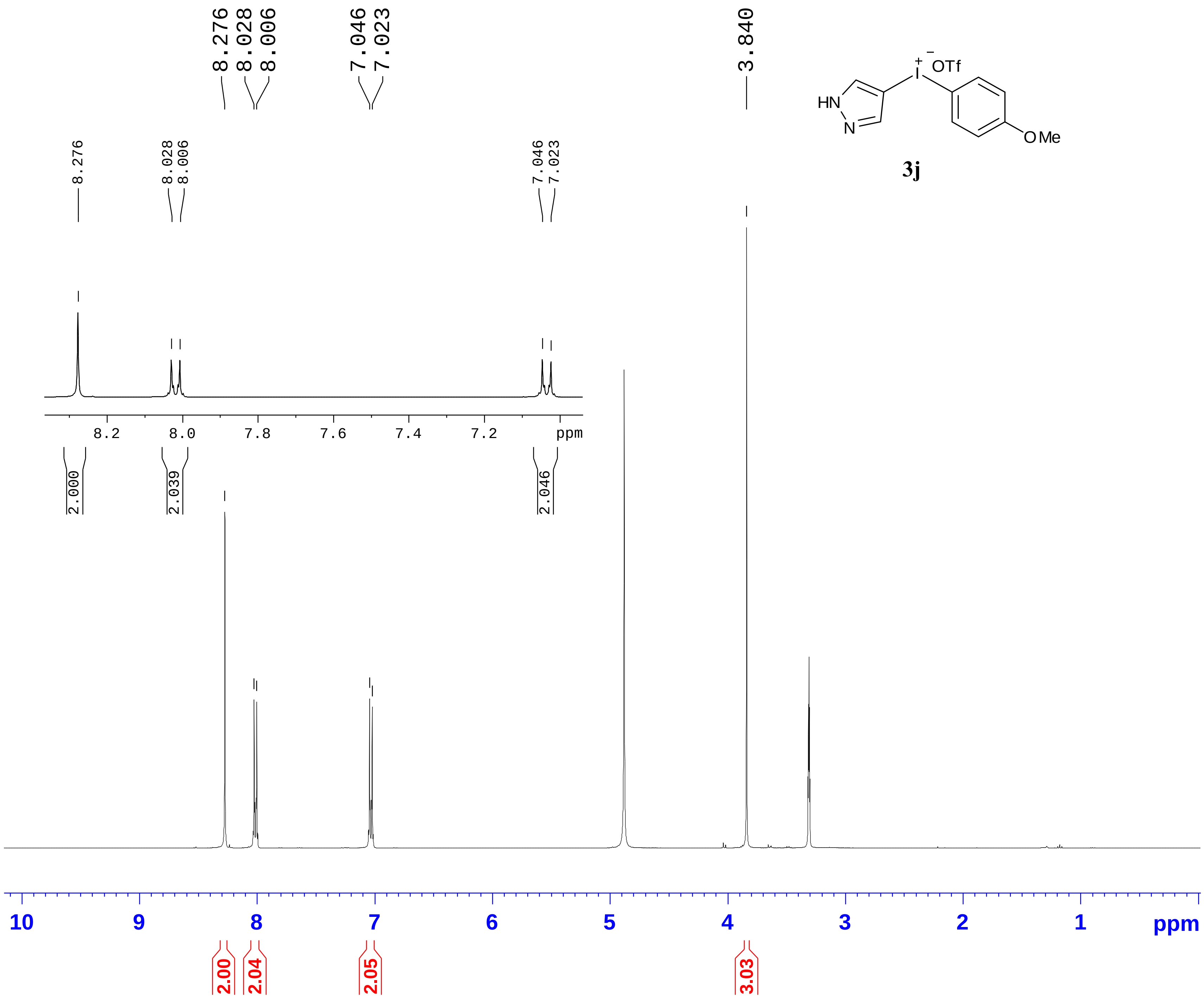
8.276
8.028
8.006
7.046
7.023
3.840
HN
N
I
OTf
OMe
3j
8.2
8.0
7.8
7.6
7.4
7.2
ppm
2.000
2.039
2.046
10
9
8
7
6
5
4
3
2
1
ppm
2.00
2.04
2.05
3.03

400 MHz
MeOD

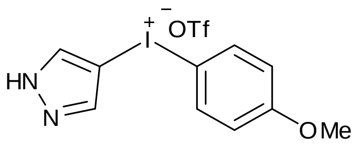

3j

100 MHz
MeOD

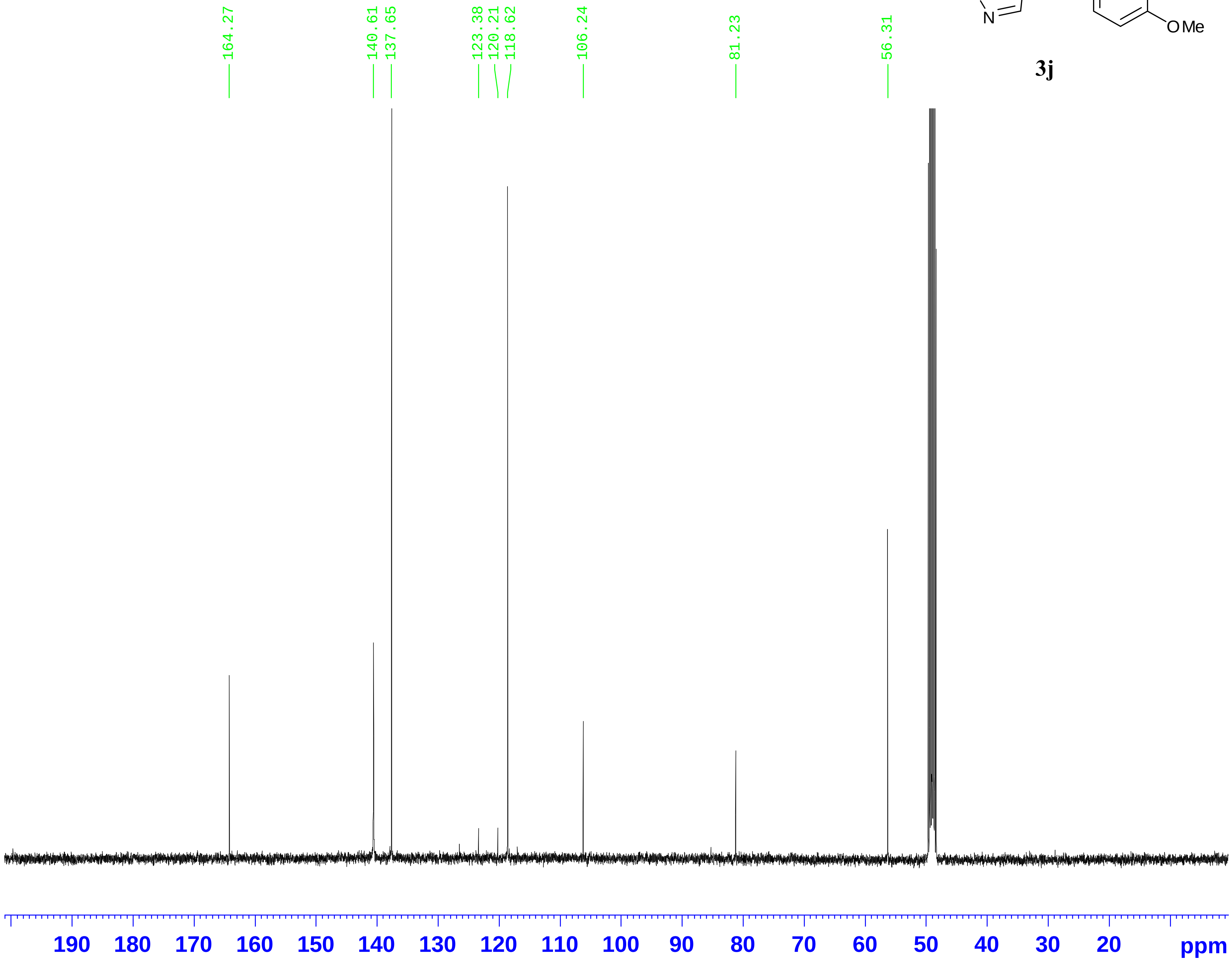

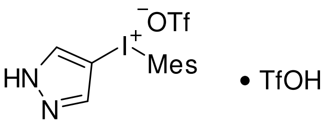

3k'

400 MHz
MeOD

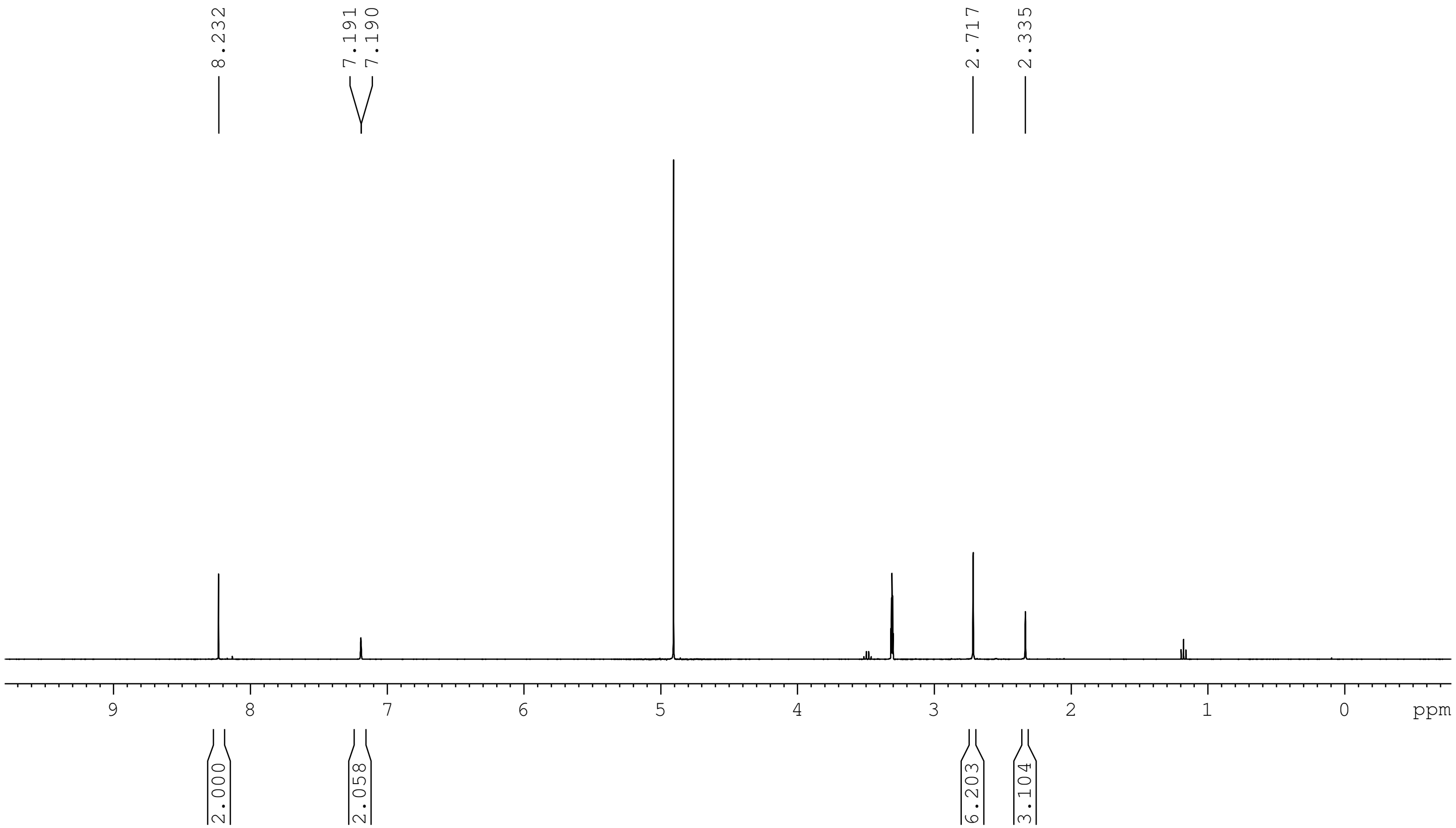

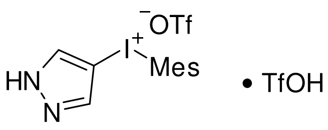

3k'

100 MHz
MeOD

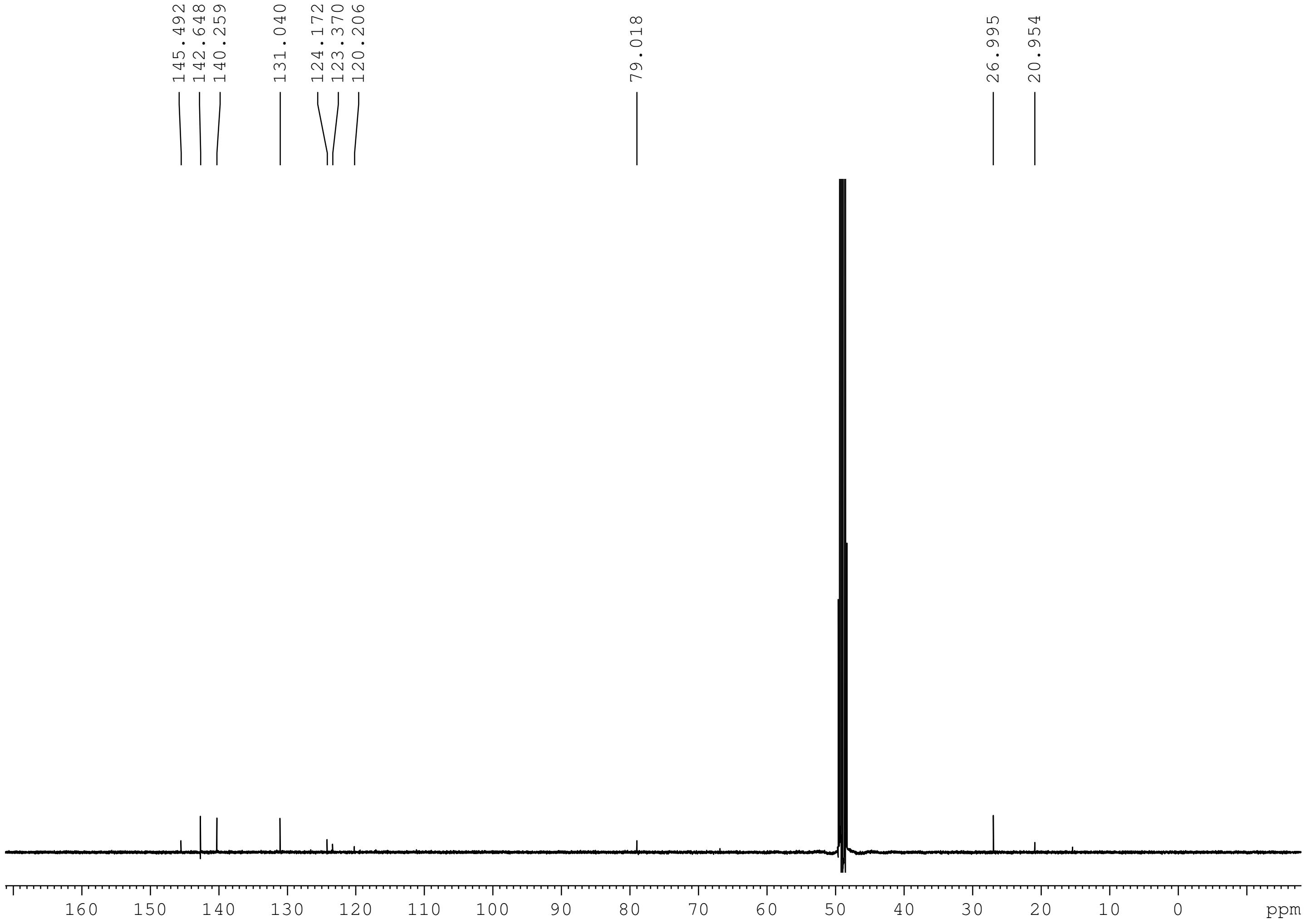

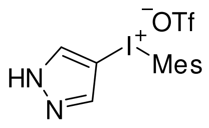

3k

400 MHz
MeOD

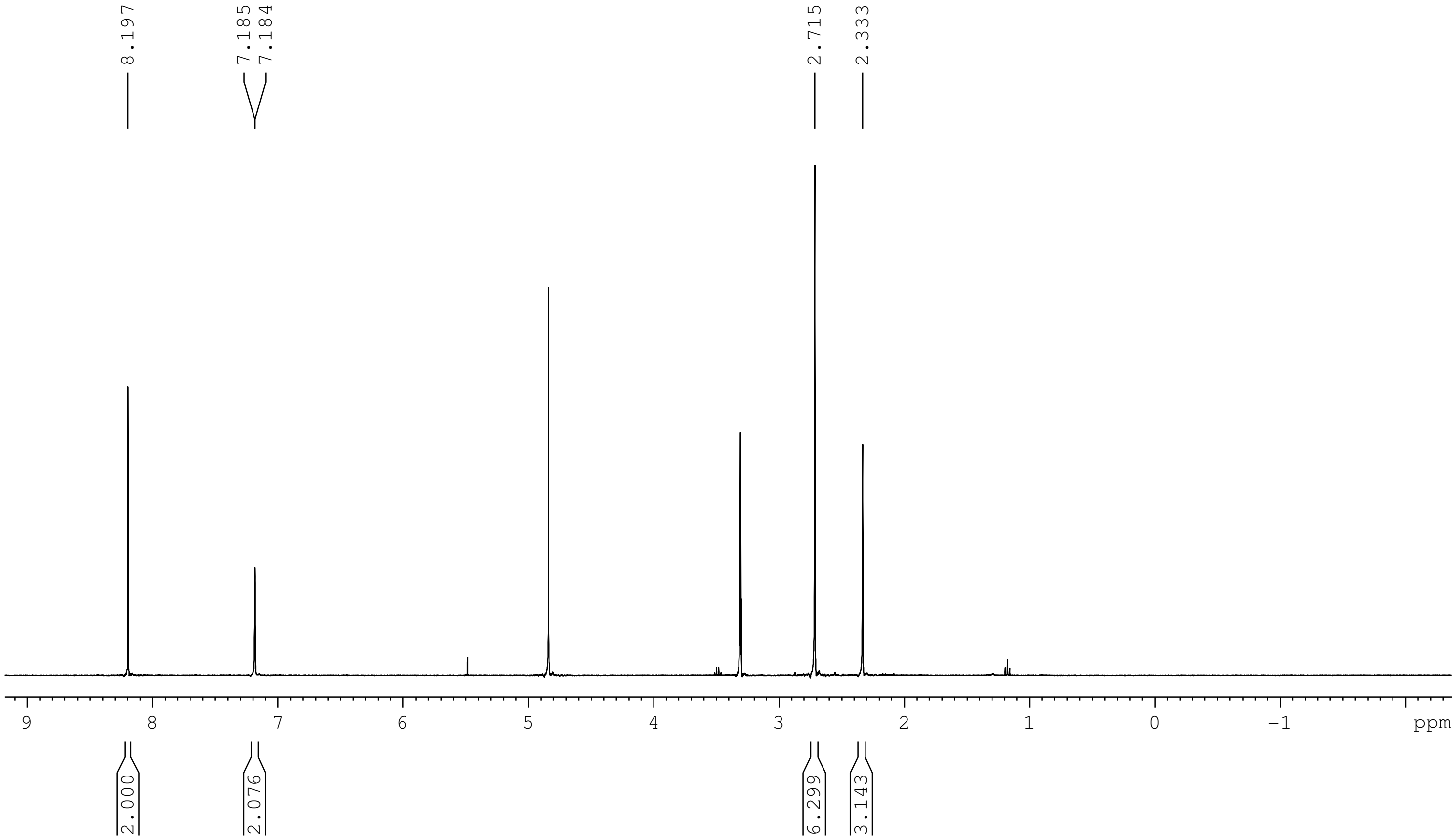

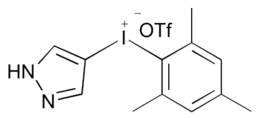

3k

100 MHz
MeOD

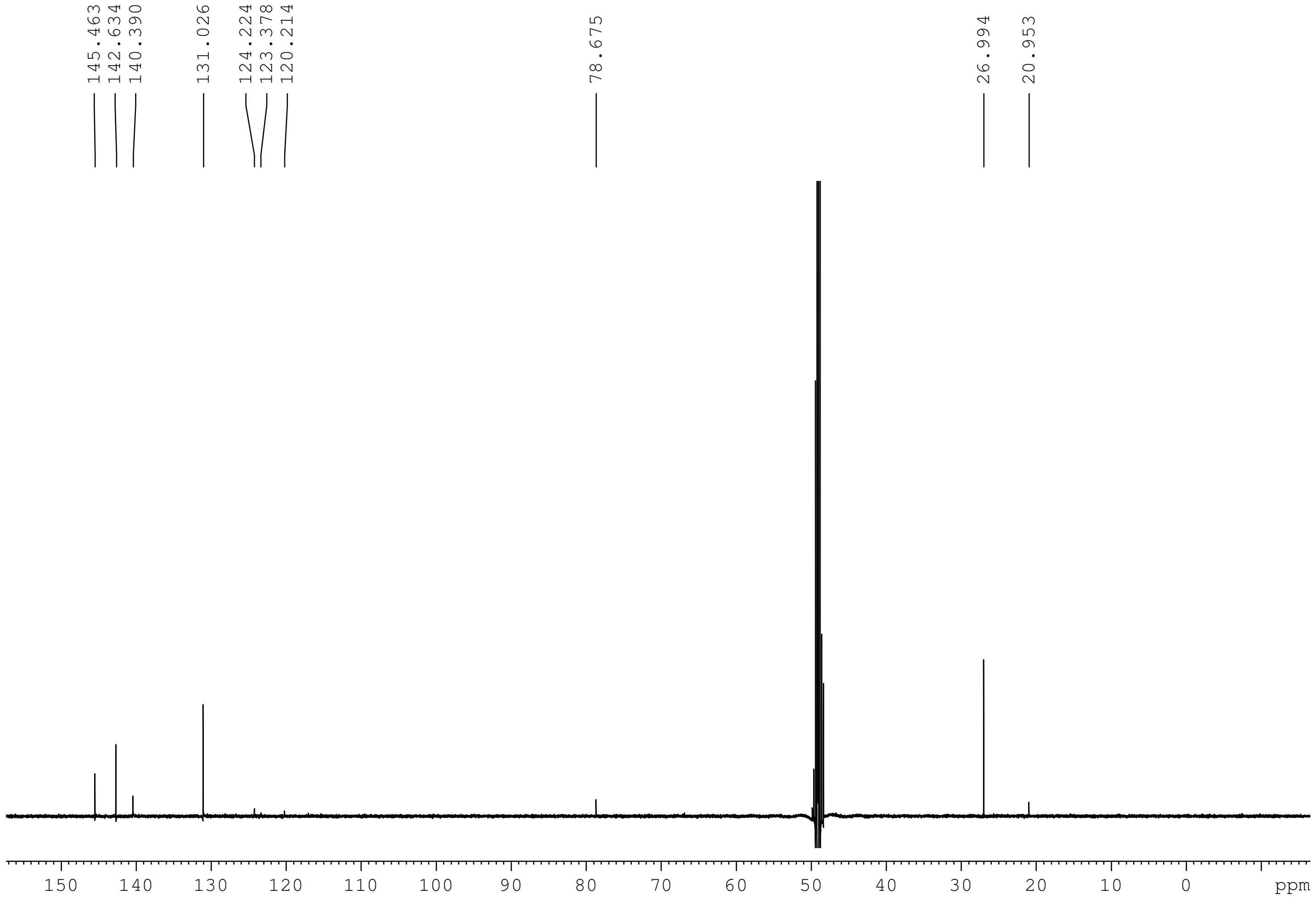

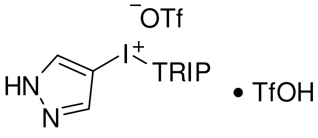

3l'

400 MHz
MeOD

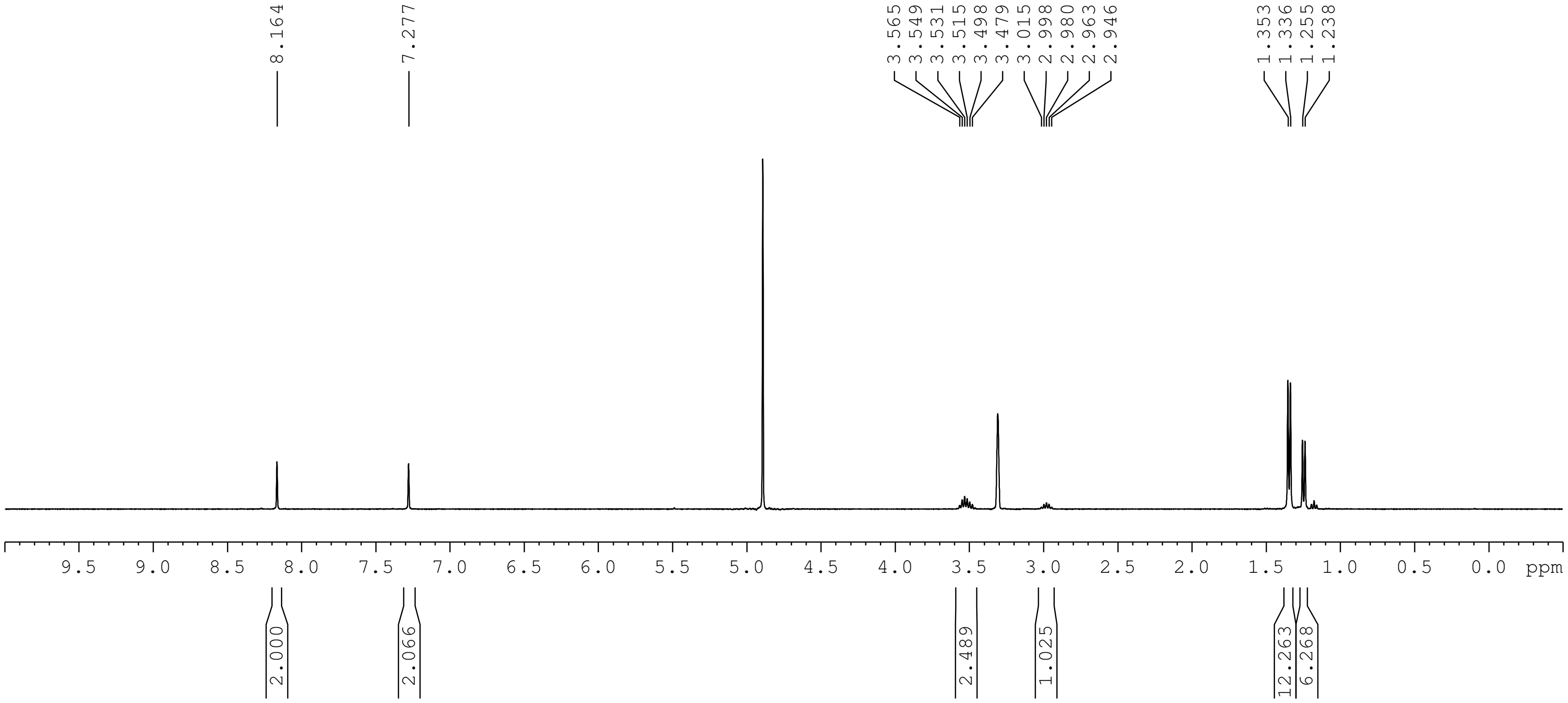

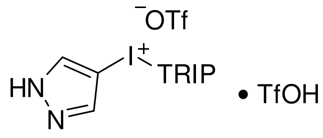

3l'

100 MHz
MeOD

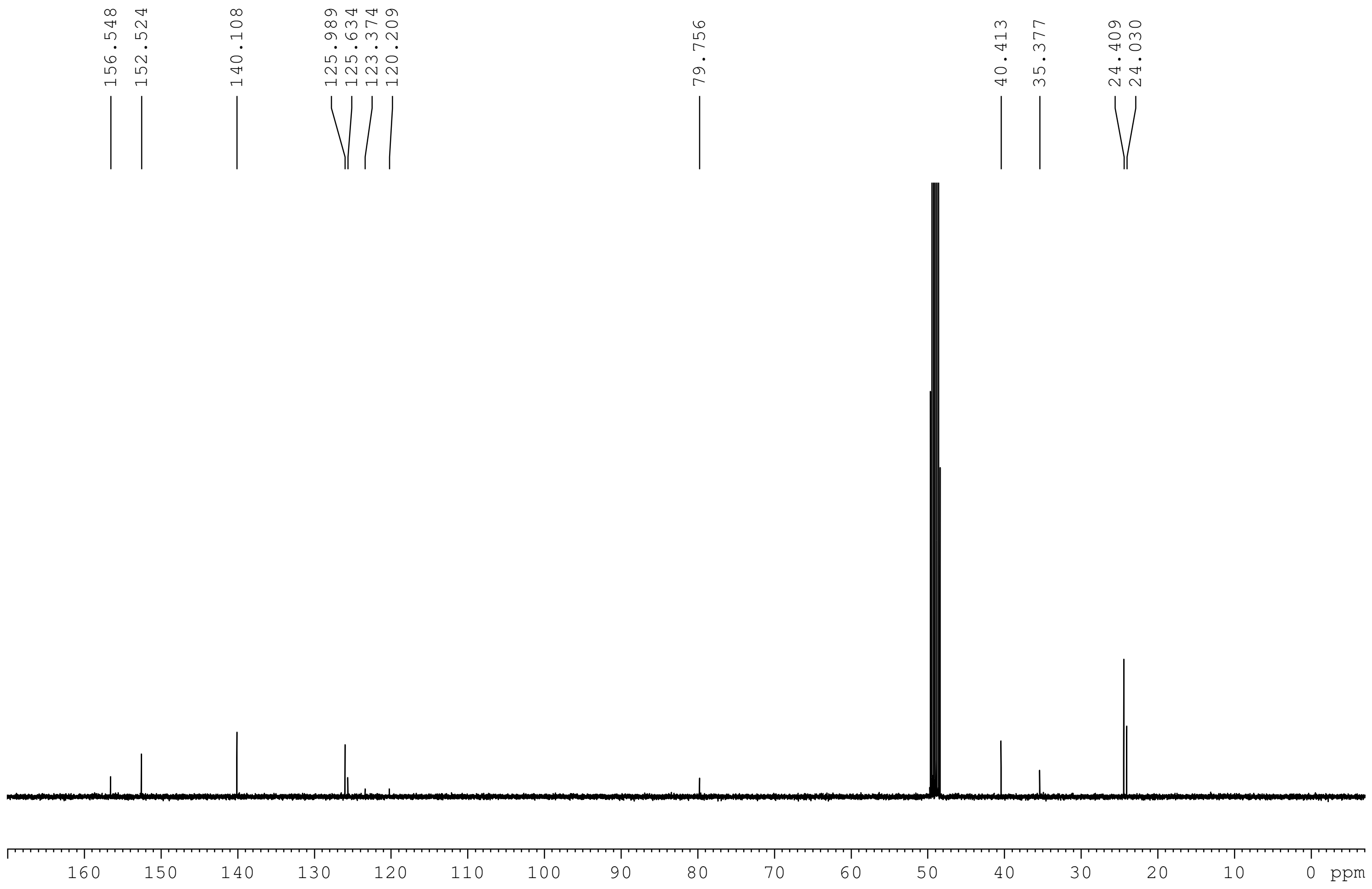

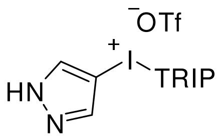

3l

400 MHz
MeOD

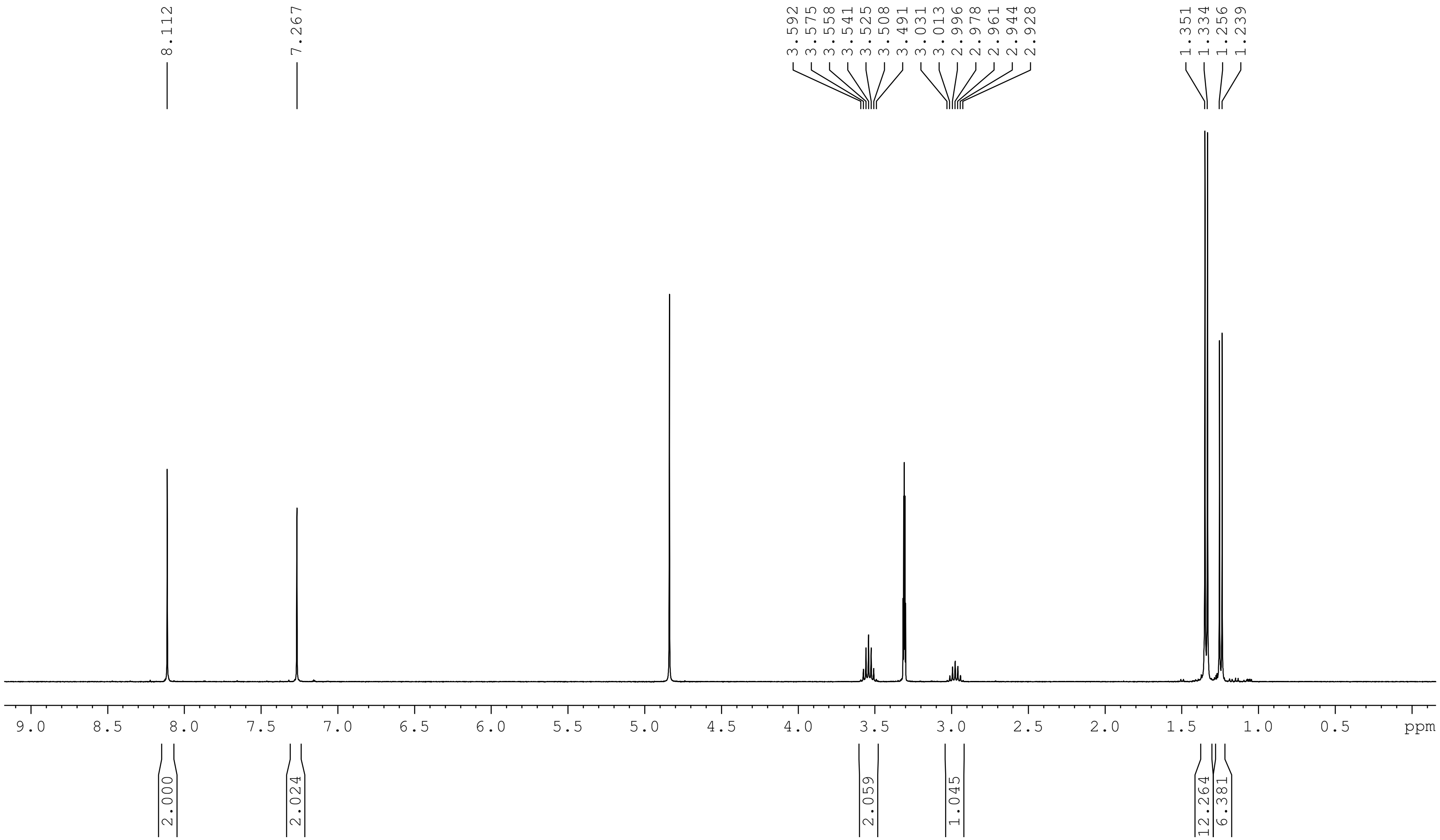

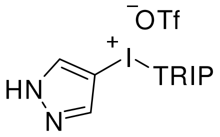

3l

100 MHz
MeOD

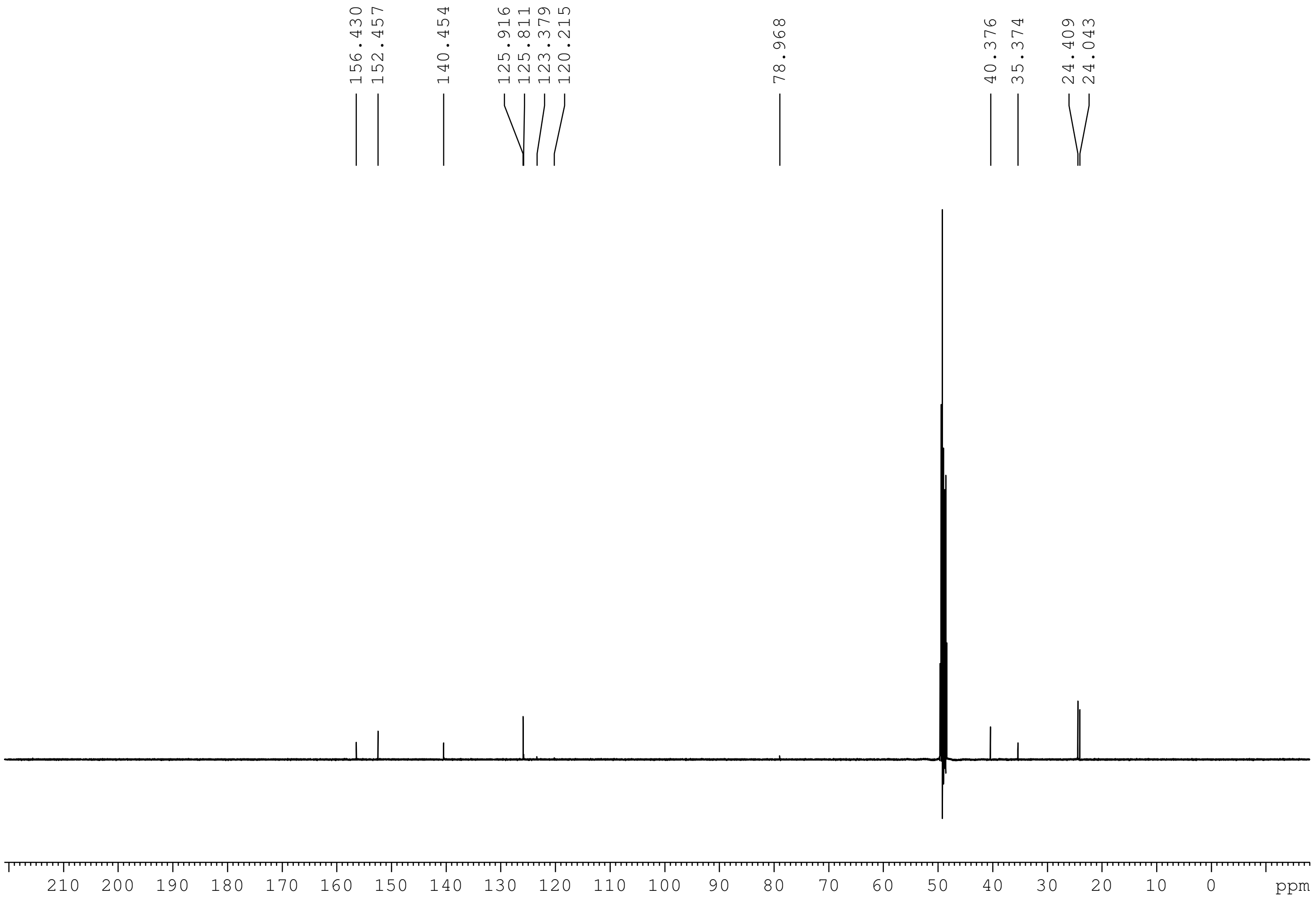

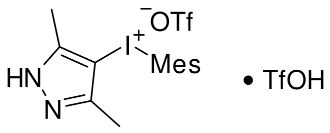

3m'

400 MHz
MeOD

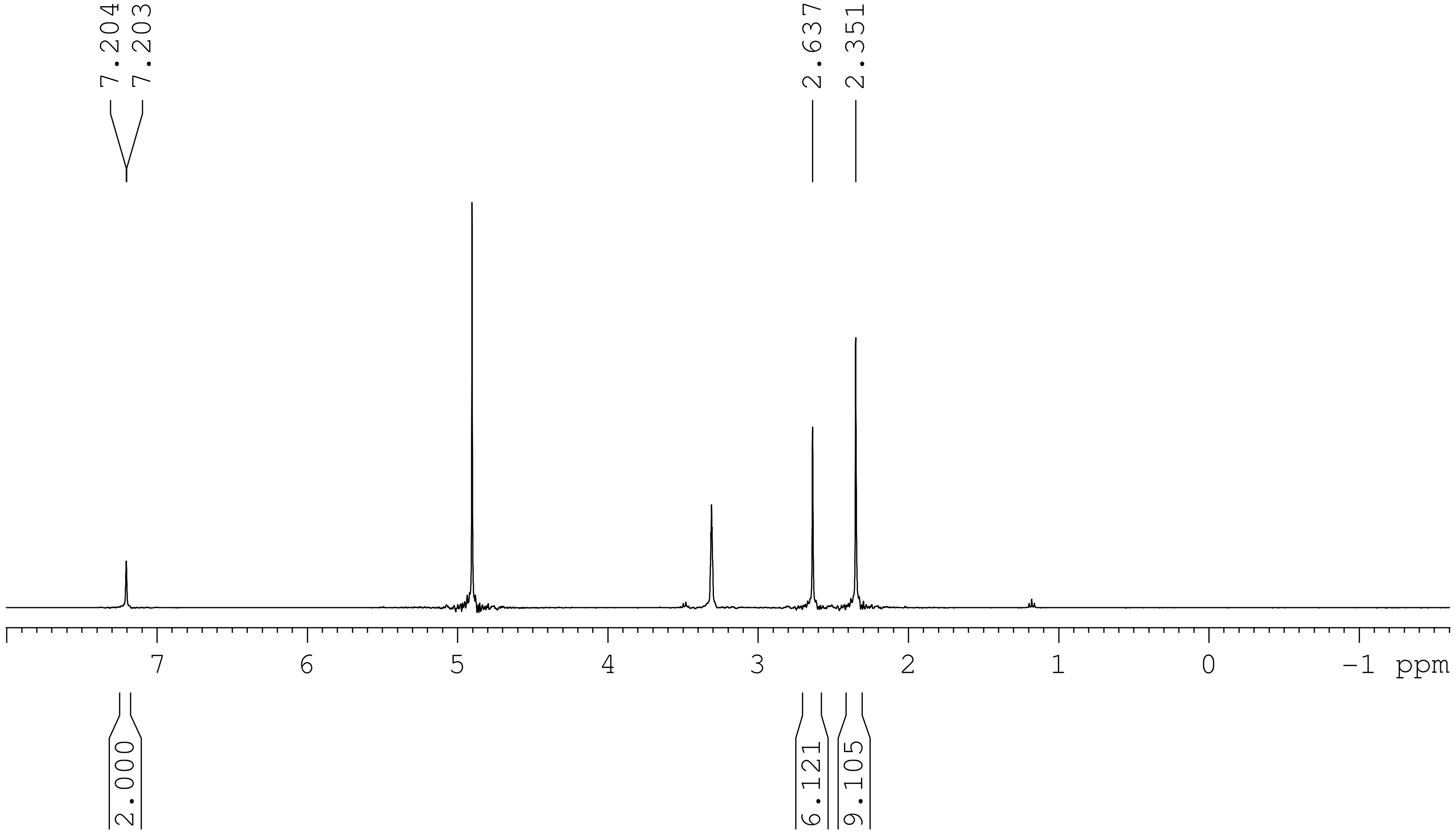

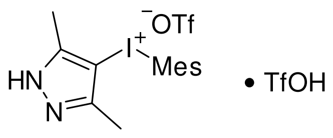

3m'

100 MHz
MeOD

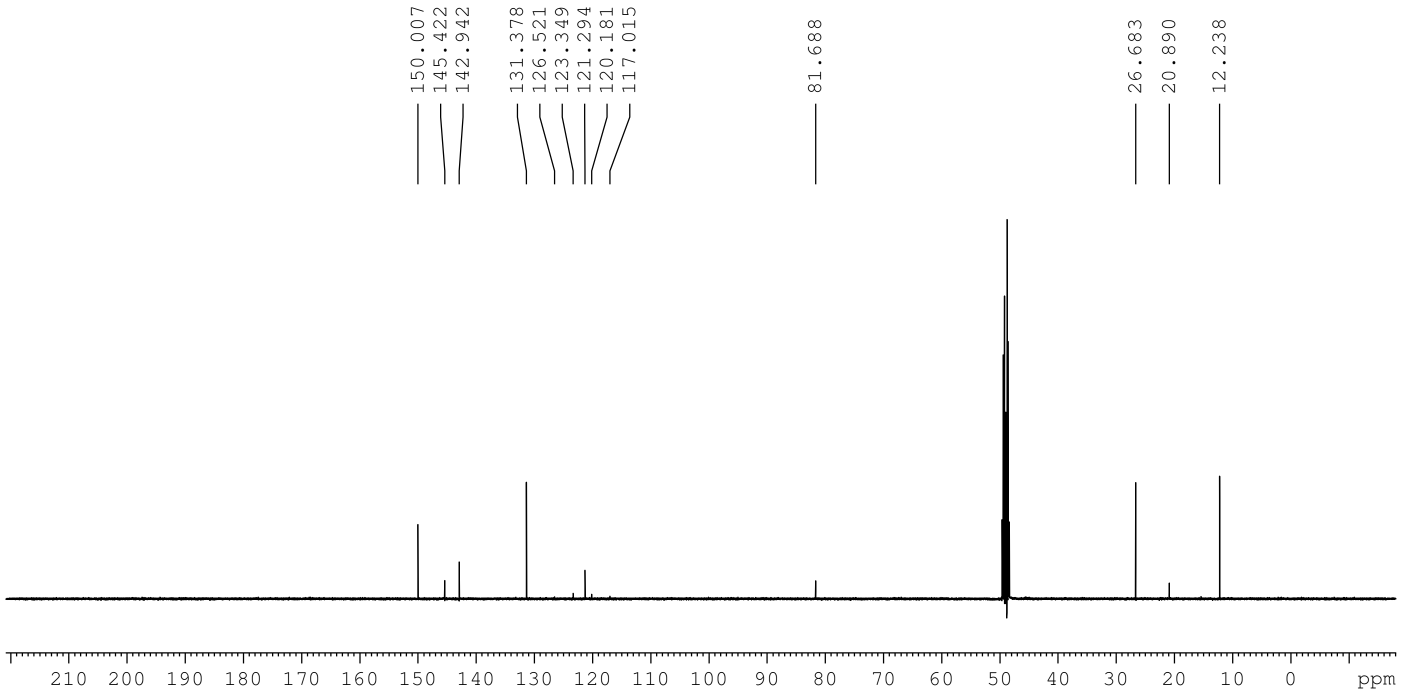

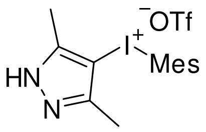

3m

400 MHz
MeOD

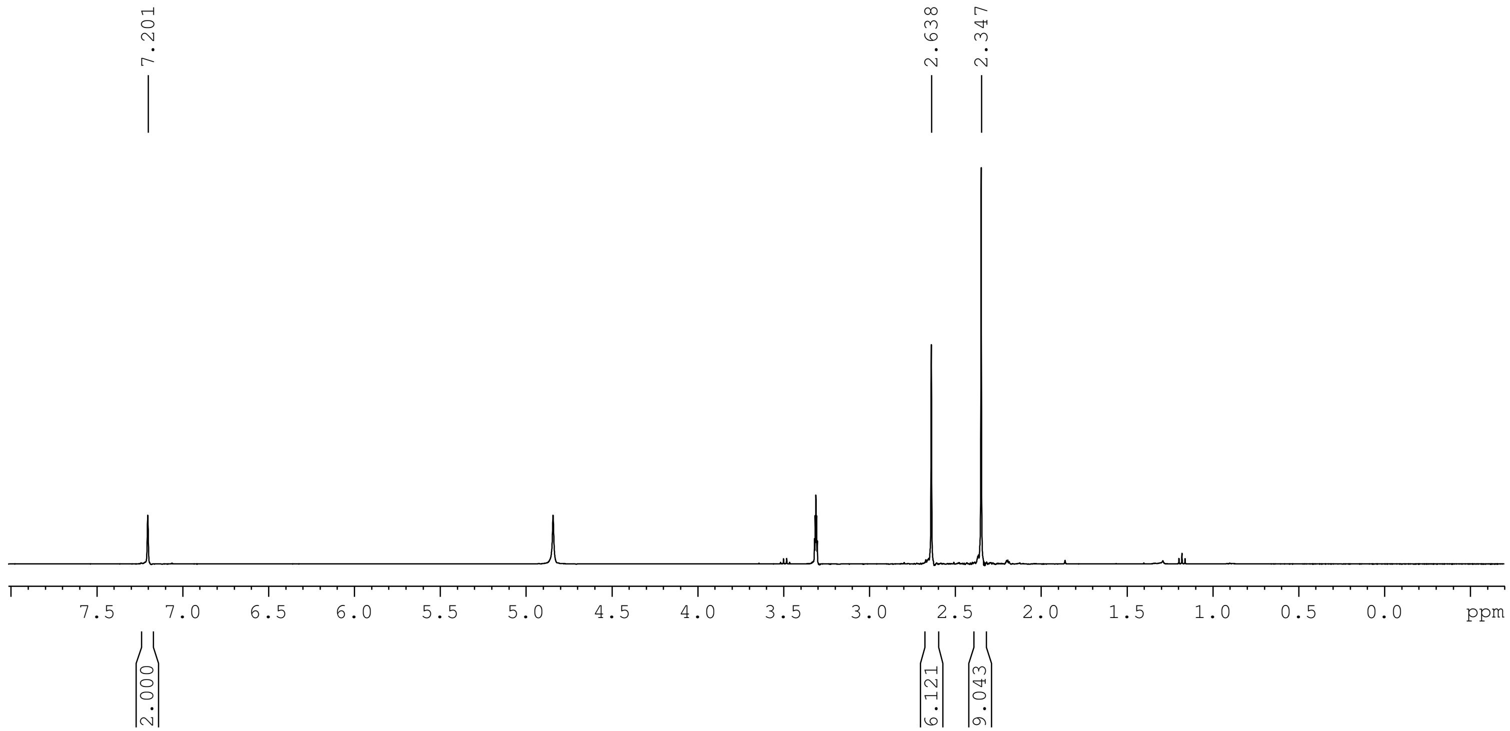

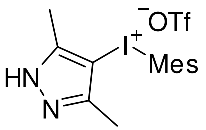

3m

400 MHz
MeOD

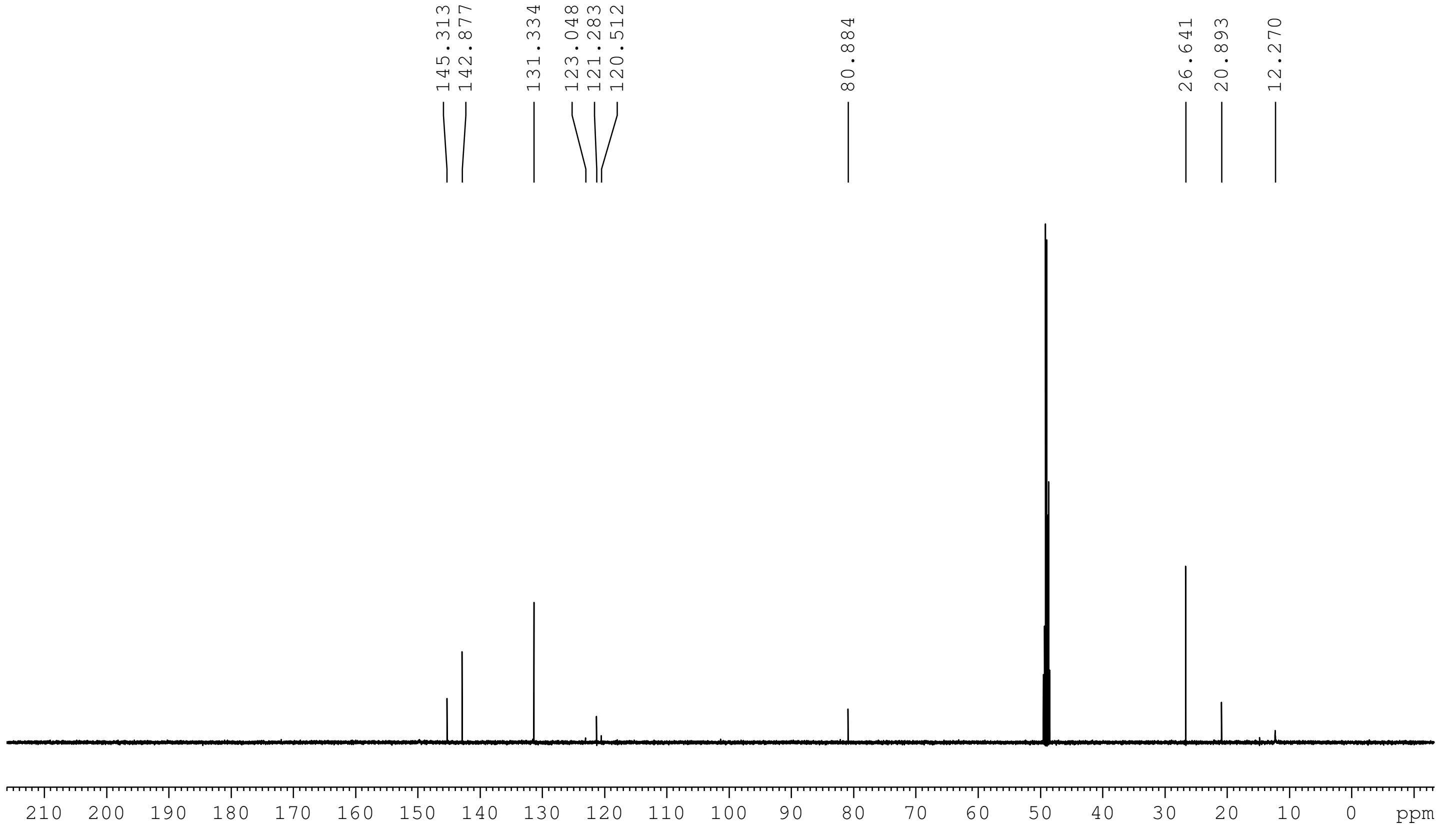

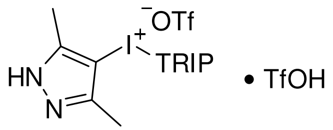

3n'

400 MHz
MeOD

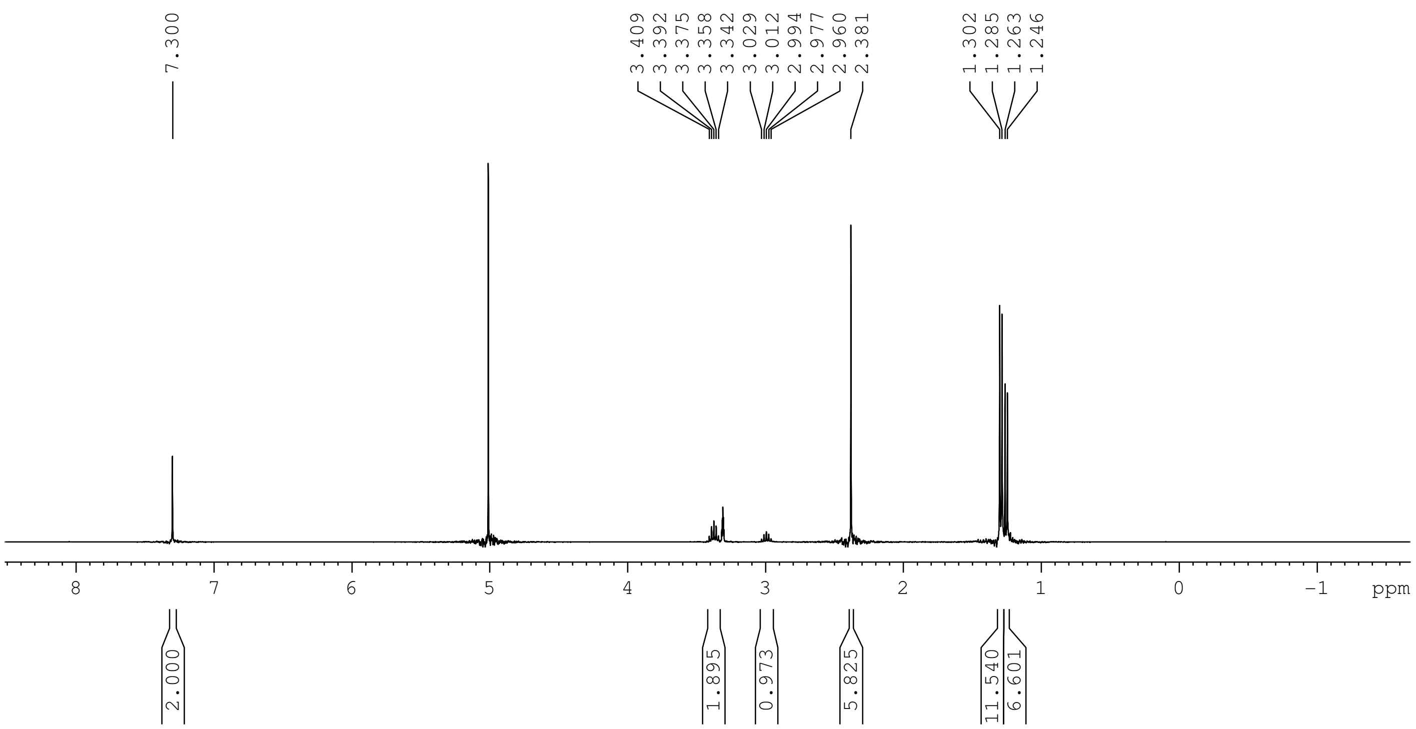

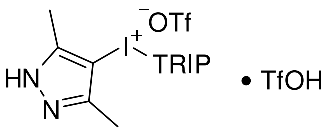

3n'

100 MHz
MeOD

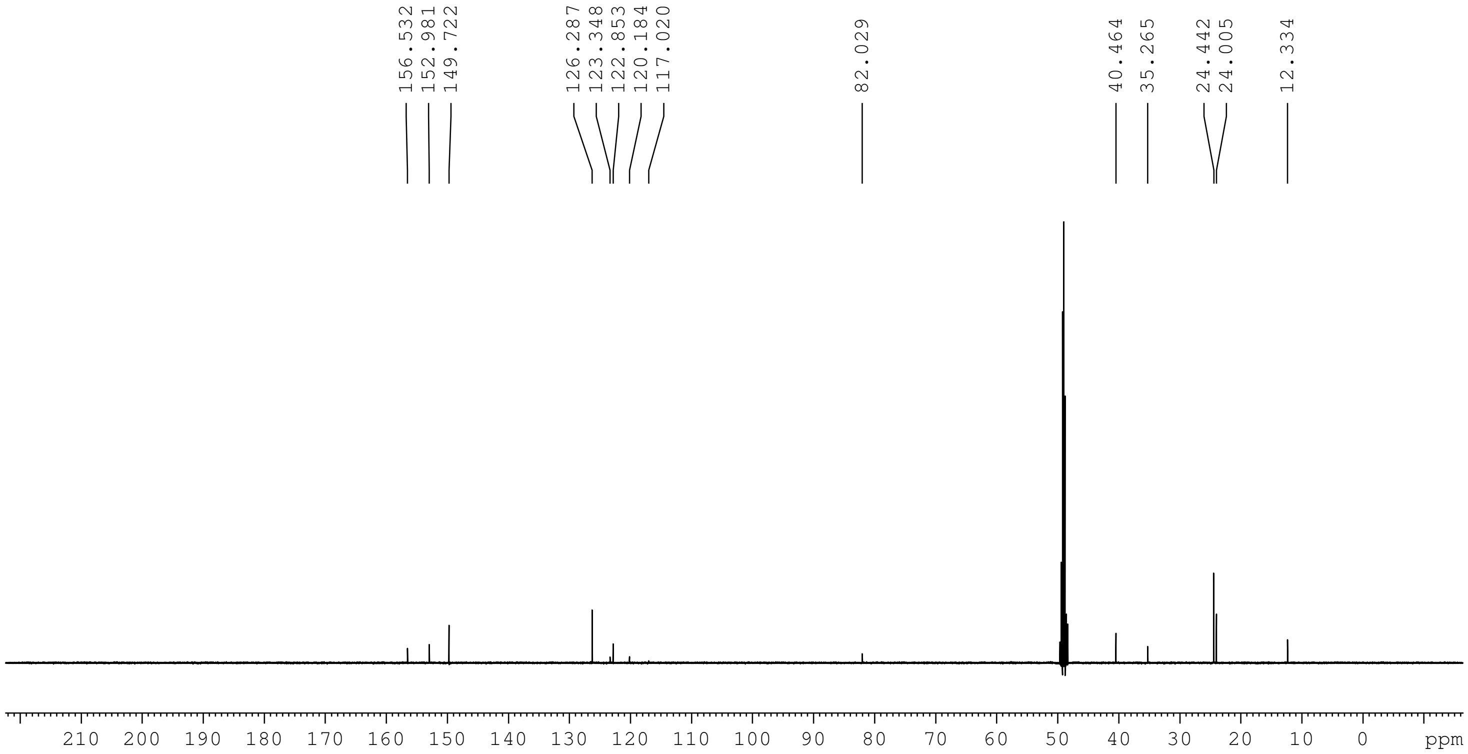

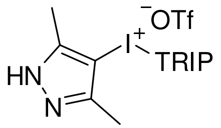

3n

400 MHz
MeOD

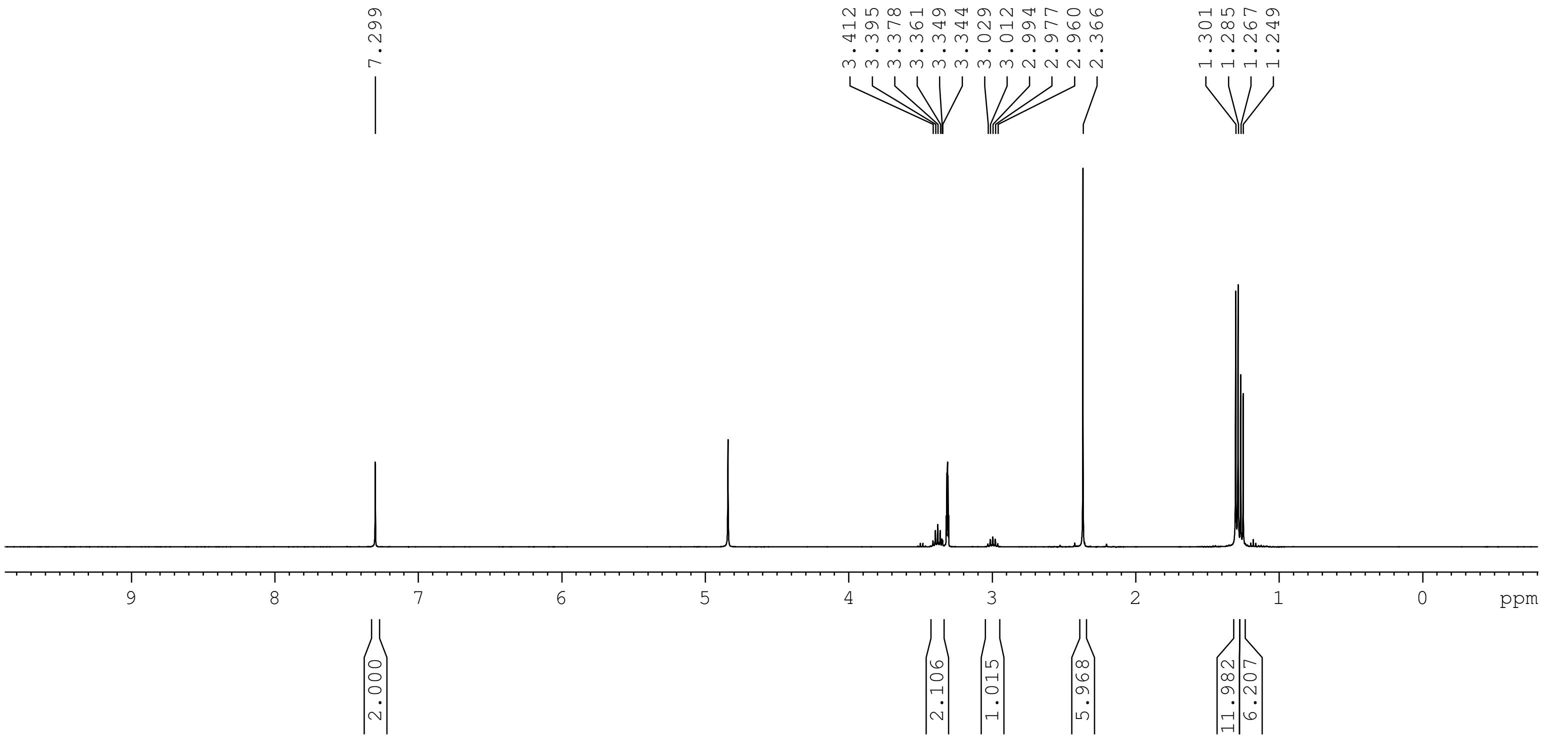

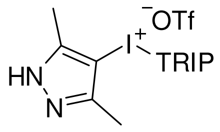

3n

100 MHz
MeOD

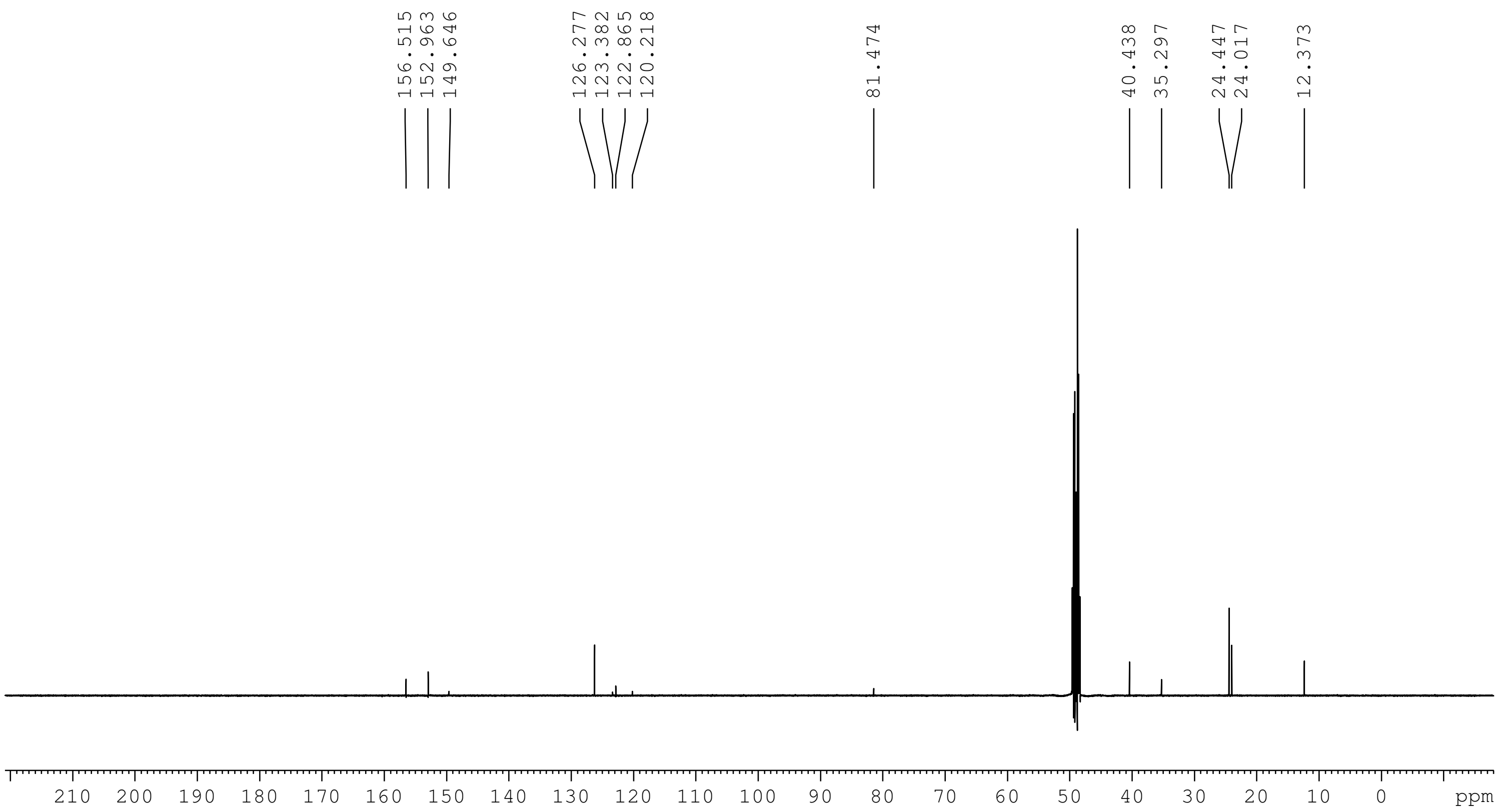

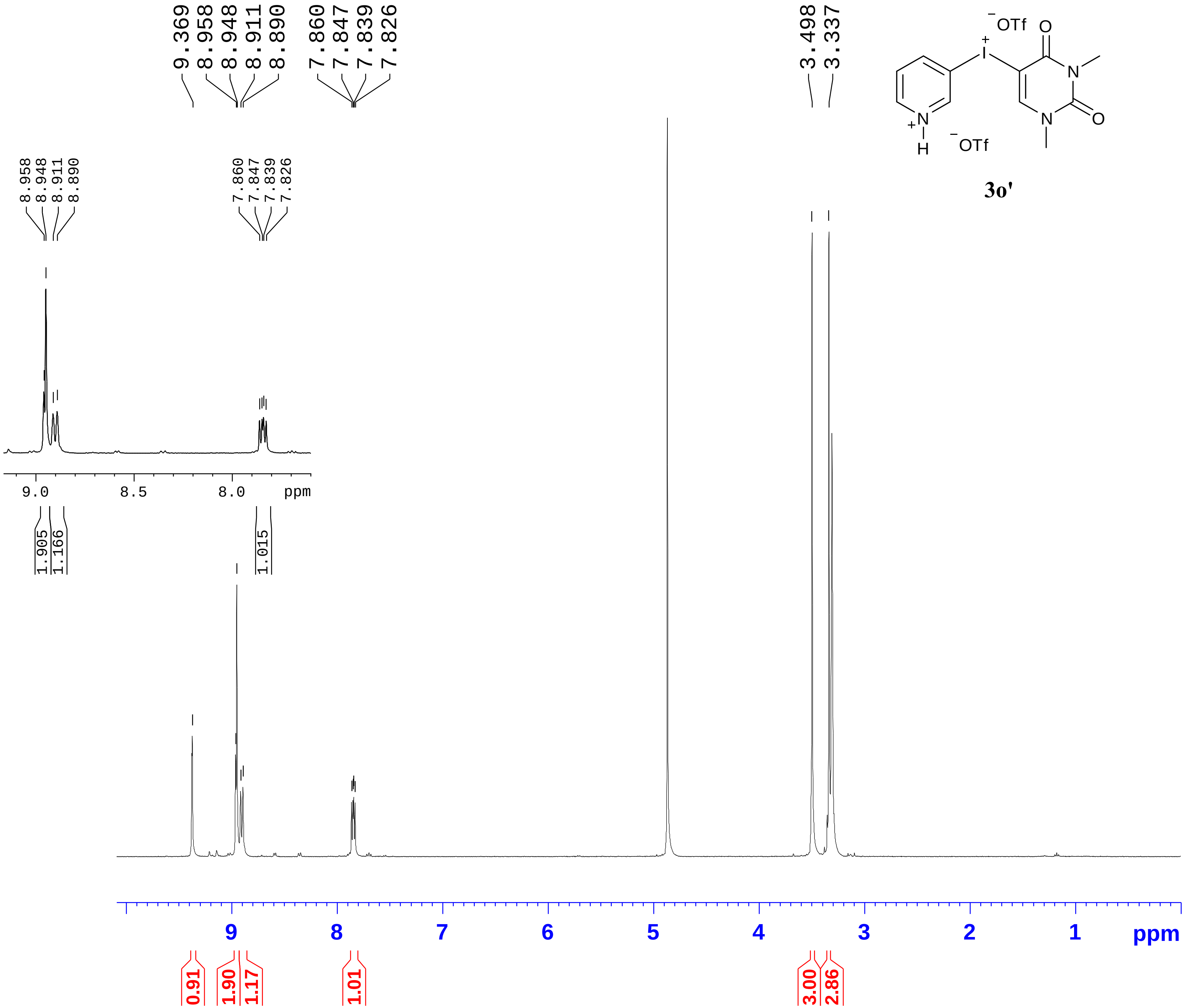

3o'

400 MHz
MeOD

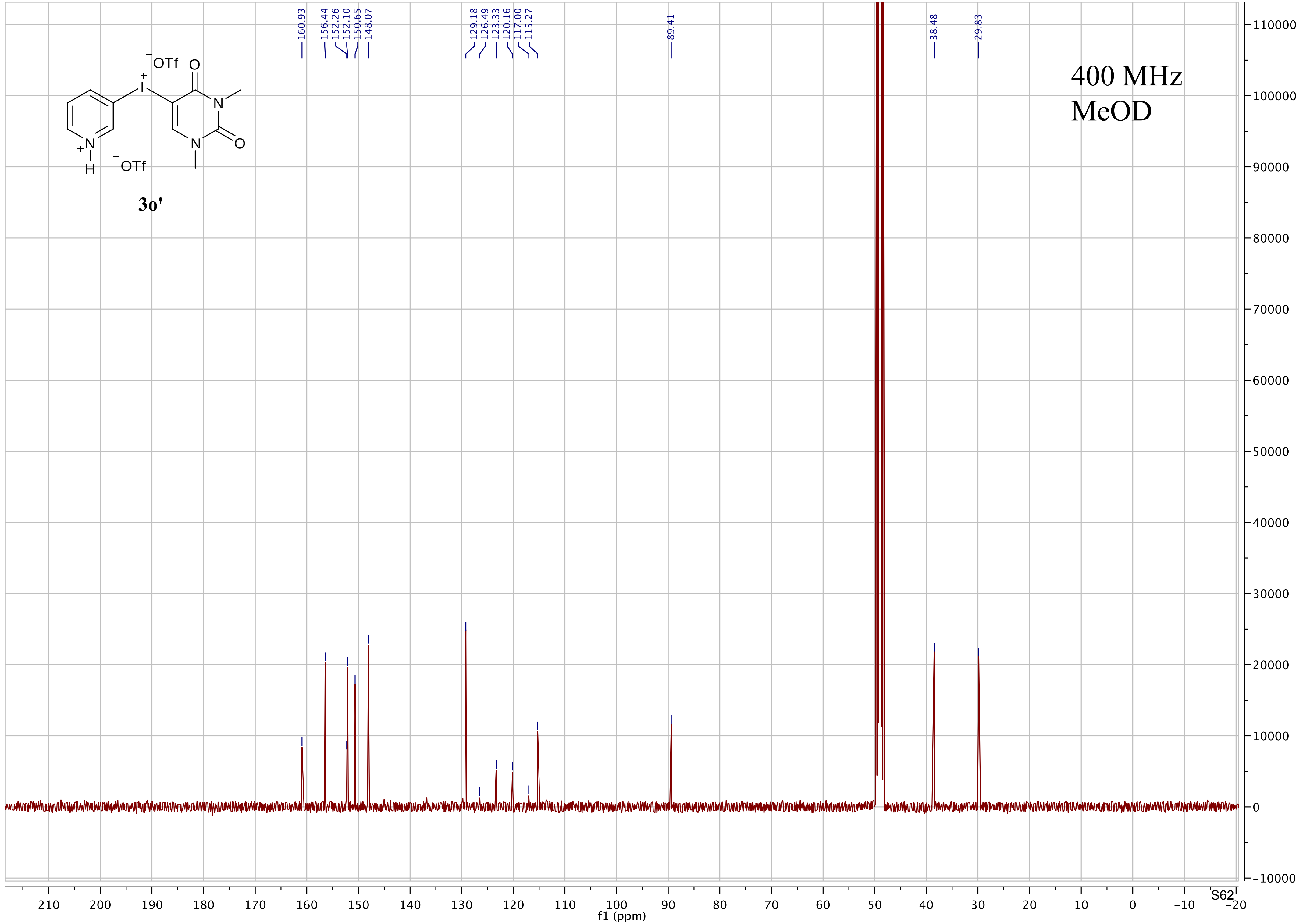

160.93
156.44
152.26
152.10
150.65
148.07
129.18
126.49
123.33
120.16
117.00
115.27
89.41
38.48
29.83
400 MHz
MeOD
OTf
OTf
3o'
f1 (ppm)

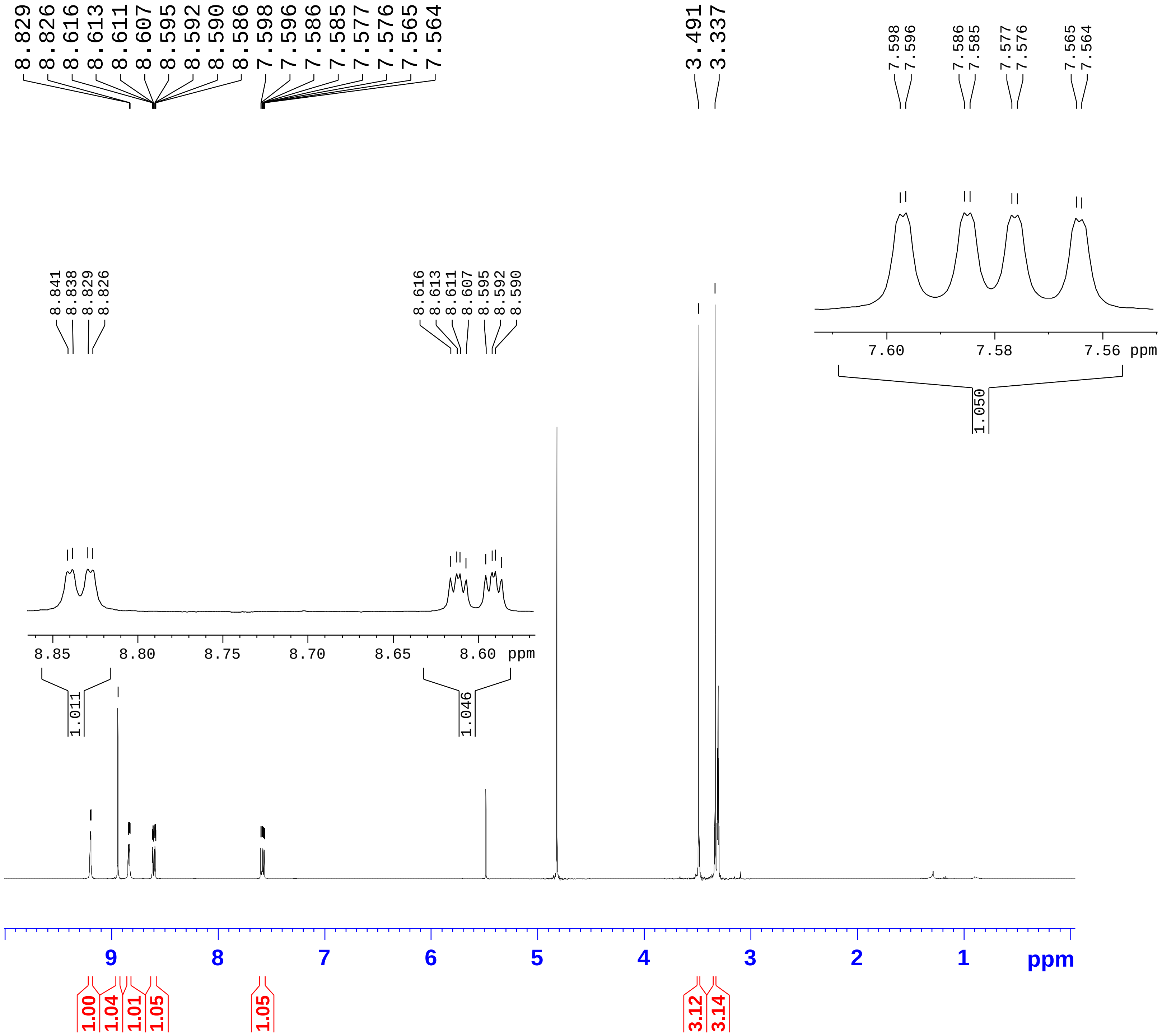

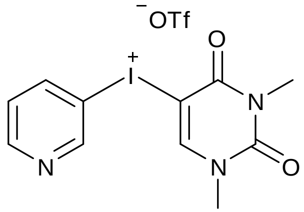

3o

400 MHz
MeOD

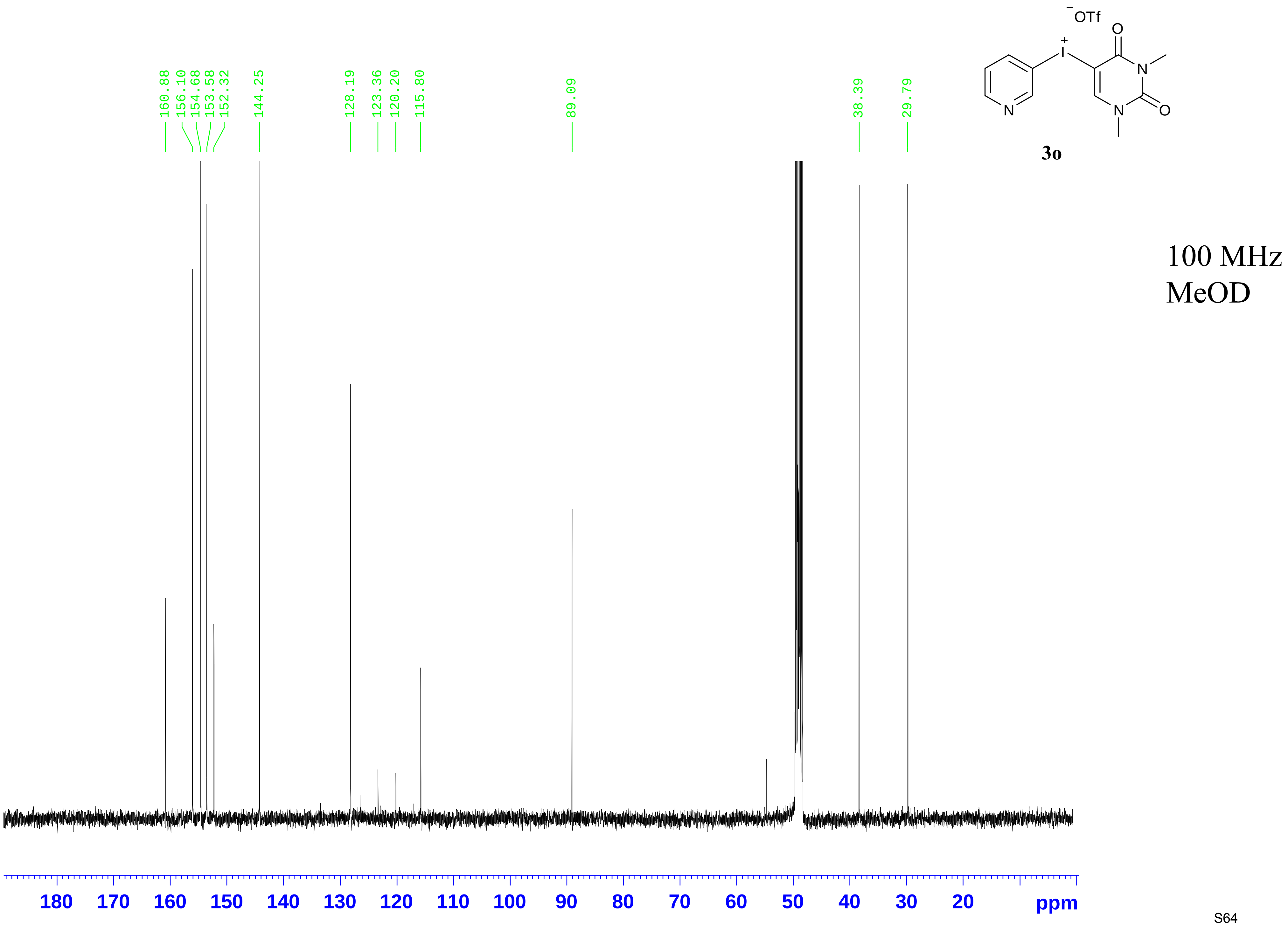
160.88
156.10
154.68
153.58
152.32
144.25
128.19
123.36
120.20
115.80
89.09
38.39
29.79
⁻OTf
I⁺
O
N
O
N
N
100 MHz
MeOD
180
170
160
150
140
130
120
110
100
90
80
70
60
50
40
30
20
ppm

3o

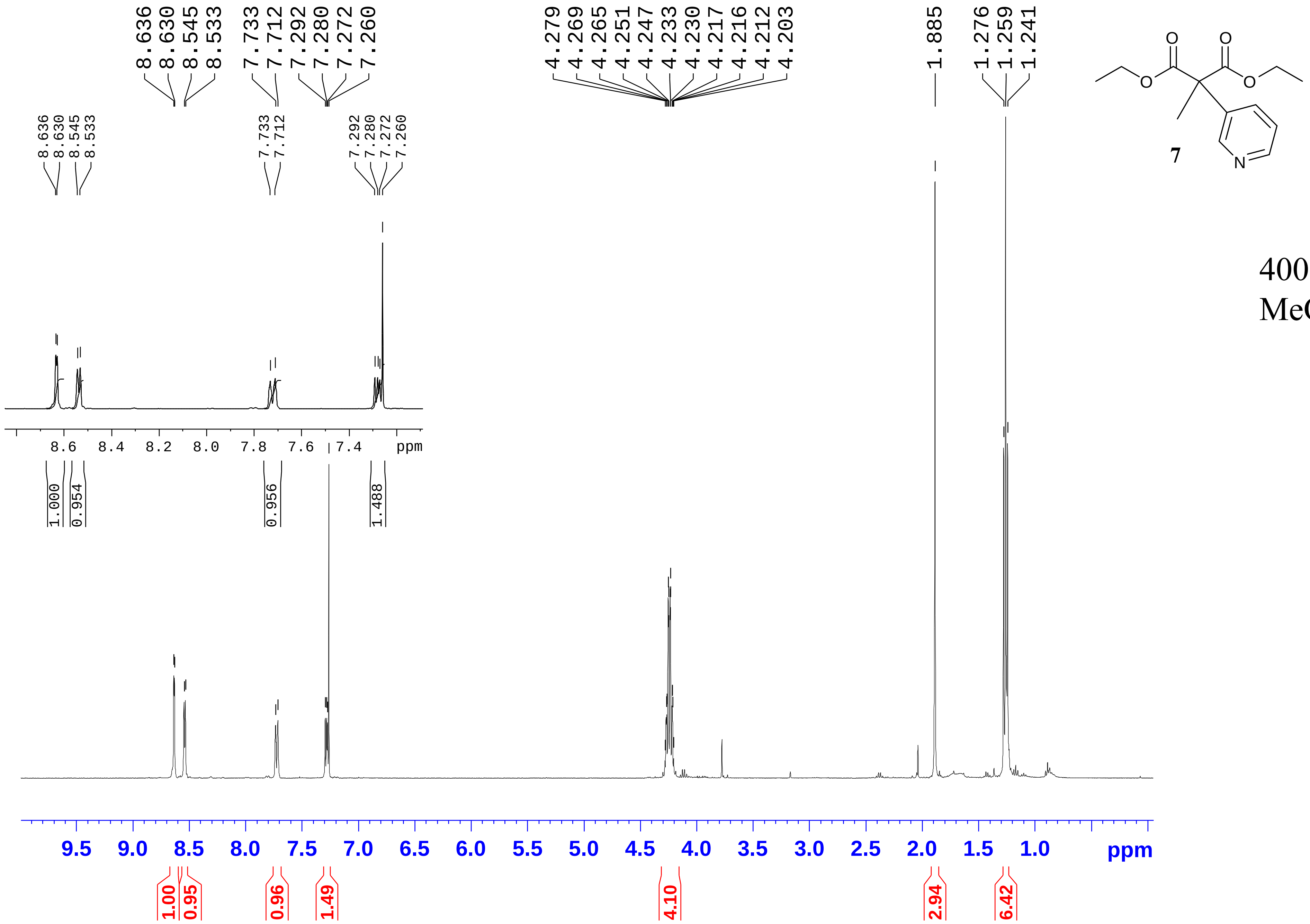
8.636
8.630
8.545
8.533
7.733
7.712
7.292
7.280
7.272
7.260
4.279
4.269
4.265
4.251
4.247
4.233
4.230
4.217
4.216
4.212
4.203
1.885
1.276
1.259
1.241
1.000
0.954
0.956
1.488
ppm
1.00
0.95
0.96
1.49
4.10
2.94
6.42
O
N
7

400 MHz
MeOD

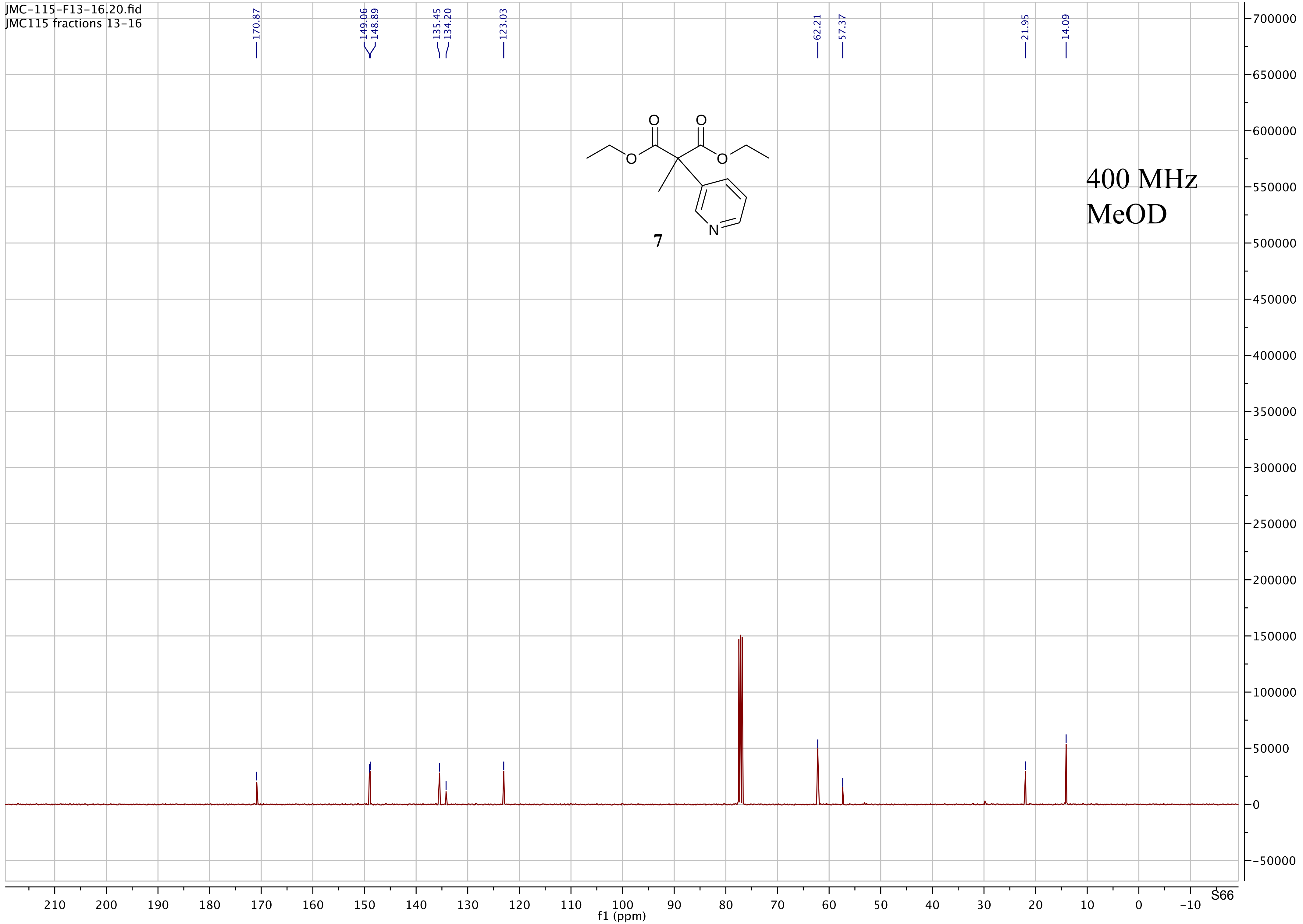
JMC-115-F13-16.20.fid
JMC115 fractions 13-16
170.87
149.06
148.89
135.45
134.20
123.03
62.21
57.37
21.95
14.09
O
O
O
O
N
7
400 MHz
MeOD
f1 (ppm)
